# Supplementary material for: Examination of the Complex Molecular Landscape in Obesity and Type 2 Diabetes
Source: Int J Mol Sci. 2024 Apr 27;25(9):4781. doi: 10.3390/ijms25094781 (PMC11084226; doi:10.3390/ijms25094781)
Supplement: Supplementary file 1 [file ijms-25-04781-s001.zip › FigureS1.pdf]

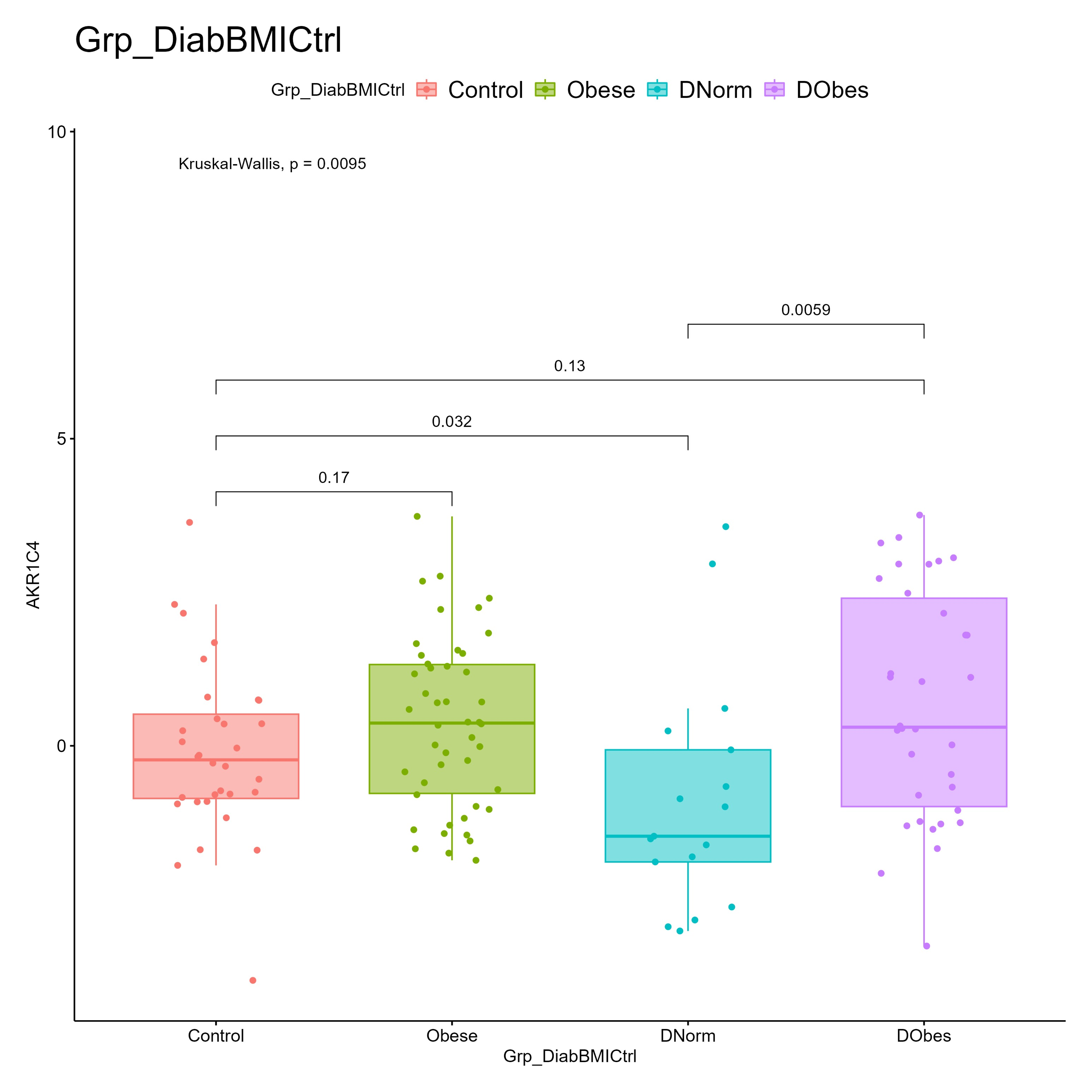

Grp_DiabBMICtrl
Grp_DiabBMICtrl
Control
Obese
DNorm
DObes
Kruskal-Wallis, p = 0.0095
0.0059
0.13
0.032
0.17
10
5
0
AKR1C4
Control
Obese
DNorm
DObes
Grp_DiabBMICtrl

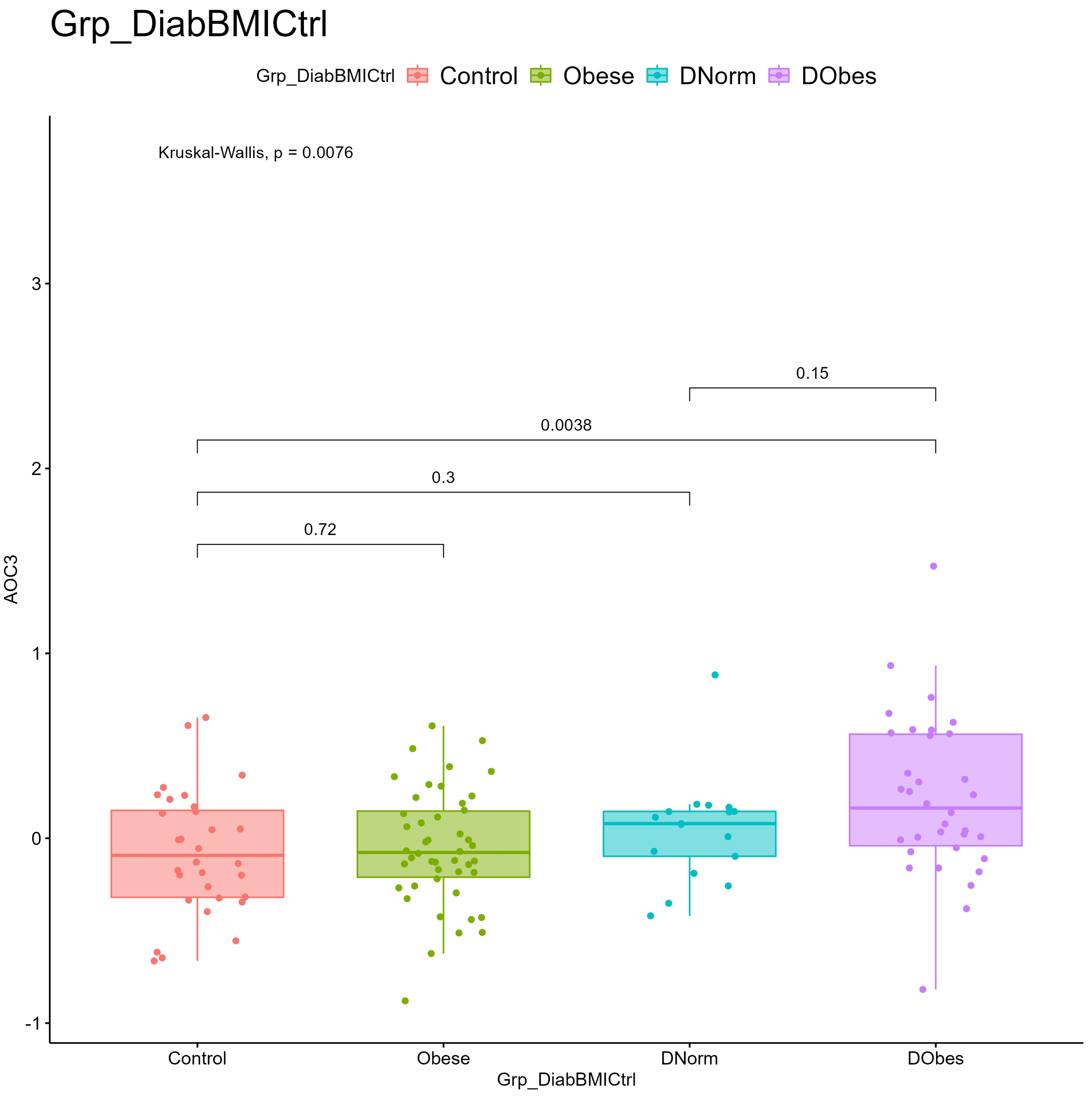
Grp_DiabBMICtrl
Grp_DiabBMICtrl
Control
Obese
DNorm
DObes
Kruskal-Wallis, p = 0.0076
0.15
0.0038
0.3
0.72
AOC3
3
2
1
0
-1
Control
Obese
DNorm
DObes
Grp_DiabBMICtrl

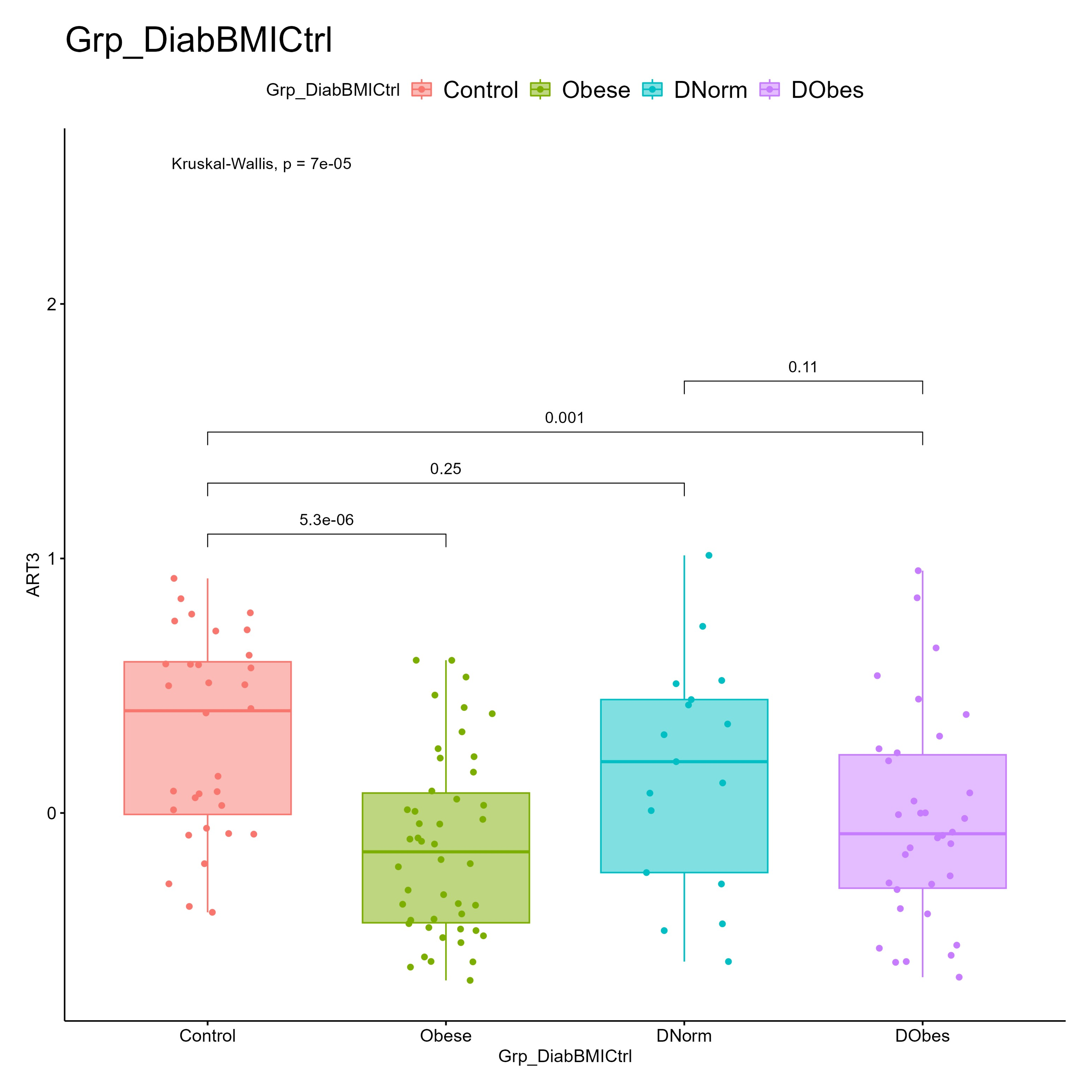

Grp_DiabBMICtrl
Grp_DiabBMICtrl
Control
Obese
DNorm
DObes
Kruskal-Wallis, p = 7e-05
0.11
0.001
0.25
5.3e-06
ART3
2
1
0
Control
Obese
DNorm
DObes
Grp_DiabBMICtrl

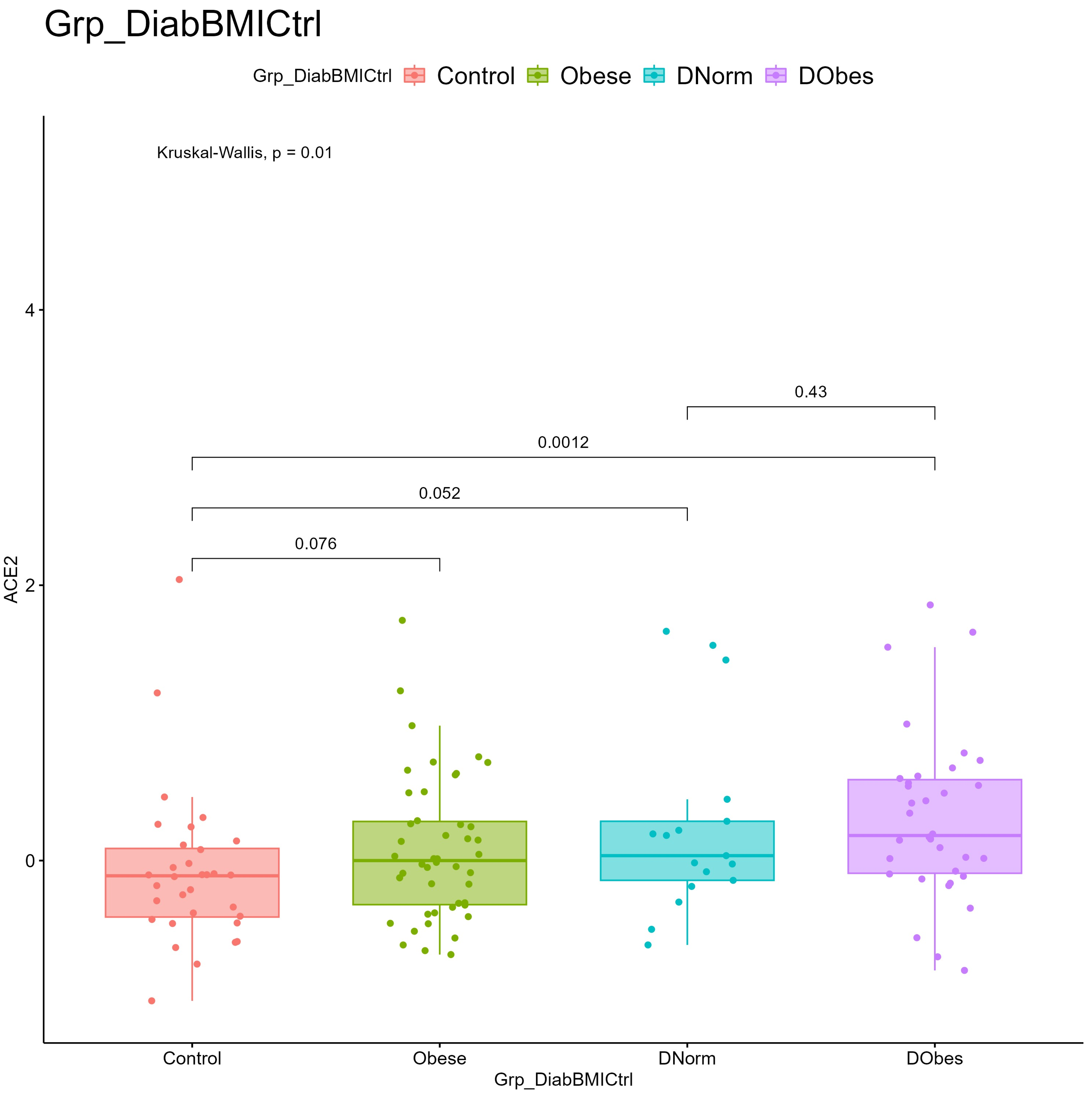

Grp_DiabBMICtrl
Grp_DiabBMICtrl
Control
Obese
DNorm
DObes
Kruskal-Wallis, p = 0.01
0.43
0.0012
0.052
0.076
ACE2
4
2
0
Control
Obese
DNorm
DObes
Grp_DiabBMICtrl

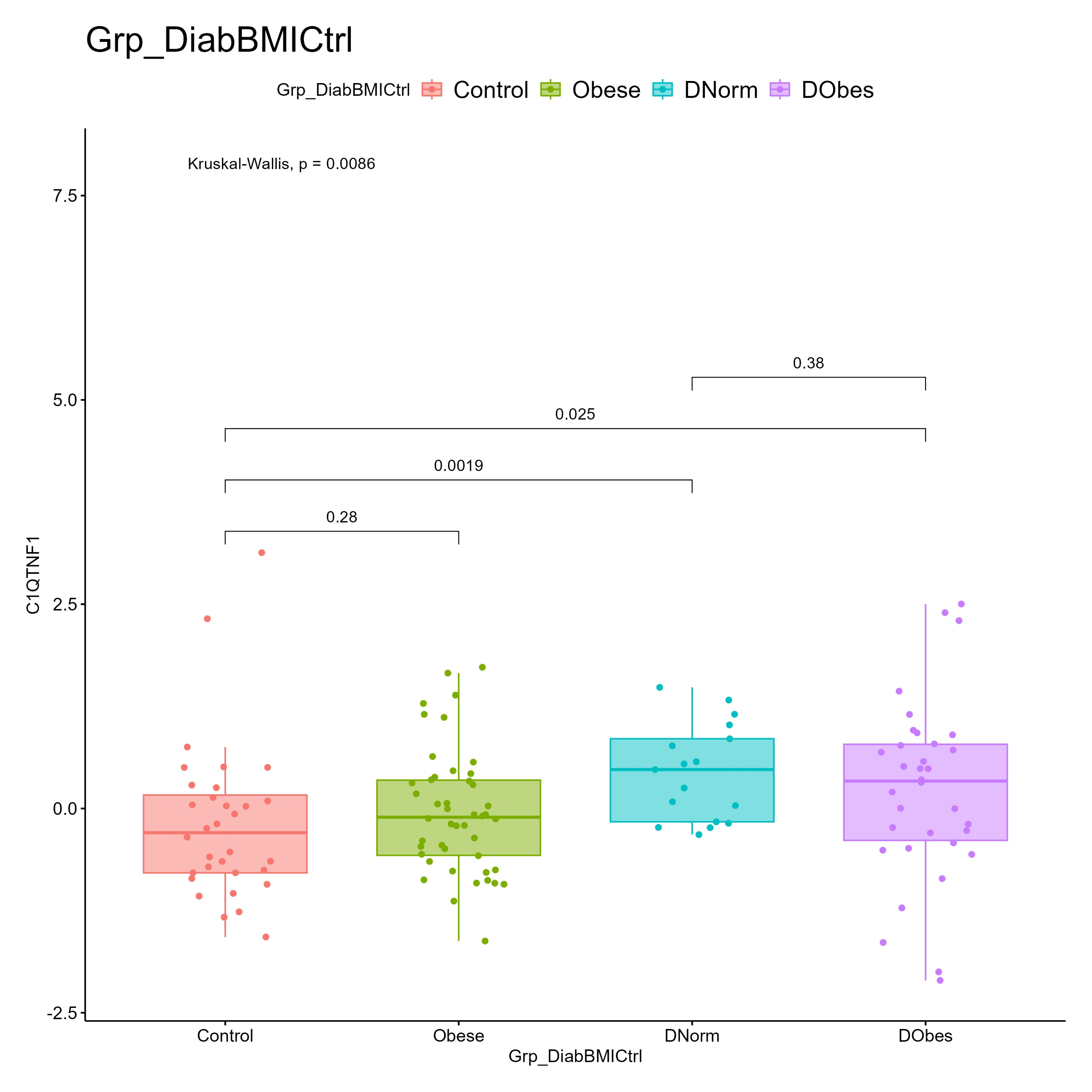
Grp_DiabBMICtrl
Grp_DiabBMICtrl
Control
Obese
DNorm
DObes
Kruskal-Wallis, p = 0.0086
0.38
0.025
0.0019
0.28
7.5
5.0
2.5
0.0
-2.5
C1QTNF1
Control
Obese
DNorm
DObes
Grp_DiabBMICtrl

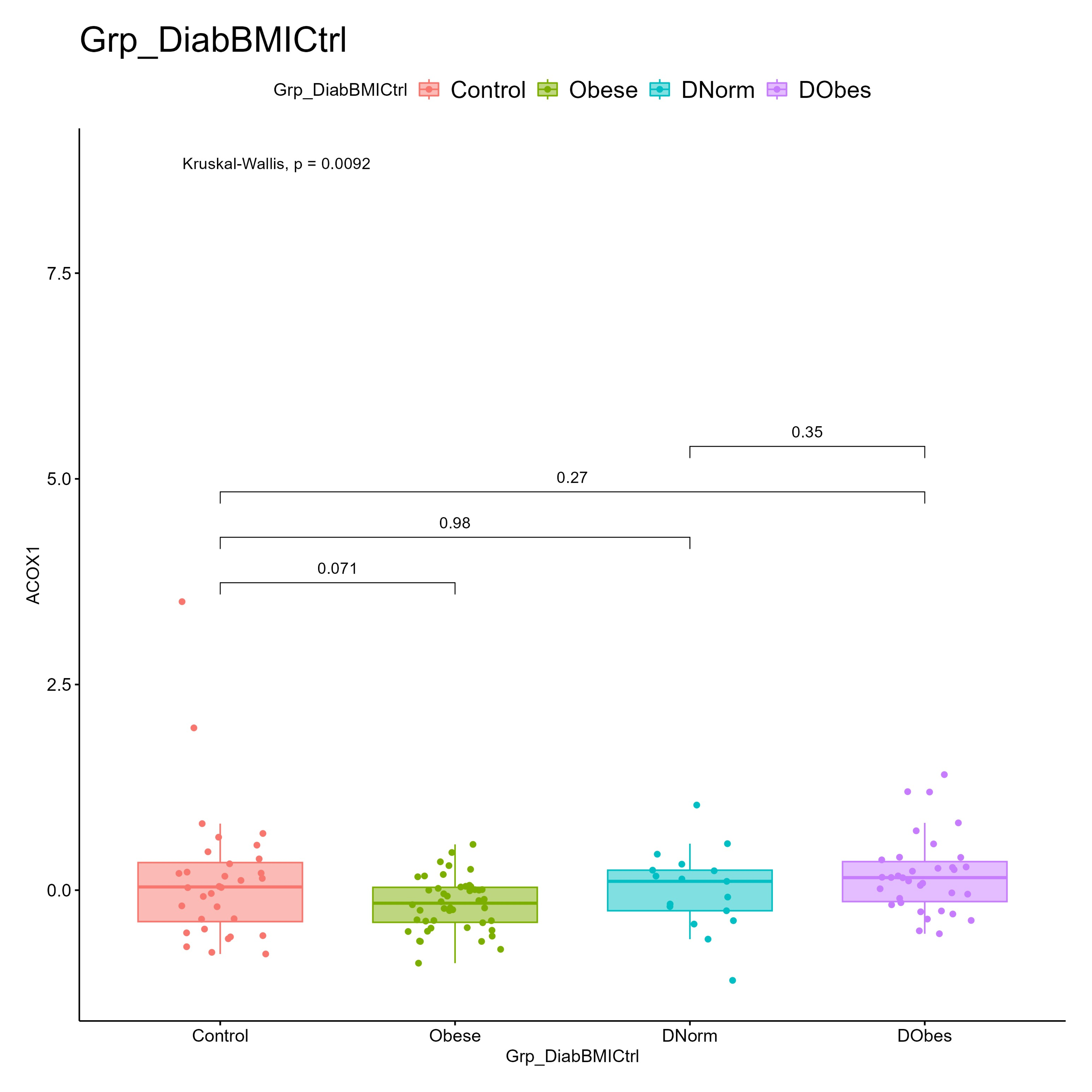

Grp_DiabBMICtrl
Grp_DiabBMICtrl
Control
Obese
DNorm
DObes
Kruskal-Wallis, p = 0.0092
0.35
0.27
0.98
0.071
7.5
5.0
2.5
0.0
ACOX1
Control
Obese
DNorm
DObes
Grp_DiabBMICtrl

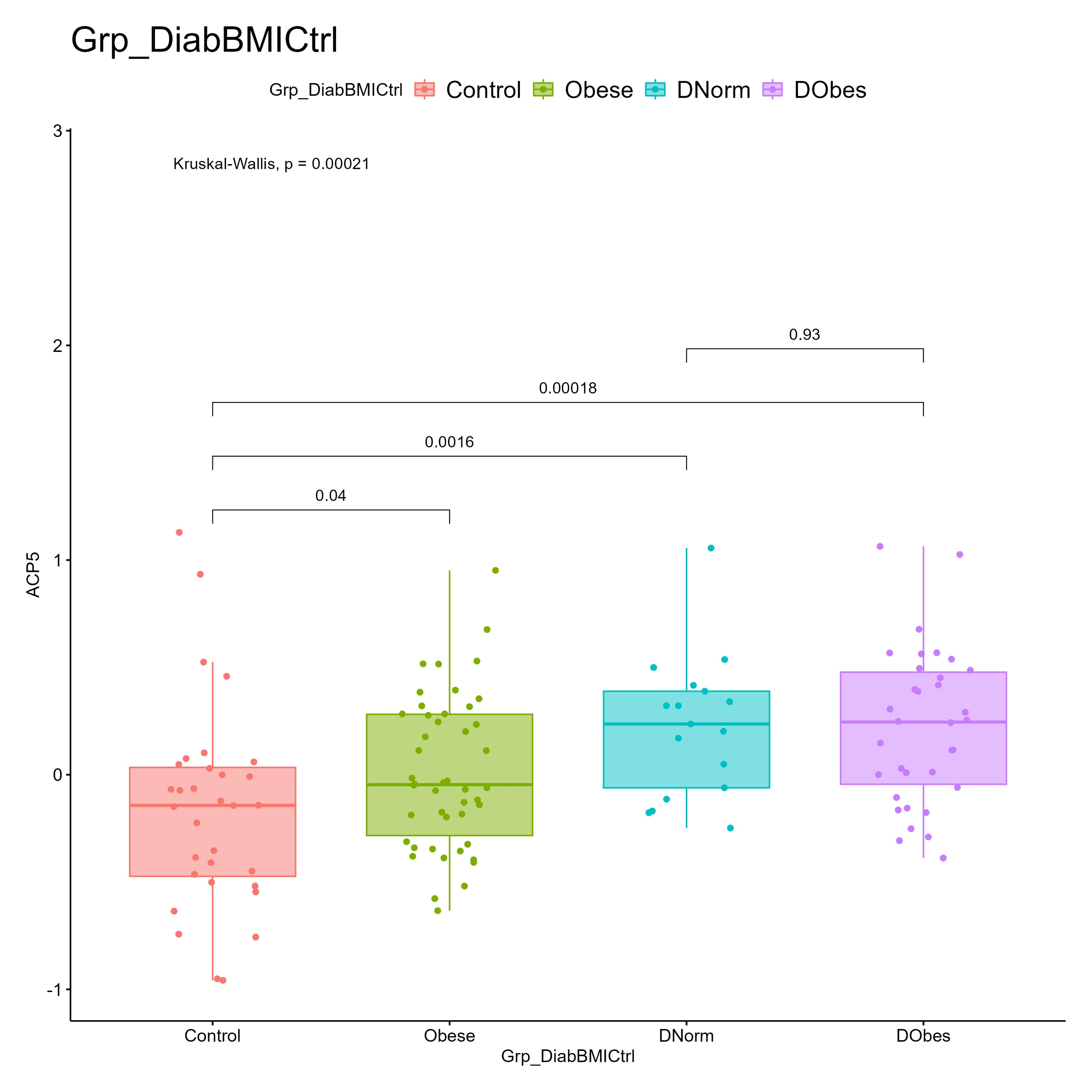

Grp_DiabBMICtrl
Grp_DiabBMICtrl
Control
Obese
DNorm
DObes
Kruskal-Wallis, p = 0.00021
0.93
0.00018
0.0016
0.04
3
2
1
0
-1
ACP5
Control
Obese
DNorm
DObes
Grp_DiabBMICtrl

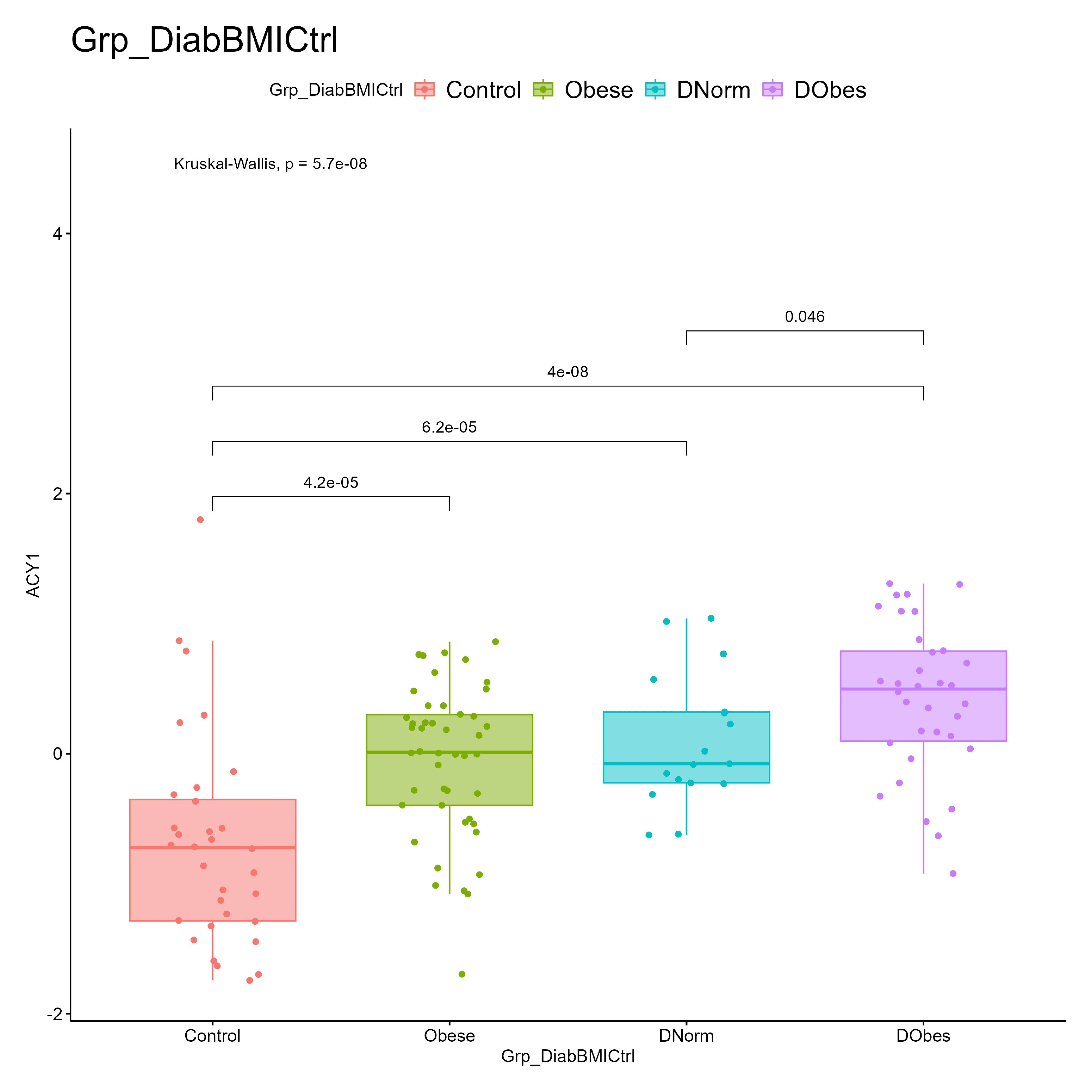
Grp_DiabBMICtrl
Grp_DiabBMICtrl
Control
Obese
DNorm
DObes
Kruskal-Wallis, p = 5.7e-08
0.046
4e-08
6.2e-05
4.2e-05
ACY1
4
2
0
-2
Control
Obese
DNorm
DObes
Grp_DiabBMICtrl

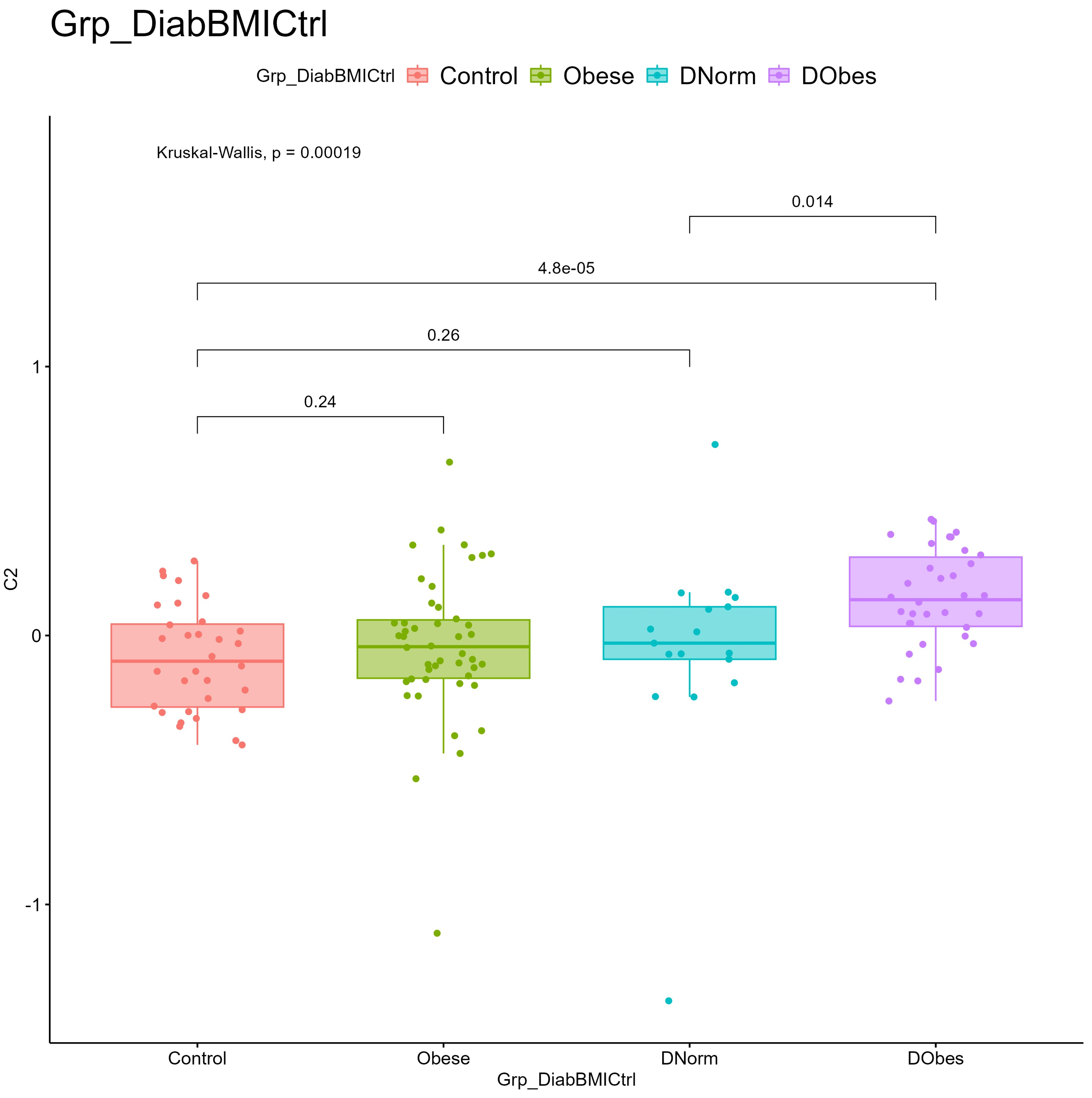
Grp_DiabBMICtrl
Grp_DiabBMICtrl
Control
Obese
DNorm
DObes
Kruskal-Wallis, p = 0.00019
0.014
4.8e-05
0.26
0.24
1
0
-1
C2
Control
Obese
DNorm
DObes
Grp_DiabBMICtrl

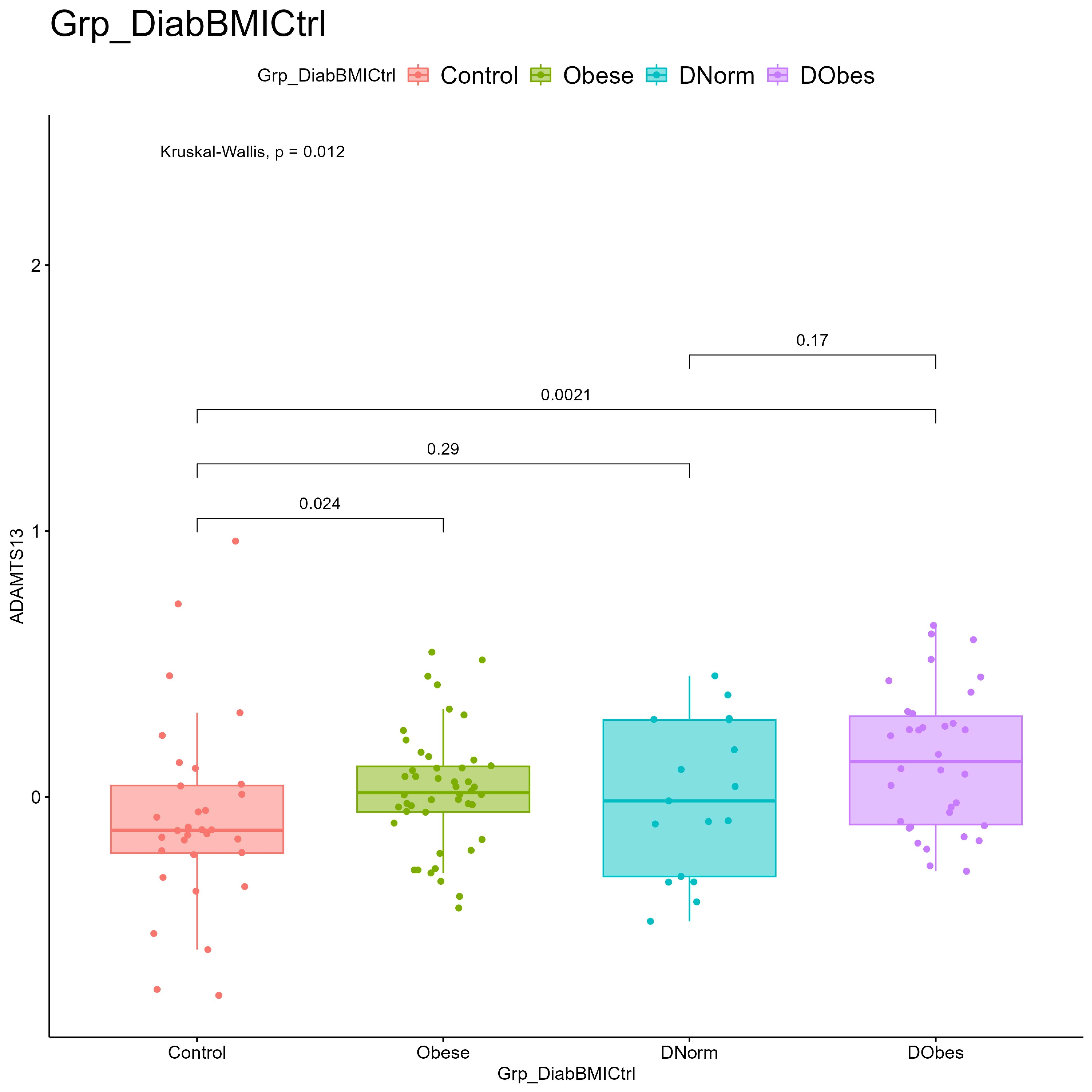
Grp_DiabBMICtrl
Grp_DiabBMICtrl
Control
Obese
DNorm
DObes
Kruskal-Wallis, p = 0.012
0.17
0.0021
0.29
0.024
ADAMTS13
2
1
0
Control
Obese
DNorm
DObes
Grp_DiabBMICtrl

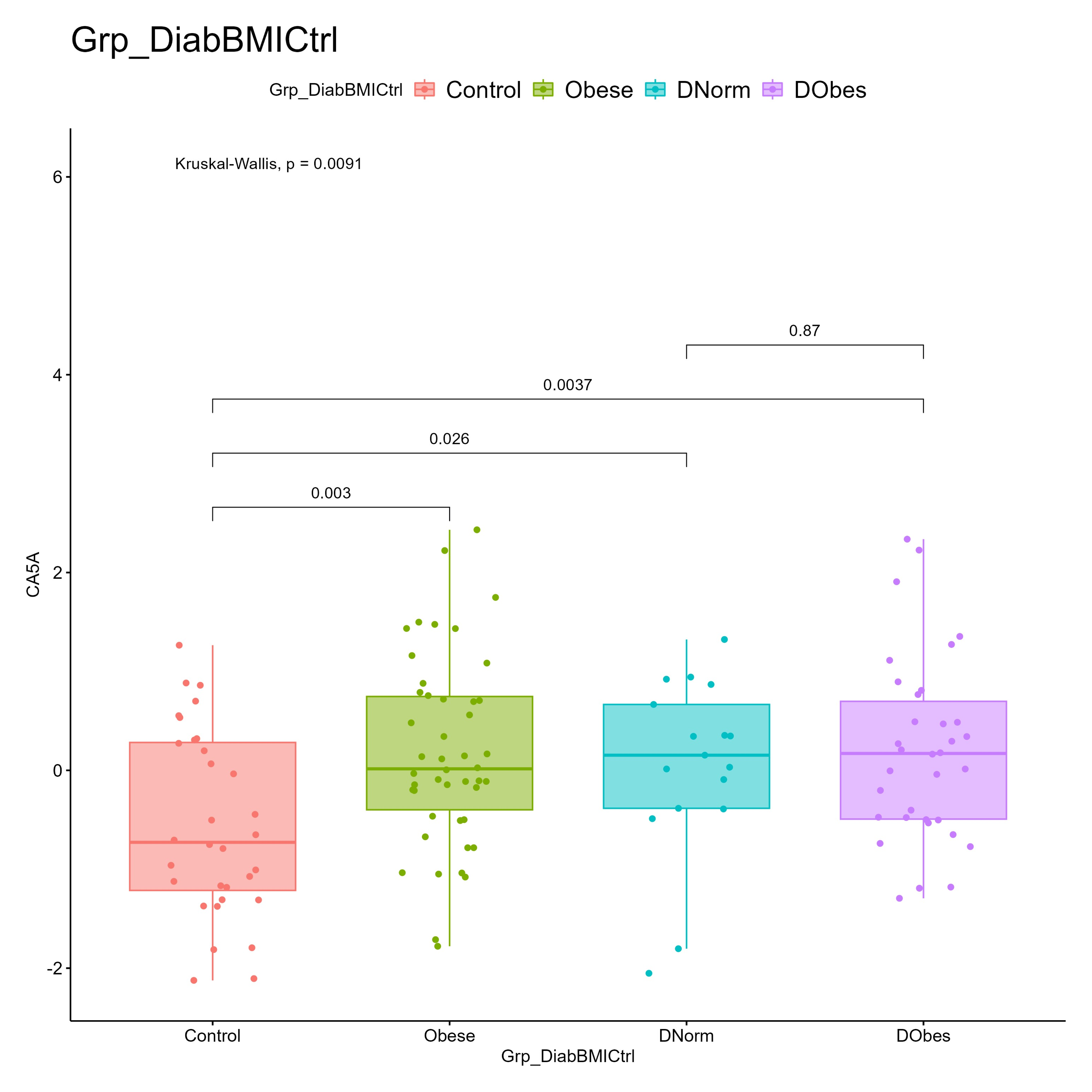
Grp_DiabBMICtrl
Grp_DiabBMICtrl Control Obese DNorm DObes
Kruskal-Wallis, p = 0.0091
0.87
0.0037
0.026
0.003
6
4
2
0
-2
CA5A
Control
Obese
DNorm
DObes
Grp_DiabBMICtrl

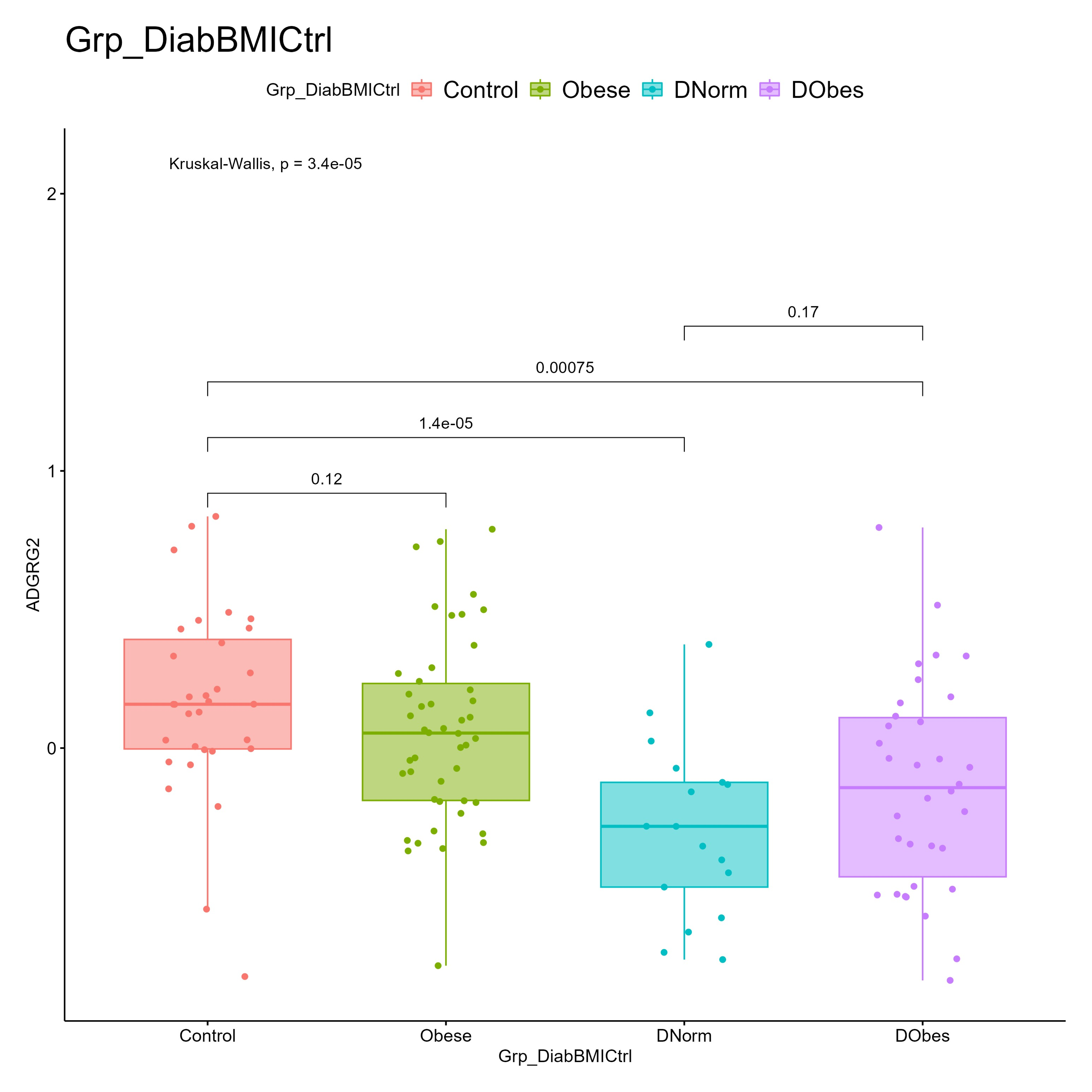

Grp_DiabBMICtrl
Grp_DiabBMICtrl
Control
Obese
DNorm
DObes
Kruskal-Wallis, p = 3.4e-05
0.17
0.00075
1.4e-05
0.12
ADGRG2
2
1
0
Control
Obese
DNorm
DObes
Grp_DiabBMICtrl

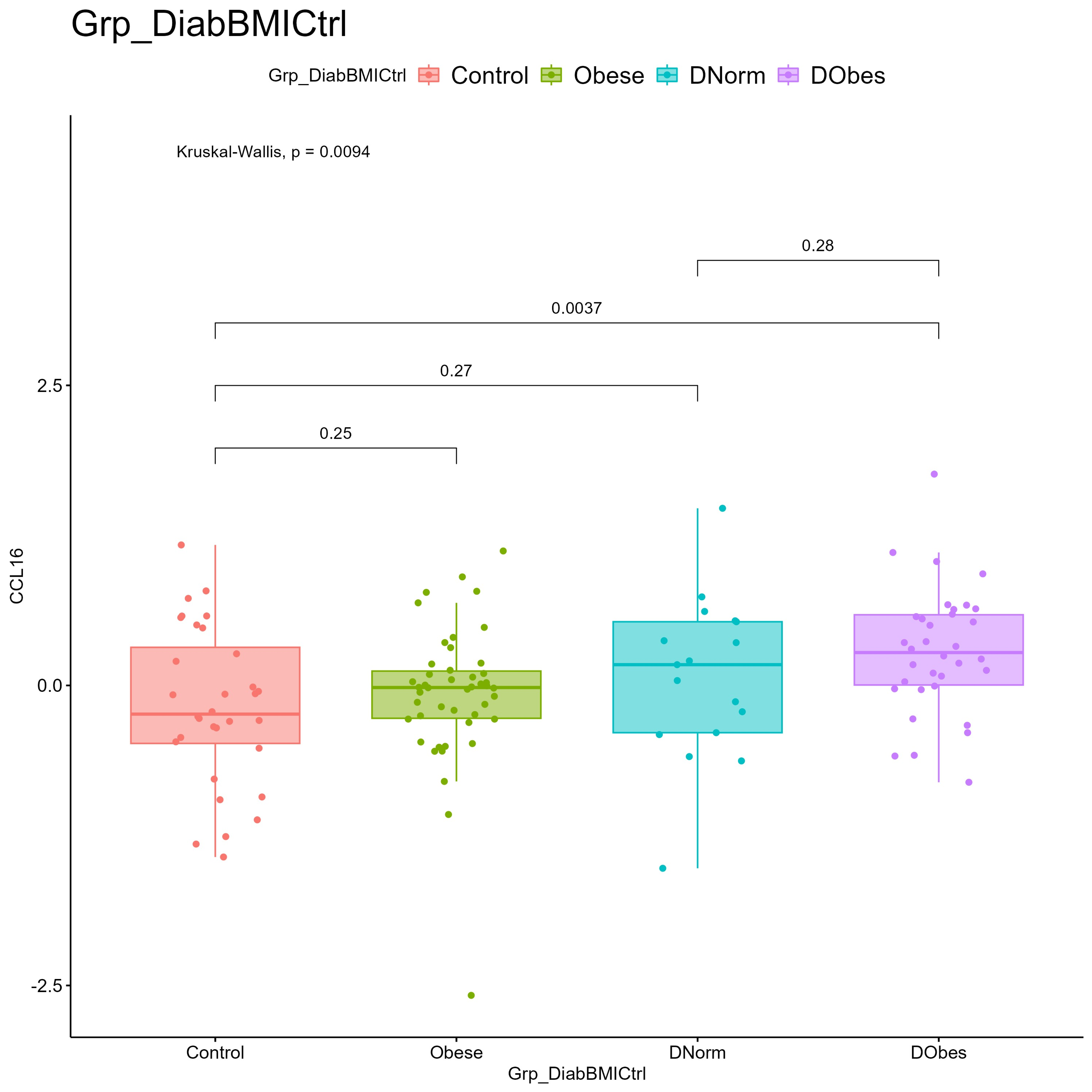
Grp_DiabBMICtrl
Grp_DiabBMICtrl
Control
Obese
DNorm
DObes
Kruskal-Wallis, p = 0.0094
0.28
0.0037
0.27
0.25
2.5
0.0
-2.5
CCL16
Control
Obese
DNorm
DObes
Grp_DiabBMICtrl

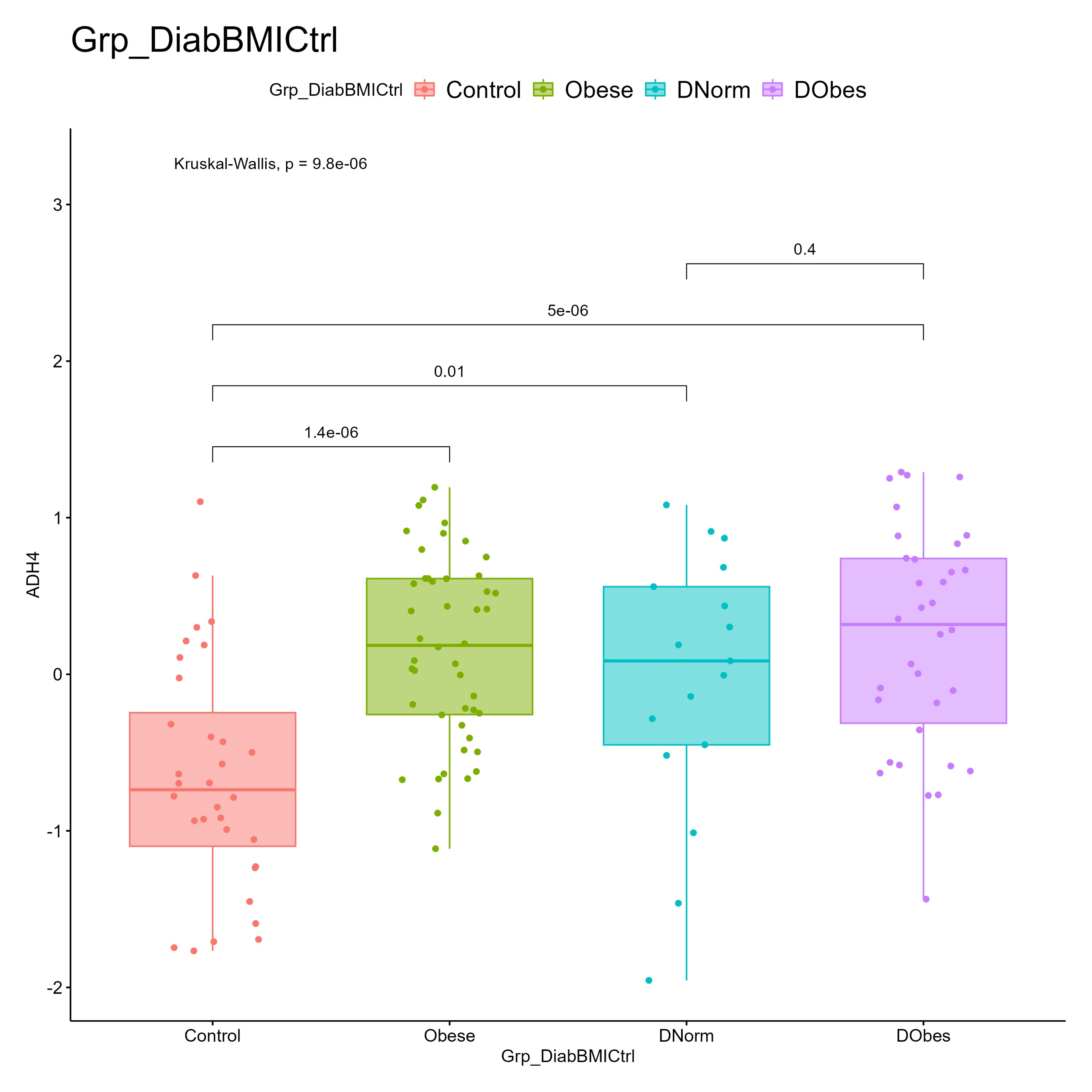

Grp_DiabBMICtrl
Grp_DiabBMICtrl
Control
Obese
DNorm
DObes
Kruskal-Wallis, p = 9.8e-06
0.4
5e-06
0.01
1.4e-06
ADH4
3
2
1
0
-1
-2
Control
Obese
DNorm
DObes
Grp_DiabBMICtrl

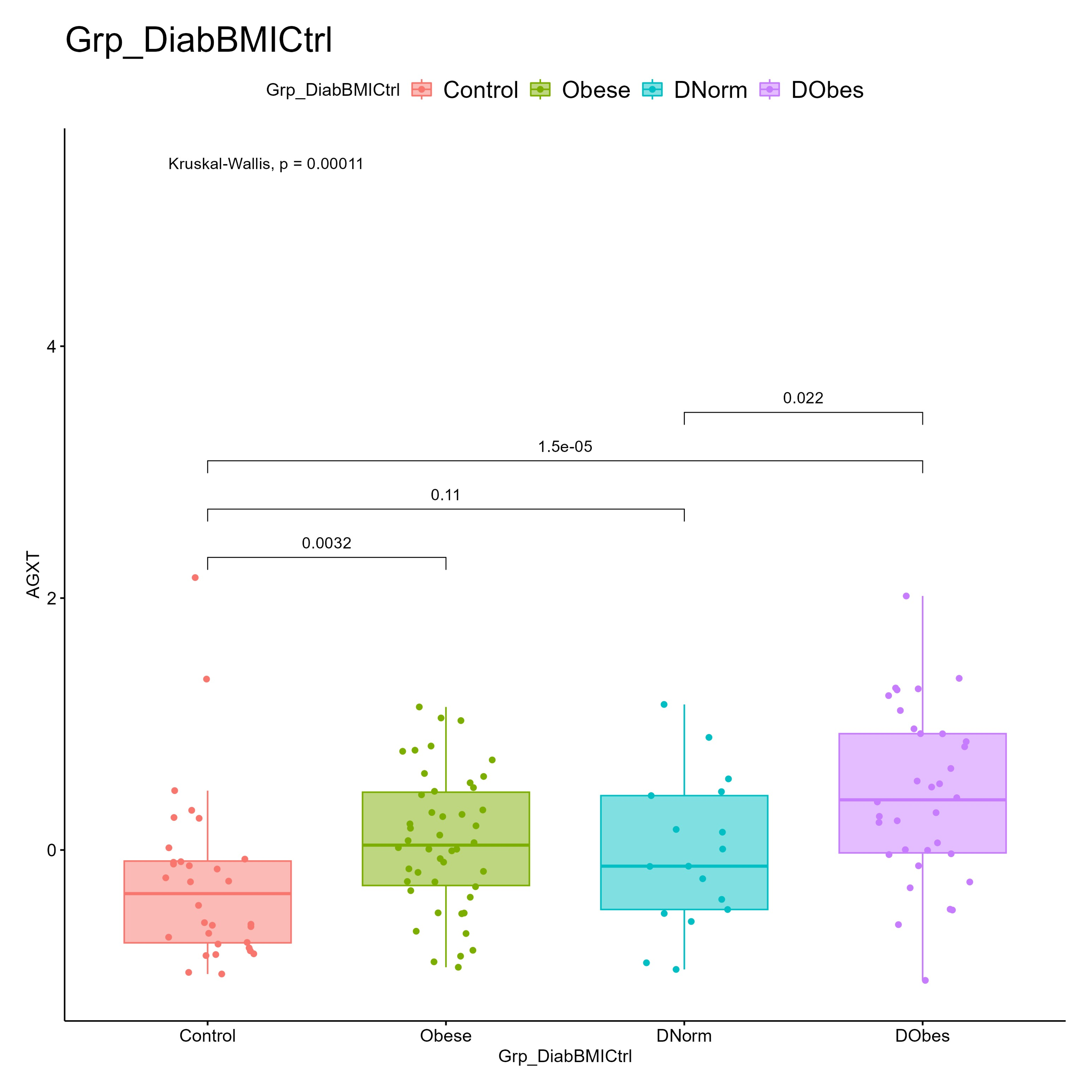

Grp_DiabBMICtrl
Grp_DiabBMICtrl
Control
Obese
DNorm
DObes
Kruskal-Wallis, p = 0.00011
0.022
1.5e-05
0.11
0.0032
AGXT
4
2
0
Control
Obese
DNorm
DObes
Grp_DiabBMICtrl

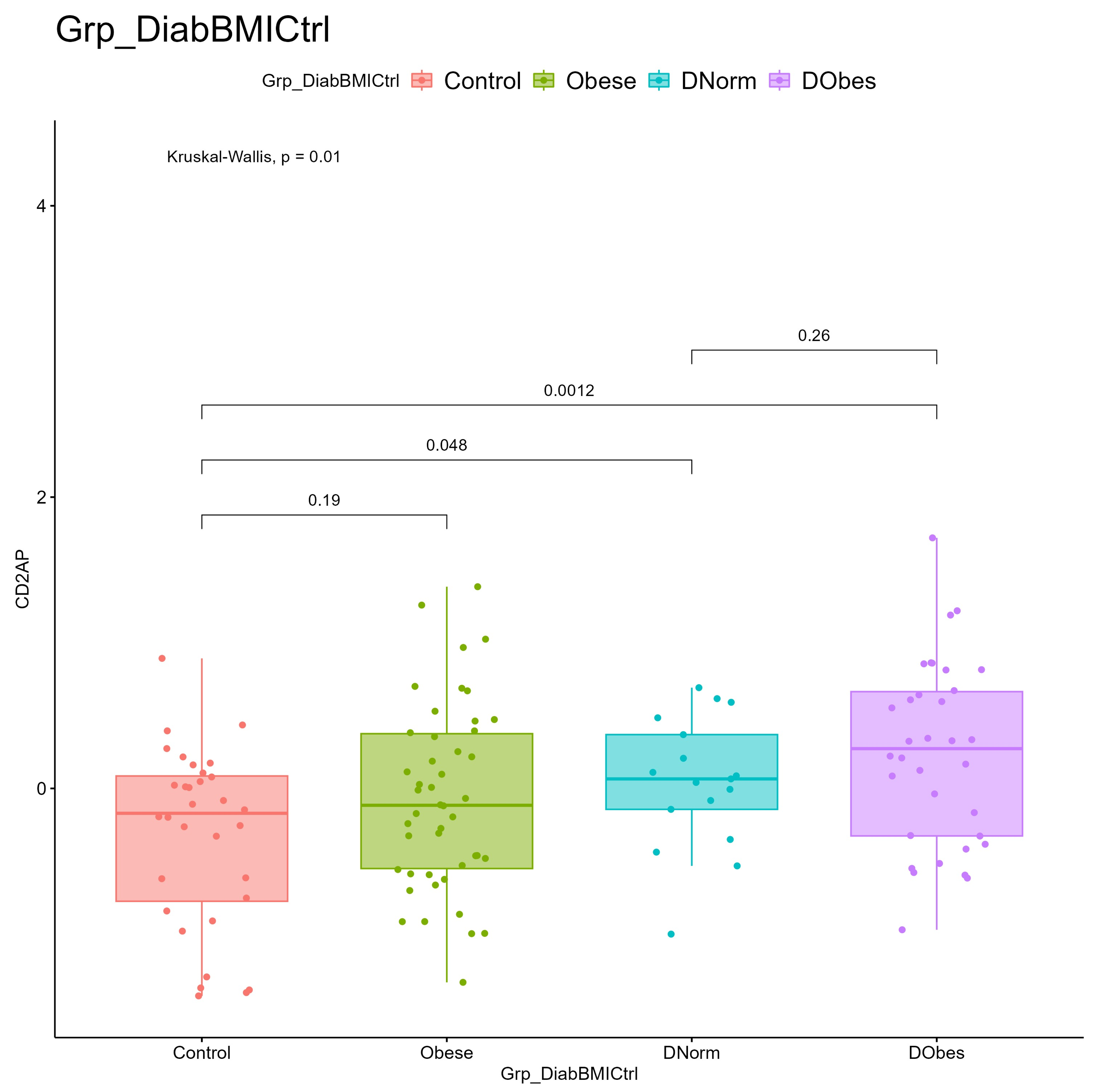

Grp_DiabBMICtrl
Grp_DiabBMICtrl
Control
Obese
DNorm
DObes
Kruskal-Wallis, p = 0.01
0.26
0.0012
0.048
0.19
4
2
0
CD2AP
Control
Obese
DNorm
DObes
Grp_DiabBMICtrl

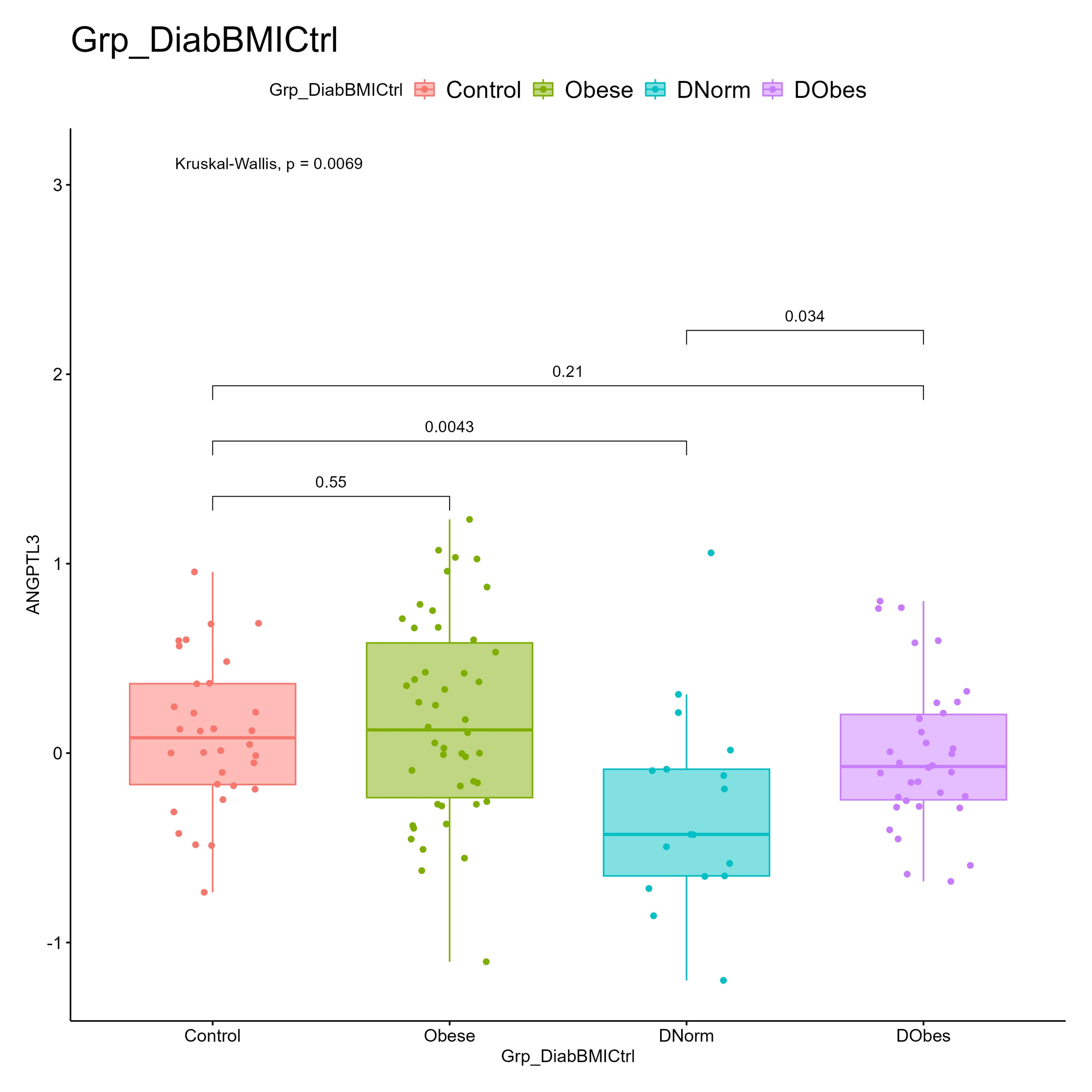

Grp_DiabBMICtrl
Grp_DiabBMICtrl
Control
Obese
DNorm
DObes
Kruskal-Wallis, p = 0.0069
0.034
0.21
0.0043
0.55
ANGPTL3
3
2
1
0
-1
Control
Obese
DNorm
DObes
Grp_DiabBMICtrl

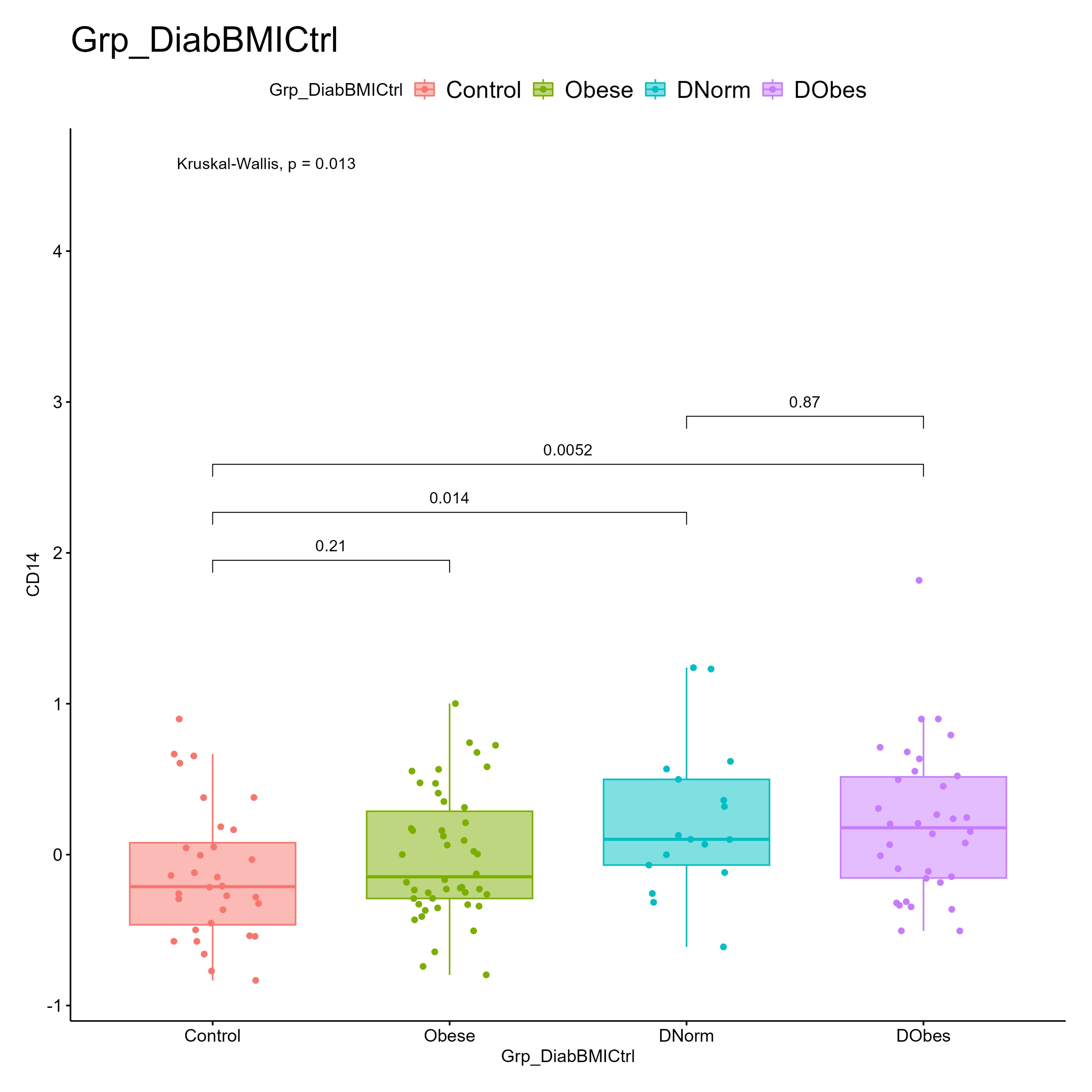
Grp_DiabBMICtrl
Grp_DiabBMICtrl
Control
Obese
DNorm
DObes
Kruskal-Wallis, p = 0.013
0.87
0.0052
0.014
0.21
CD14
4
3
2
1
0
-1
Control
Obese
DNorm
DObes
Grp_DiabBMICtrl

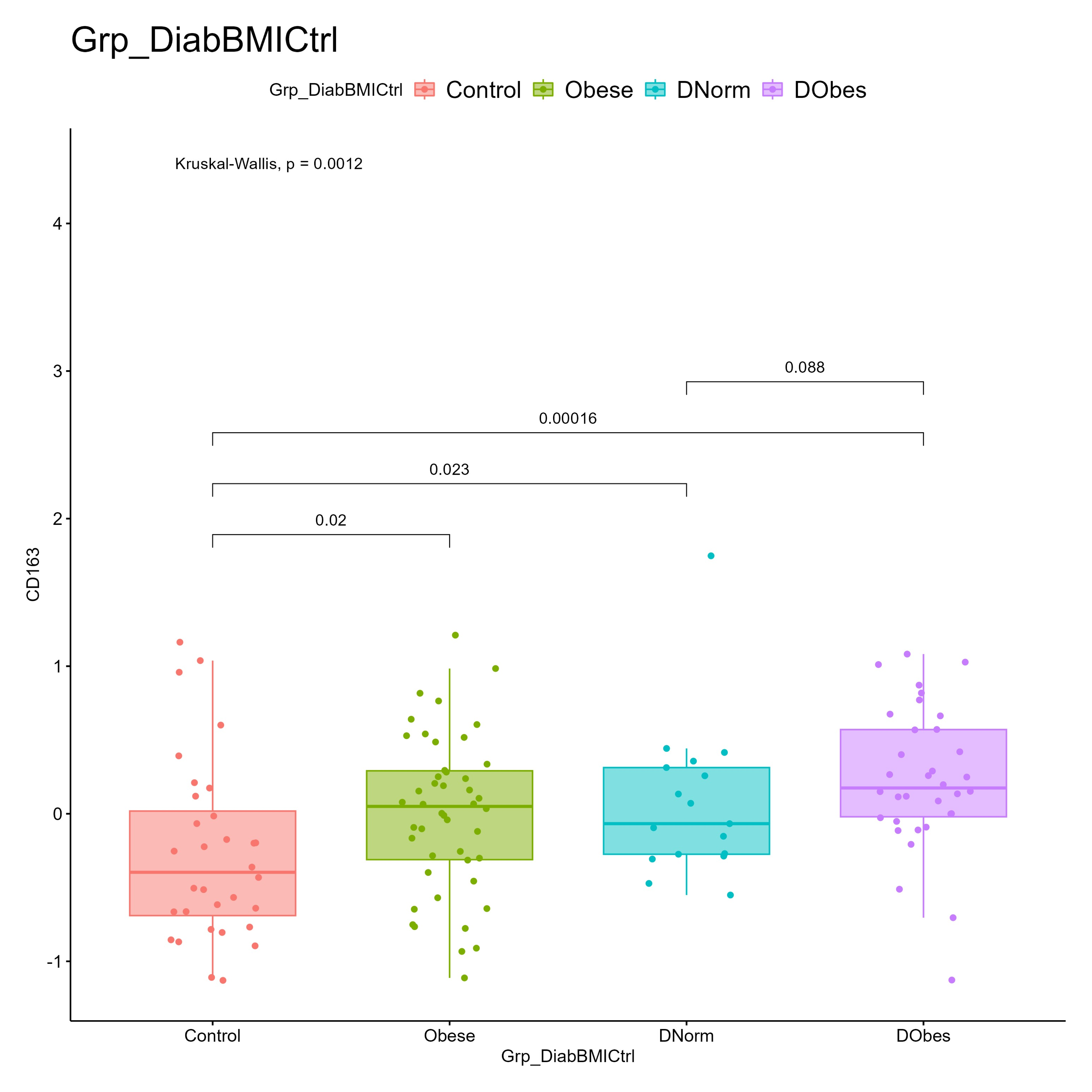
Grp_DiabBMICtrl
Grp_DiabBMICtrl
Control
Obese
DNorm
DObes
Kruskal-Wallis, p = 0.0012
0.088
0.00016
0.023
0.02
CD163
4
3
2
1
0
-1
Control
Obese
DNorm
DObes
Grp_DiabBMICtrl

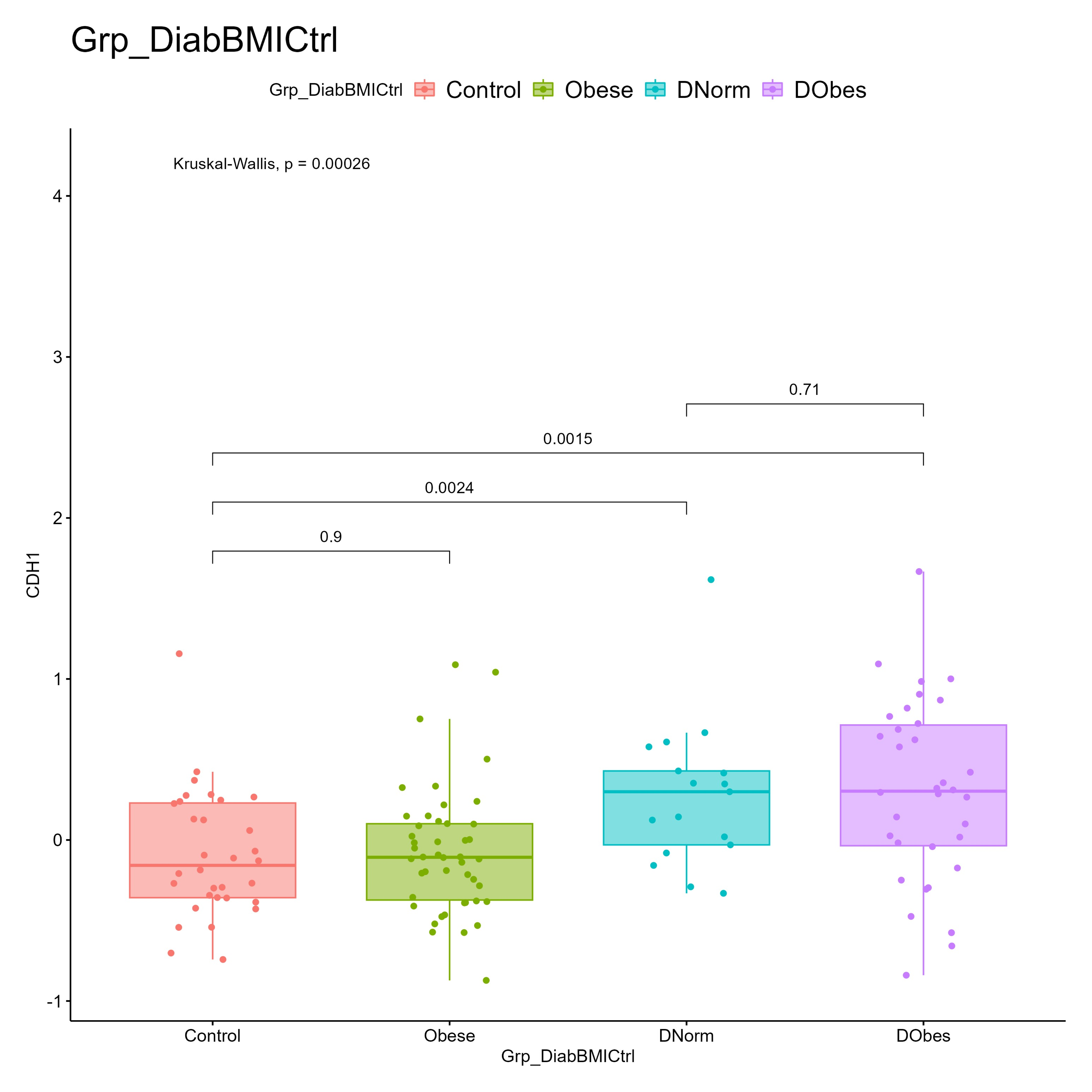
Grp_DiabBMICtrl
Grp_DiabBMICtrl
Control
Obese
DNorm
DObes
Kruskal-Wallis, p = 0.00026
0.71
0.0015
0.0024
0.9
CDH1
4
3
2
1
0
-1
Control
Obese
DNorm
DObes
Grp_DiabBMICtrl

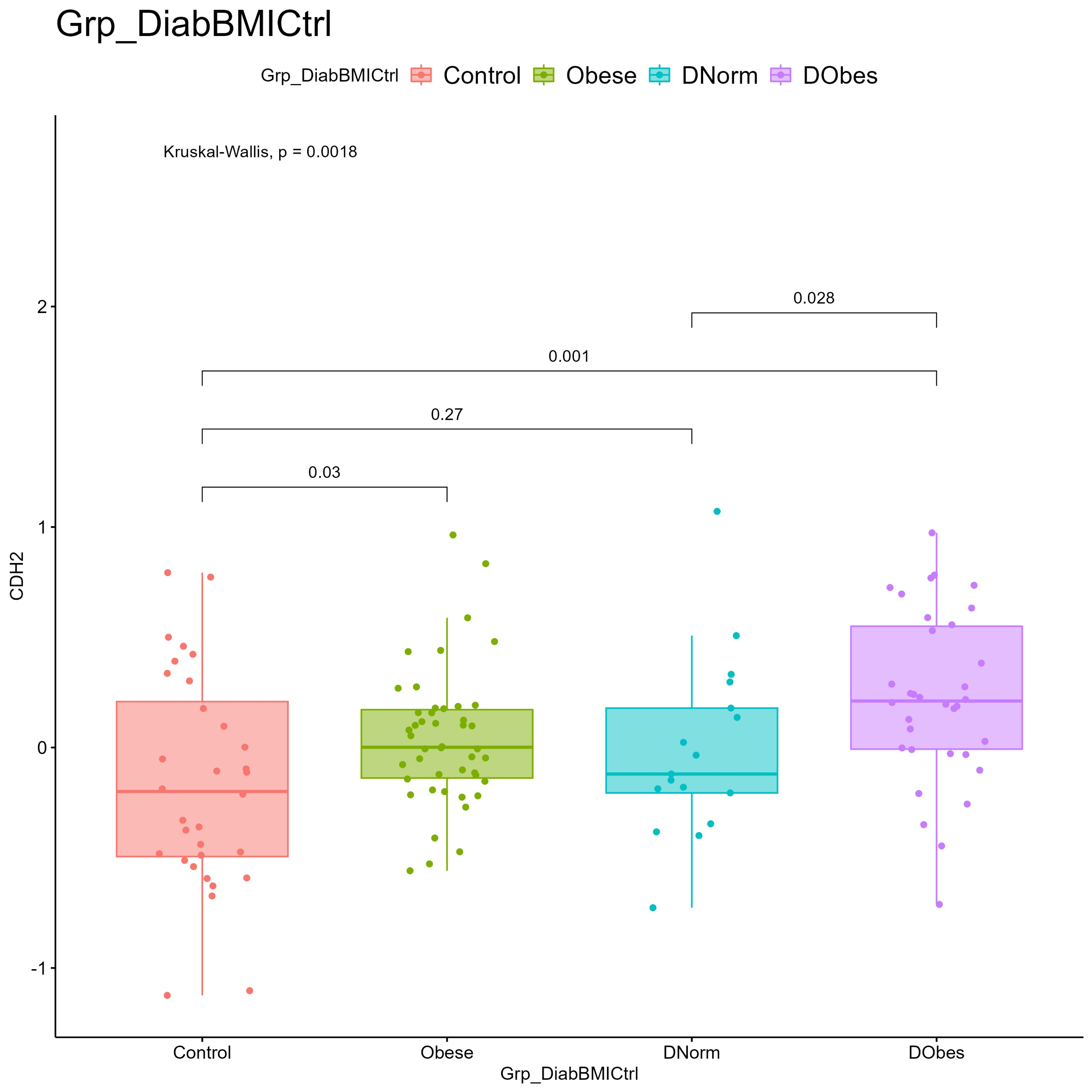
Grp_DiabBMICtrl
Grp_DiabBMICtrl
Control
Obese
DNorm
DObes
Kruskal-Wallis, p = 0.0018
0.028
0.001
0.27
0.03
2
1
0
-1
CDH2
Control
Obese
DNorm
DObes
Grp_DiabBMICtrl

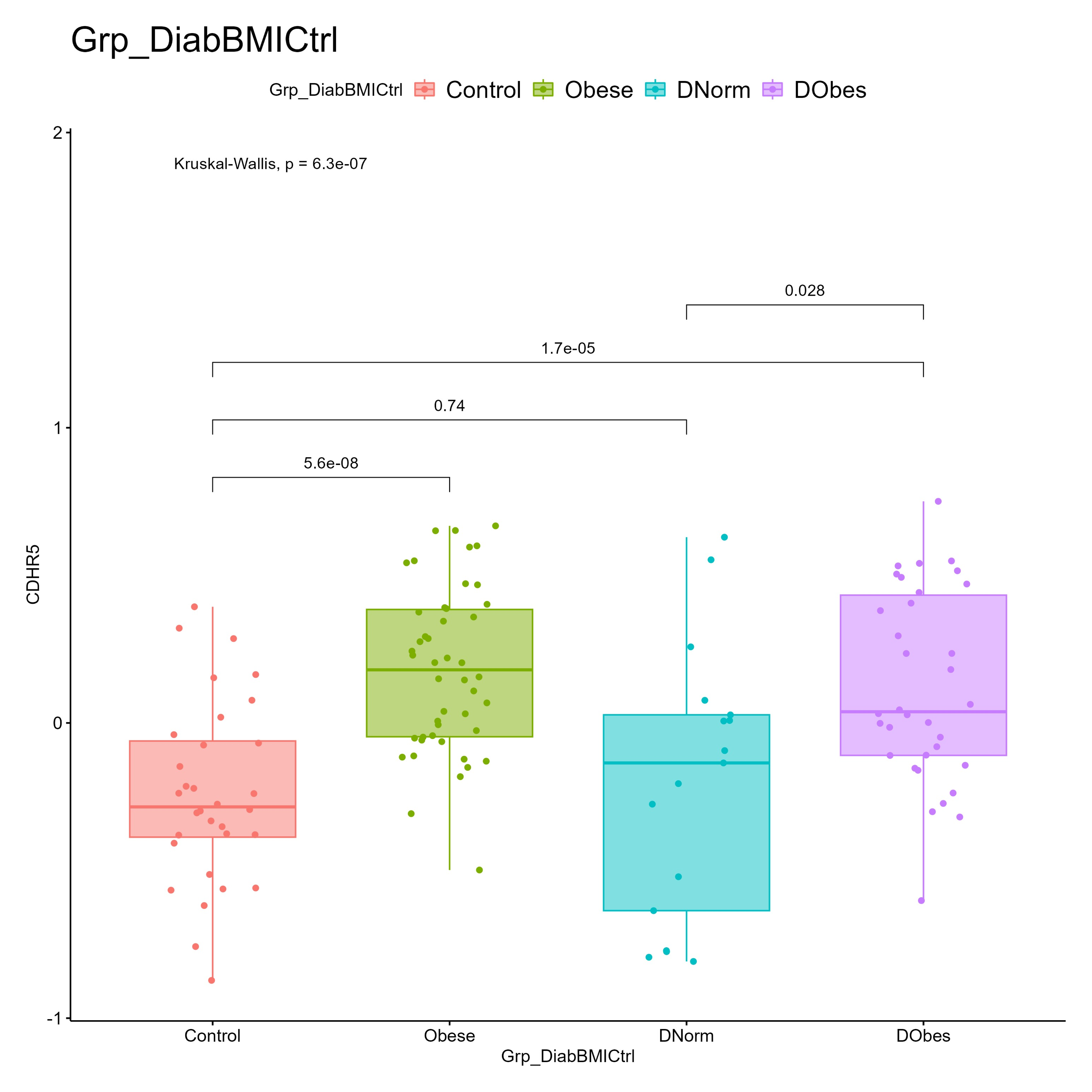

Grp_DiabBMICtrl
Grp_DiabBMICtrl
Control
Obese
DNorm
DObes
Kruskal-Wallis, p = 6.3e-07
0.028
1.7e-05
0.74
5.6e-08
CDHR5
2
1
0
-1
Control
Obese
DNorm
DObes
Grp_DiabBMICtrl

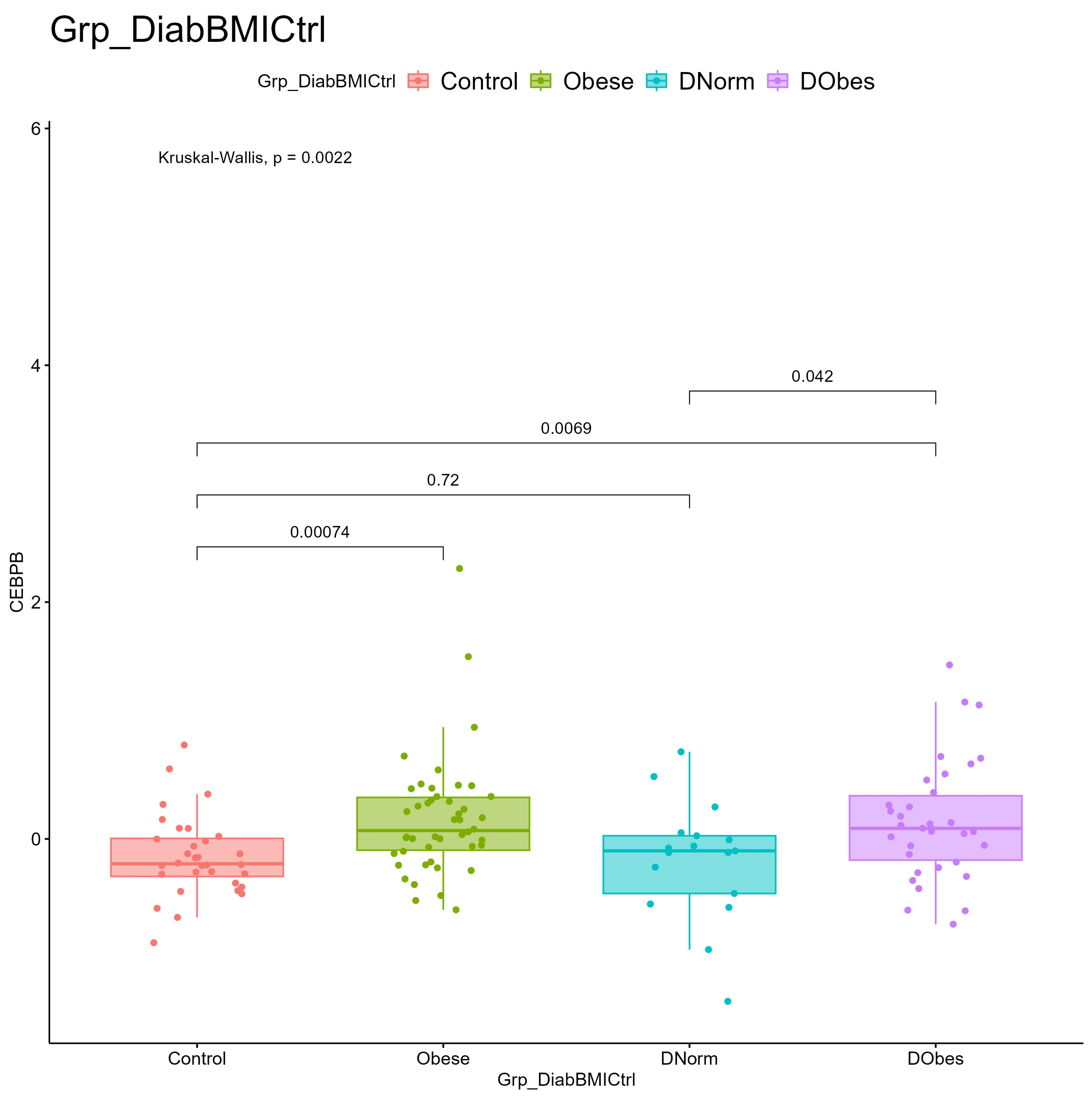

Grp_DiabBMICtrl
Grp_DiabBMICtrl
Control
Obese
DNorm
DObes
Kruskal-Wallis, p = 0.0022
0.042
0.0069
0.72
0.00074
CEBPB
6
4
2
0
Control
Obese
DNorm
DObes
Grp_DiabBMICtrl

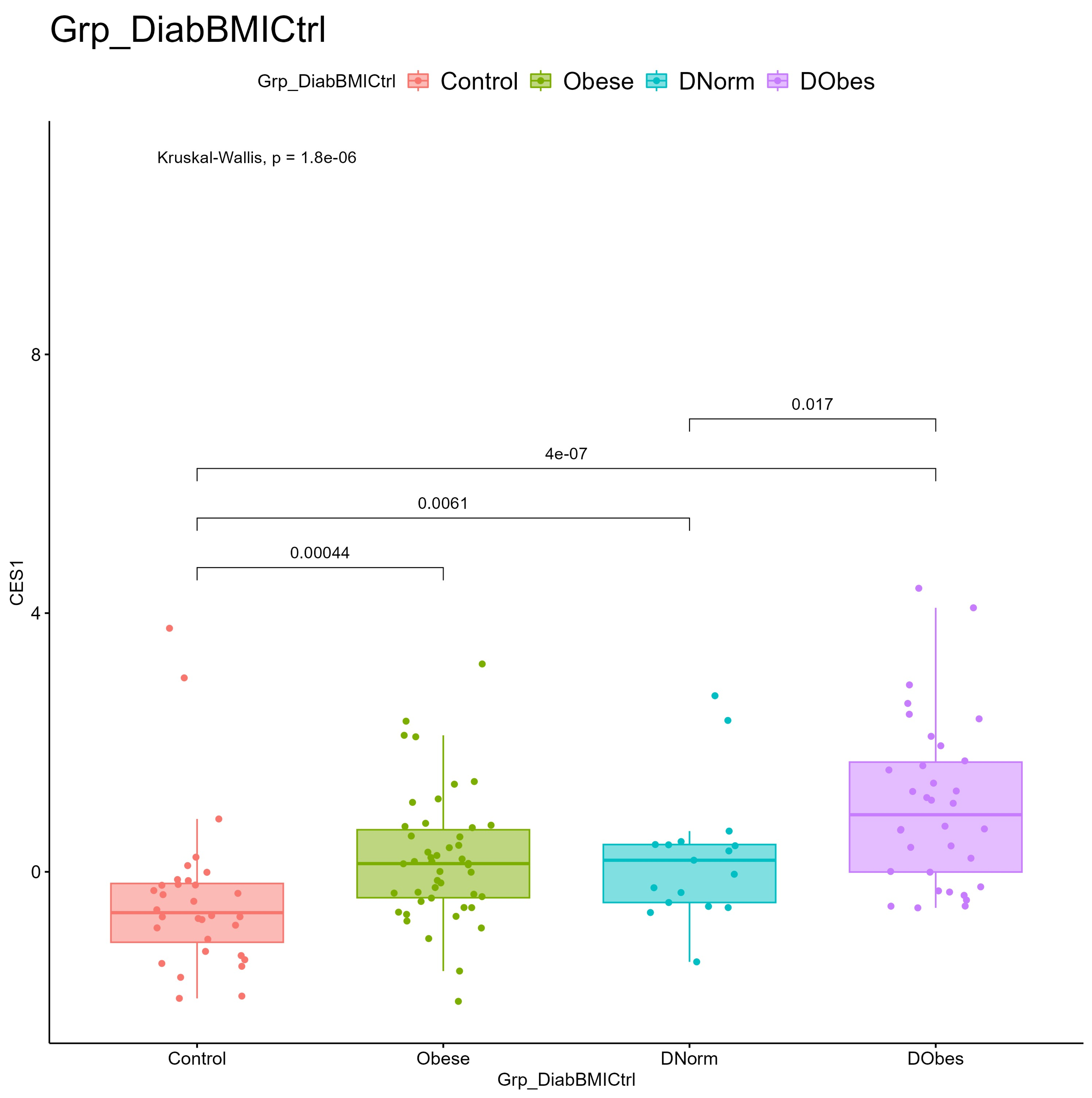
Grp_DiabBMICtrl
Grp_DiabBMICtrl
Control
Obese
DNorm
DObes
Kruskal-Wallis, p = 1.8e-06
0.017
4e-07
0.0061
0.00044
CES1
8
4
0
Control
Obese
DNorm
DObes
Grp_DiabBMICtrl

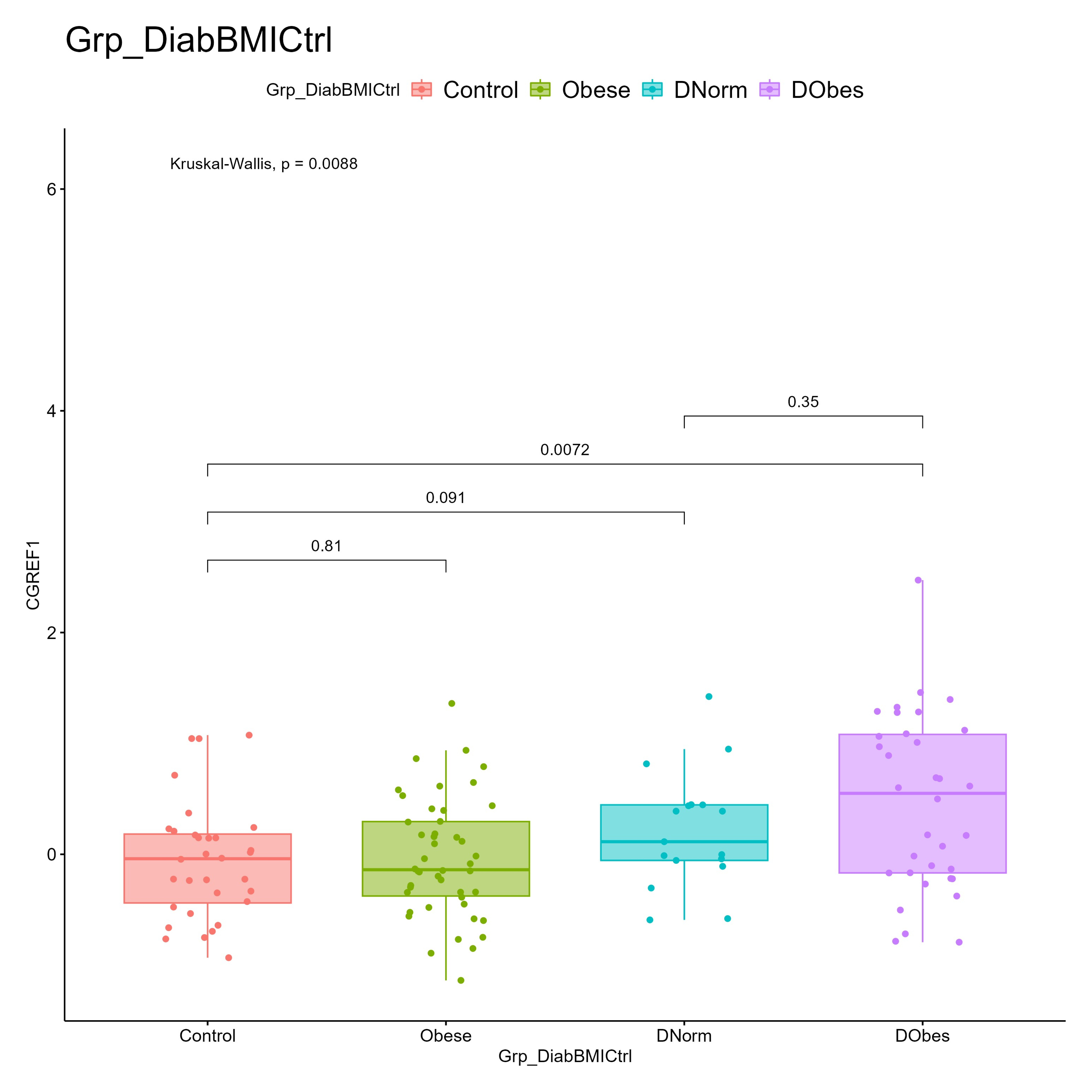
Grp_DiabBMICtrl
Grp_DiabBMICtrl
Control
Obese
DNorm
DObes
Kruskal-Wallis, p = 0.0088
0.35
0.0072
0.091
0.81
CGREF1
6
4
2
0
Control
Obese
DNorm
DObes
Grp_DiabBMICtrl

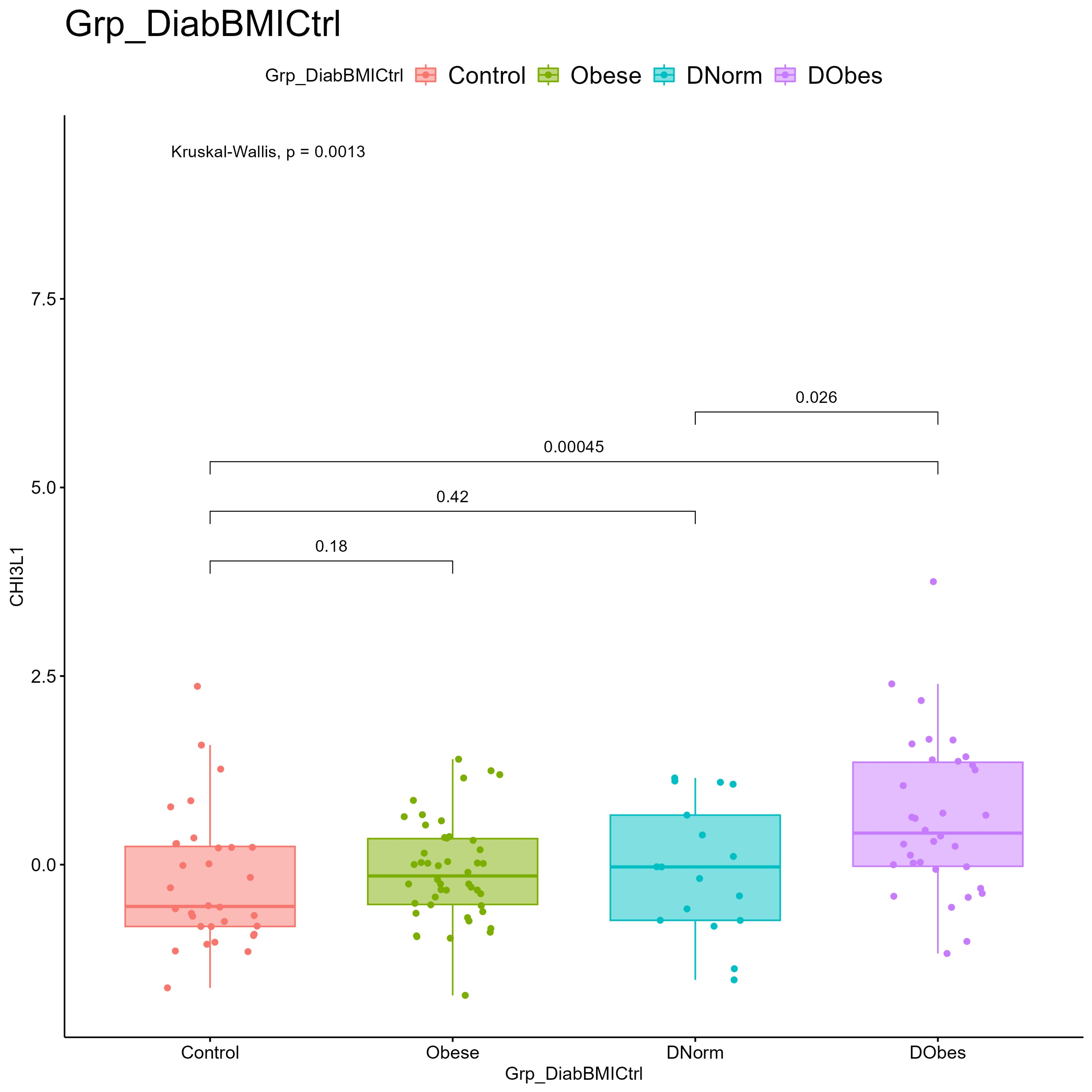
Grp_DiabBMICtrl
Grp_DiabBMICtrl
Control
Obese
DNorm
DObes
Kruskal-Wallis, p = 0.0013
0.026
0.00045
0.42
0.18
CHI3L1
7.5
5.0
2.5
0.0
Control
Obese
DNorm
DObes
Grp_DiabBMICtrl

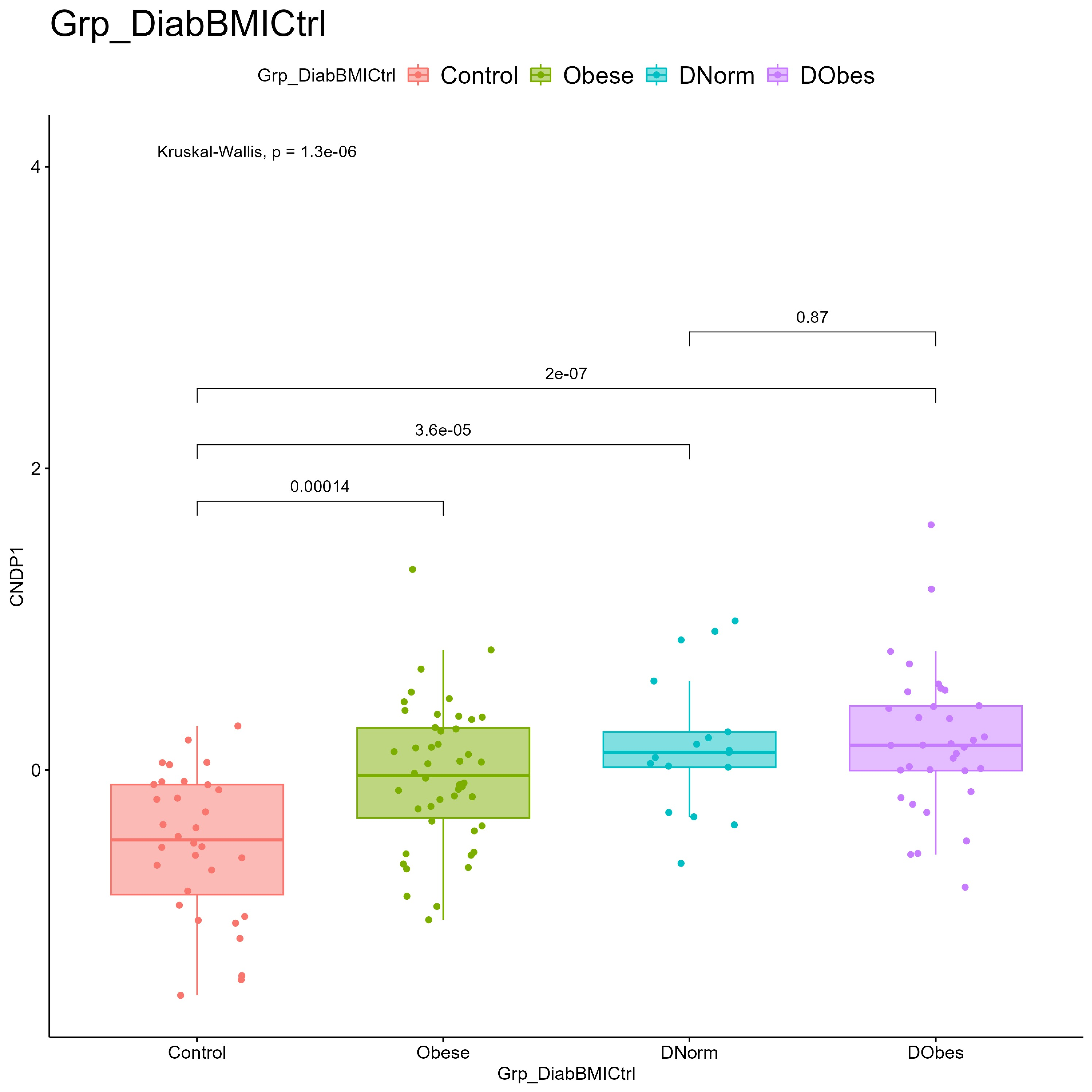

Grp_DiabBMICtrl
Grp_DiabBMICtrl
Control
Obese
DNorm
DObes
Kruskal-Wallis, p = 1.3e-06
0.87
2e-07
3.6e-05
0.00014
4
2
0
CNDP1
Control
Obese
DNorm
DObes
Grp_DiabBMICtrl

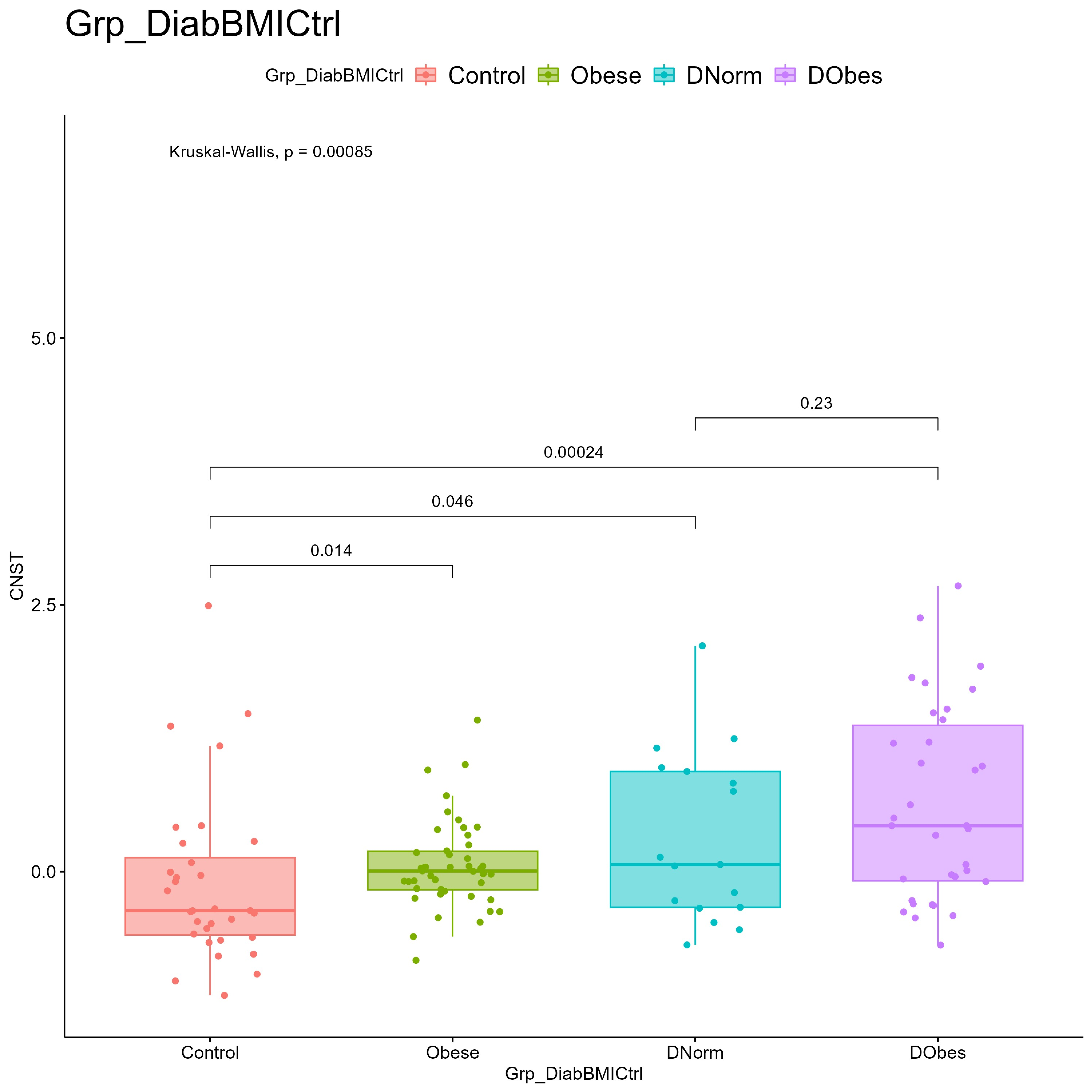
Grp_DiabBMICtrl
Grp_DiabBMICtrl Control Obese DNorm DObes
Kruskal-Wallis, p = 0.00085
0.23
0.00024
0.046
0.014
CNST
5.0
2.5
0.0
Control
Obese
DNorm
DObes
Grp_DiabBMICtrl

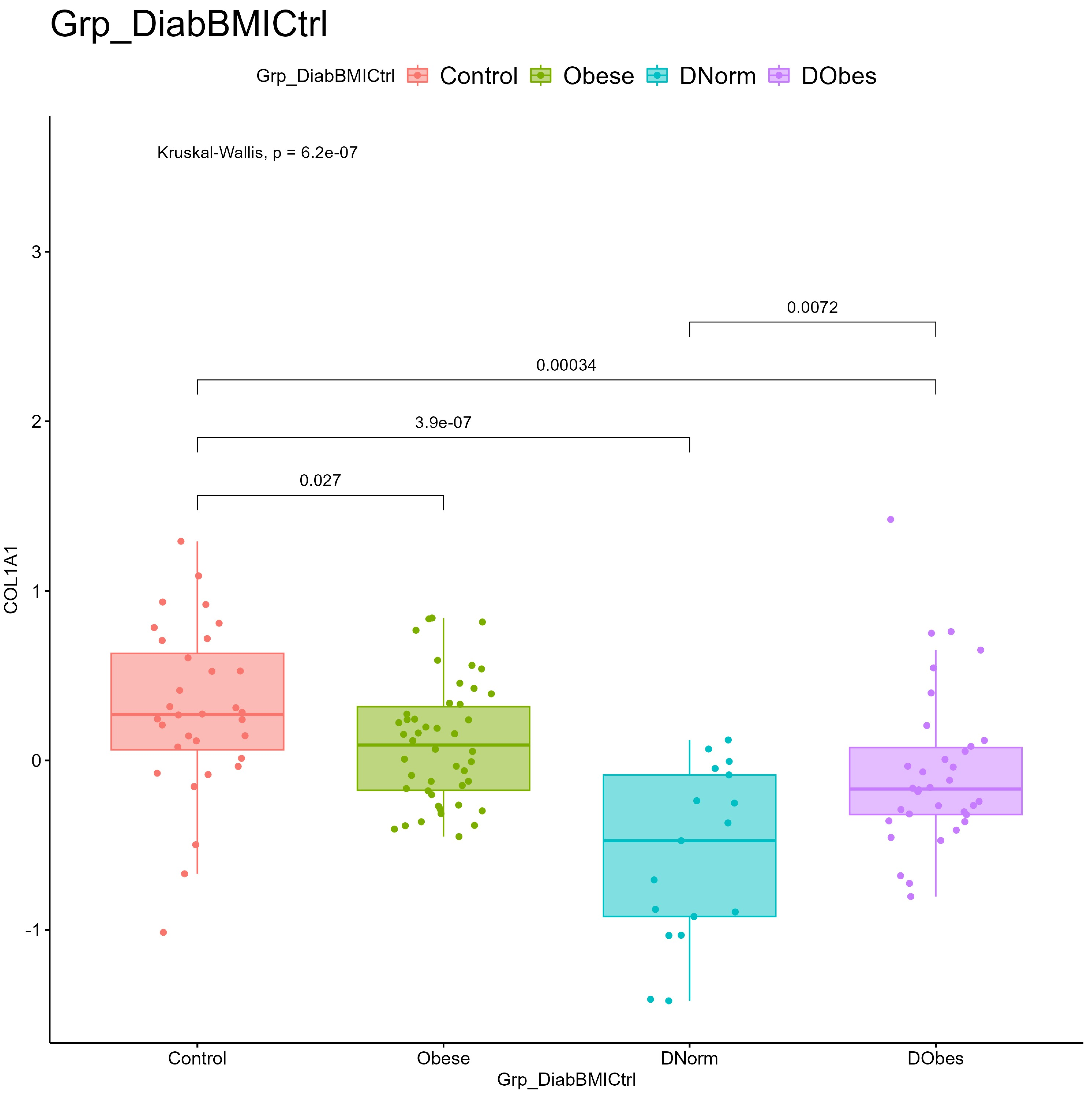

Grp_DiabBMICtrl
Grp_DiabBMICtrl
Control
Obese
DNorm
DObes
Kruskal-Wallis, p = 6.2e-07
0.0072
0.00034
3.9e-07
0.027
COL1A1
3
2
1
0
-1
Control
Obese
DNorm
DObes
Grp_DiabBMICtrl

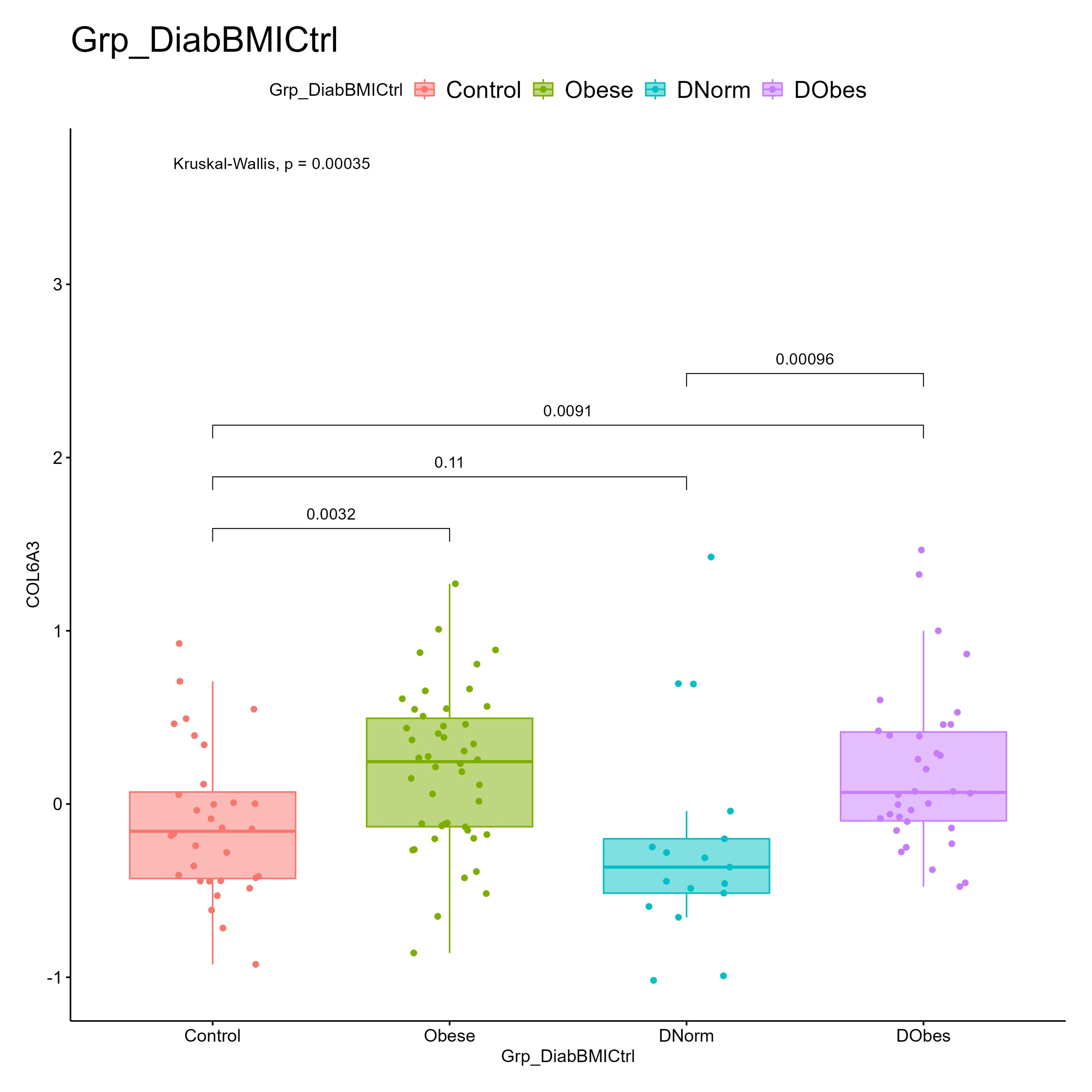

Grp_DiabBMICtrl
Grp_DiabBMICtrl
Control
Obese
DNorm
DObes
Kruskal-Wallis, p = 0.00035
0.00096
0.0091
0.11
0.0032
COL6A3
3
2
1
0
-1
Control
Obese
DNorm
DObes
Grp_DiabBMICtrl

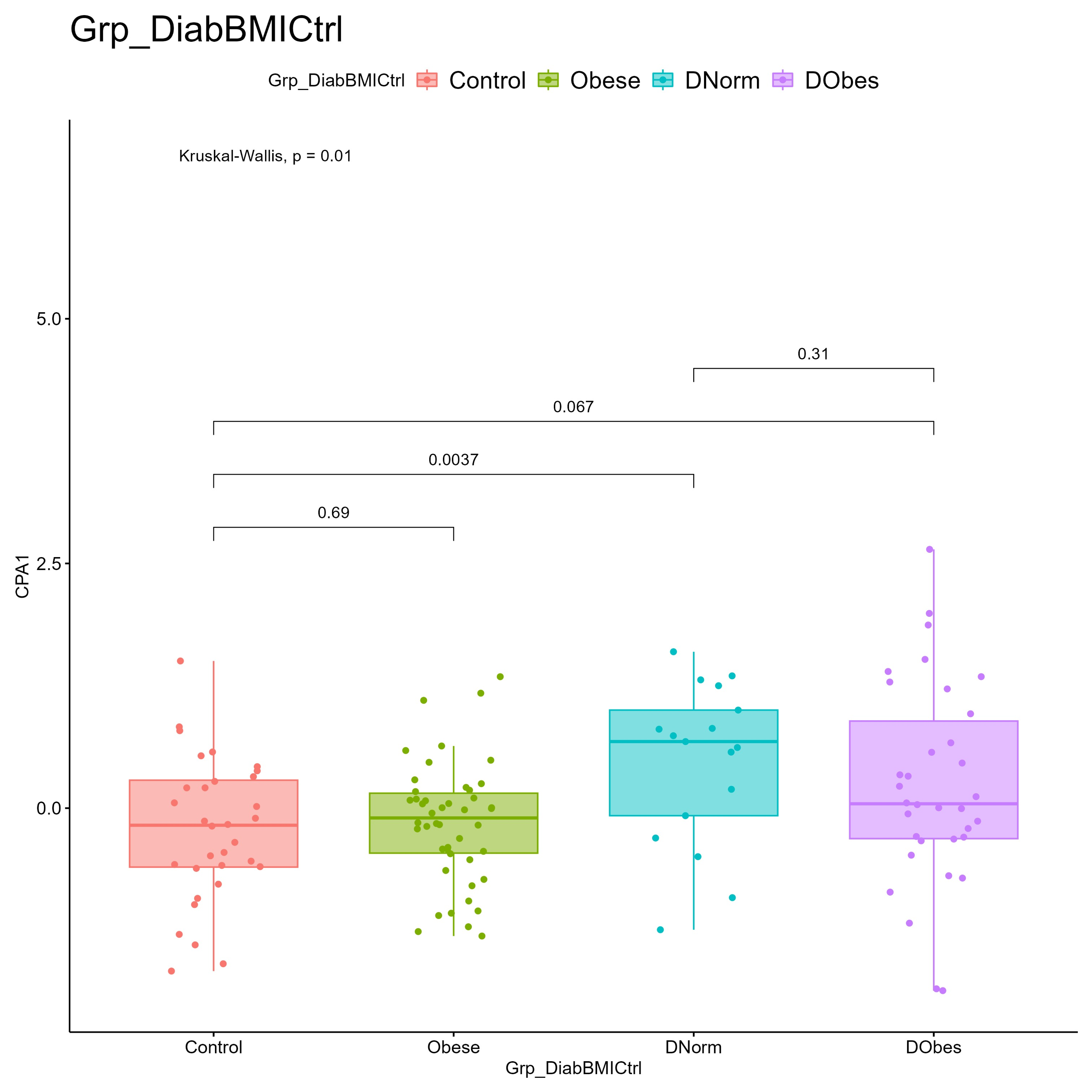
Grp_DiabBMICtrl
Grp_DiabBMICtrl
Control
Obese
DNorm
DObes
Kruskal-Wallis, p = 0.01
0.31
0.067
0.0037
0.69
5.0
2.5
0.0
CPA1
Control
Obese
DNorm
DObes
Grp_DiabBMICtrl

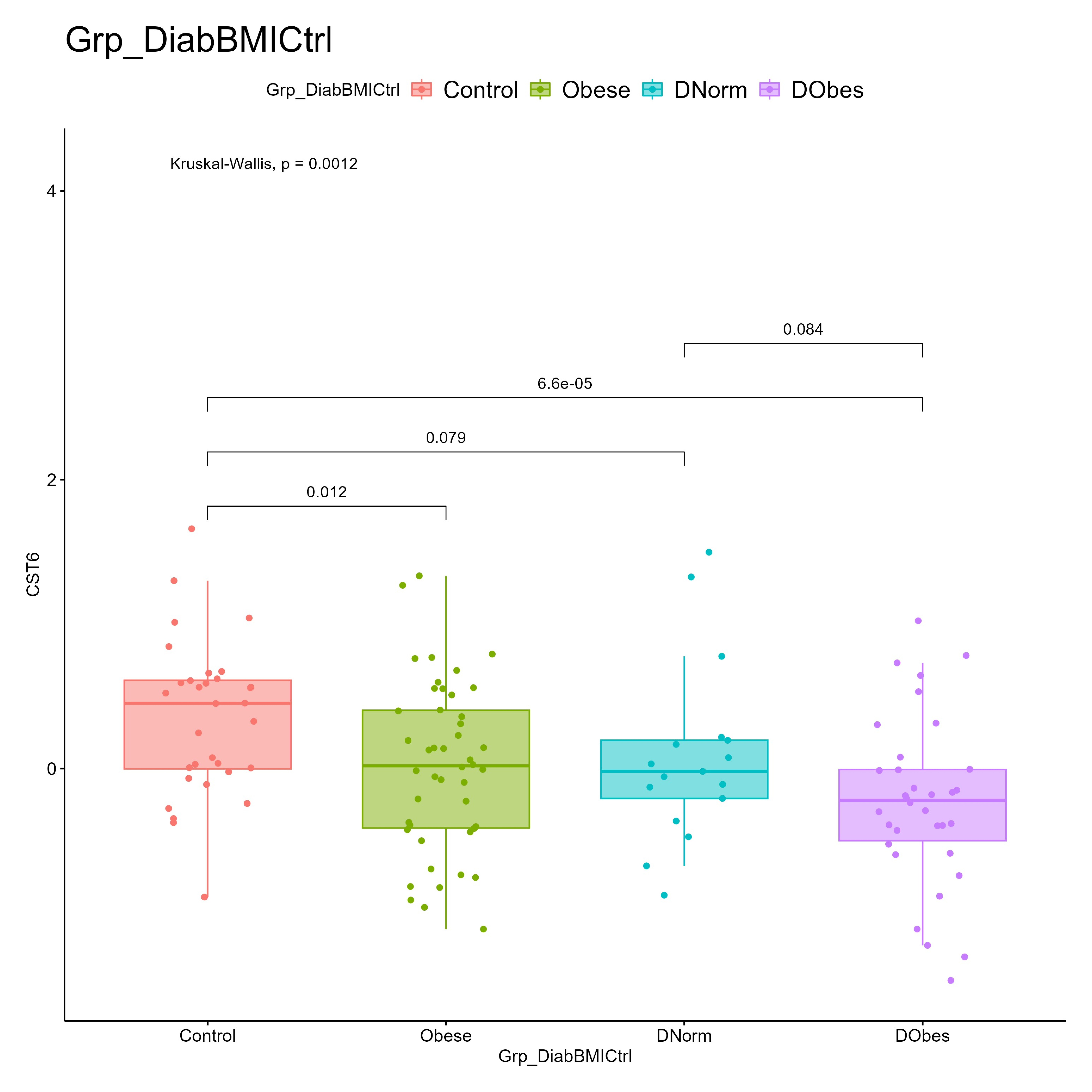

Grp_DiabBMICtrl
Grp_DiabBMICtrl
Control
Obese
DNorm
DObes
Kruskal-Wallis, p = 0.0012
0.084
6.6e-05
0.079
0.012
4
2
0
CST6
Control
Obese
DNorm
DObes
Grp_DiabBMICtrl

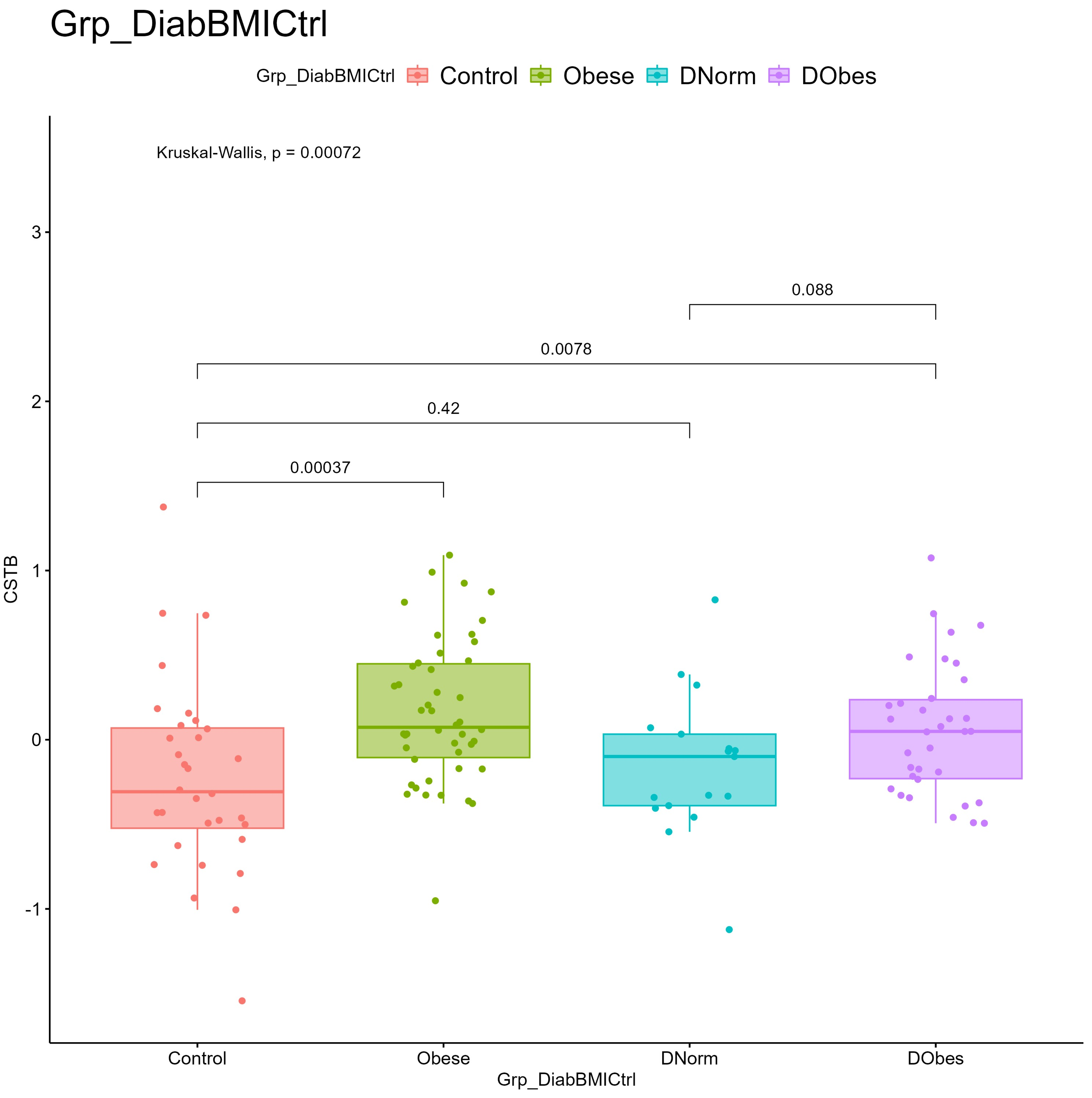
Grp_DiabBMICtrl
Grp_DiabBMICtrl
Control
Obese
DNorm
DObes
Kruskal-Wallis, p = 0.00072
0.088
0.0078
0.42
0.00037
CSTB
3
2
1
0
-1
Control
Obese
DNorm
DObes
Grp_DiabBMICtrl

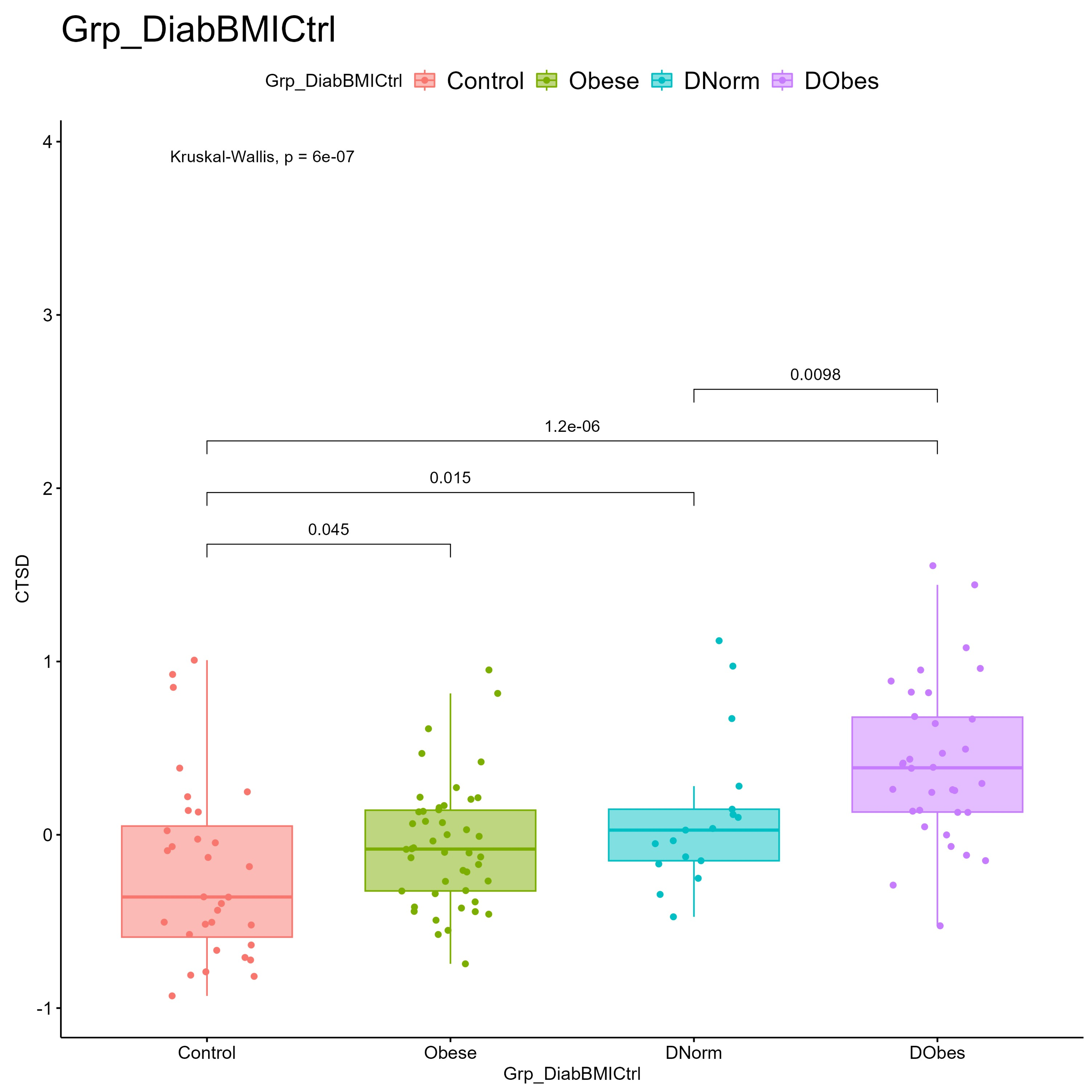
Grp_DiabBMICtrl
Grp_DiabBMICtrl
Control
Obese
DNorm
DObes
Kruskal-Wallis, p = 6e-07
0.0098
1.2e-06
0.015
0.045
CTSD
4
3
2
1
0
-1
Control
Obese
DNorm
DObes
Grp_DiabBMICtrl

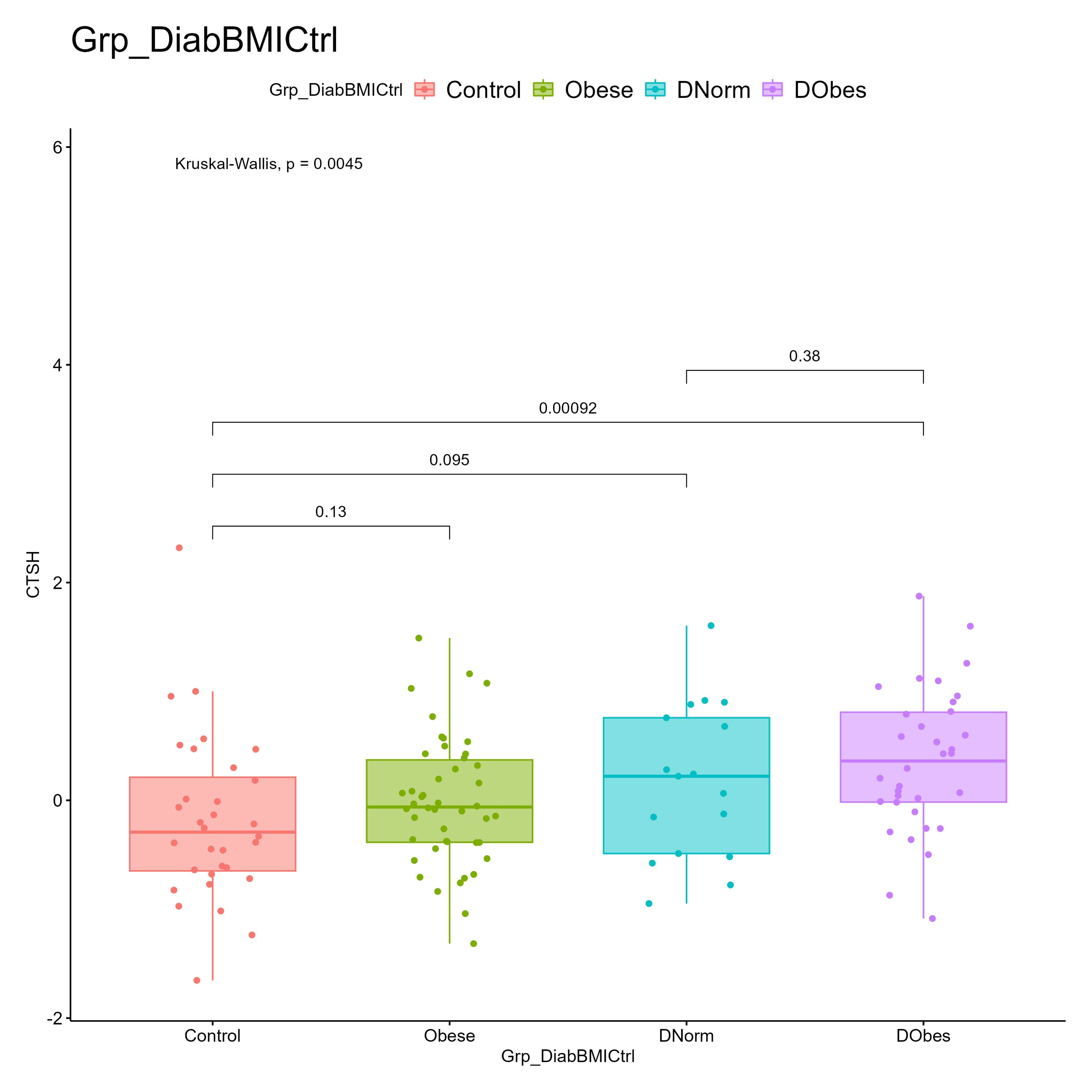
Grp_DiabBMICtrl
Grp_DiabBMICtrl
Control
Obese
DNorm
DObes
Kruskal-Wallis, p = 0.0045
0.38
0.00092
0.095
0.13
CTSH
6
4
2
0
-2
Control
Obese
DNorm
DObes
Grp_DiabBMICtrl

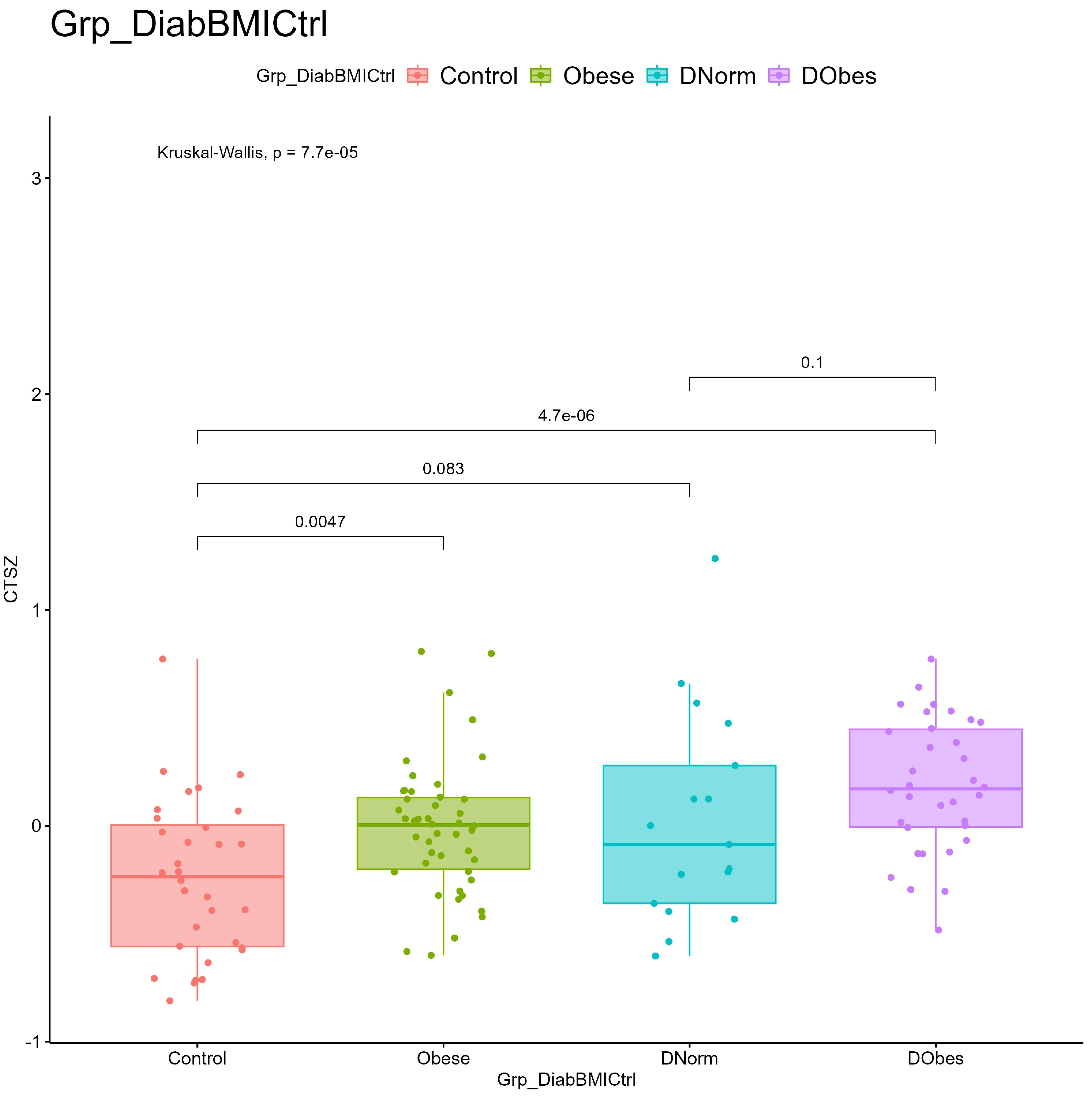
Grp_DiabBMICtrl
Grp_DiabBMICtrl
Control
Obese
DNorm
DObes
Kruskal-Wallis, p = 7.7e-05
0.1
4.7e-06
0.083
0.0047
CTSZ
3
2
1
0
-1
Control
Obese
DNorm
DObes
Grp_DiabBMICtrl

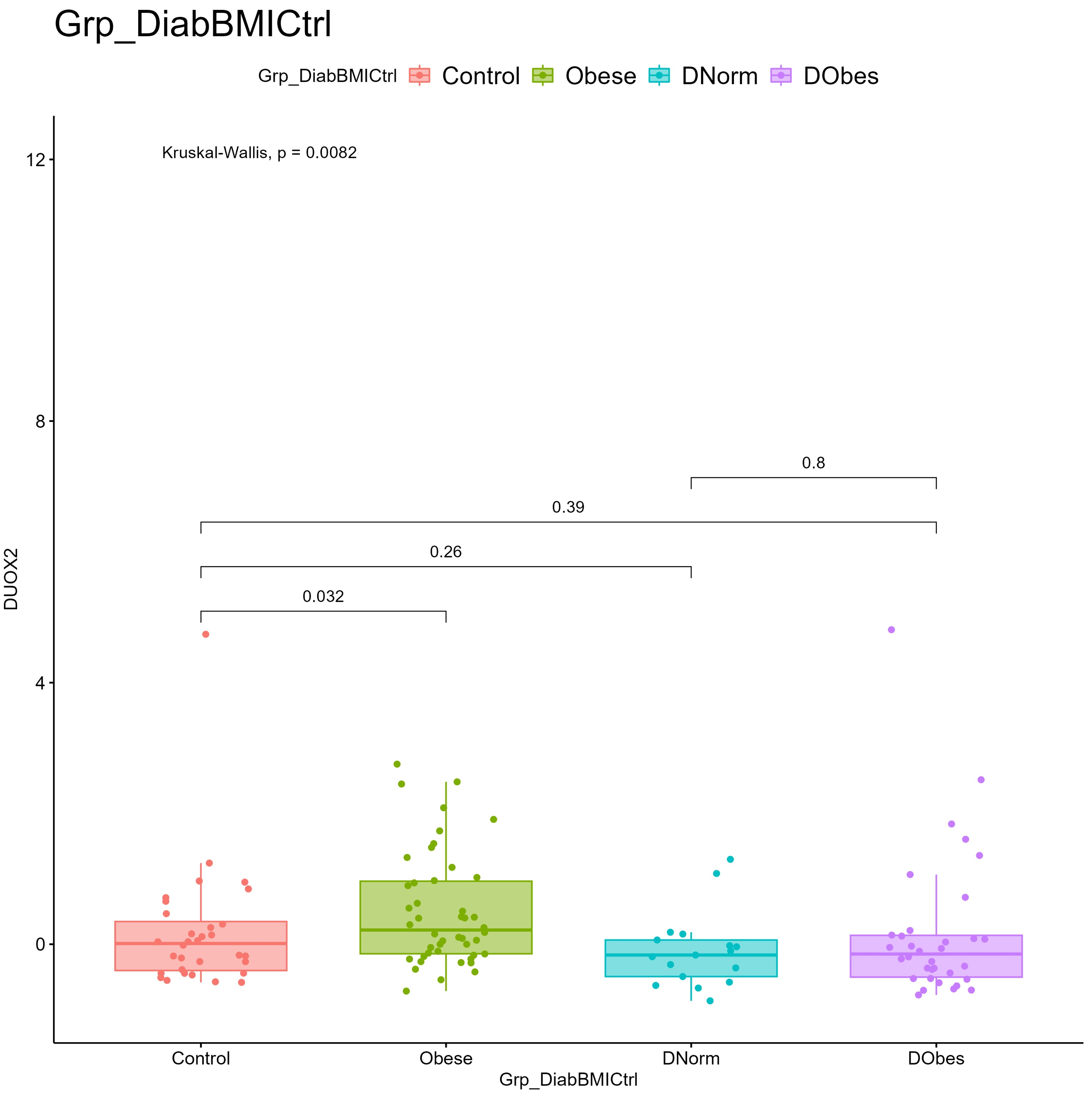

Grp_DiabBMICtrl
Grp_DiabBMICtrl
Control
Obese
DNorm
DObes
Kruskal-Wallis, p = 0.0082
0.8
0.39
0.26
0.032
12
8
4
0
DUOX2
Control
Obese
DNorm
DObes
Grp_DiabBMICtrl

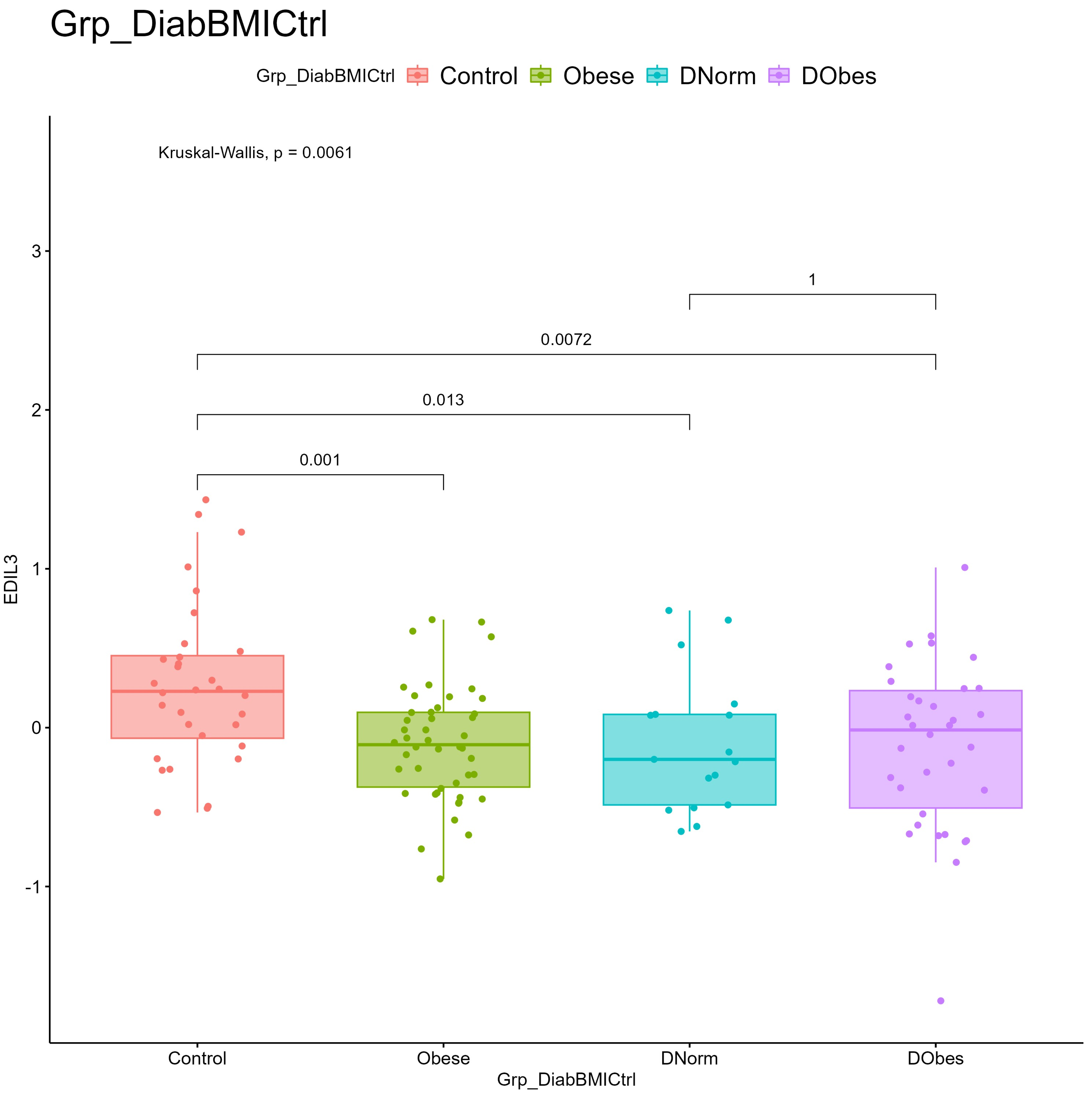

Grp_DiabBMICtrl
Grp_DiabBMICtrl
Control
Obese
DNorm
DObes
Kruskal-Wallis, p = 0.0061
1
0.0072
0.013
0.001
EDIL3
3
2
1
0
-1
Control
Obese
DNorm
DObes
Grp_DiabBMICtrl

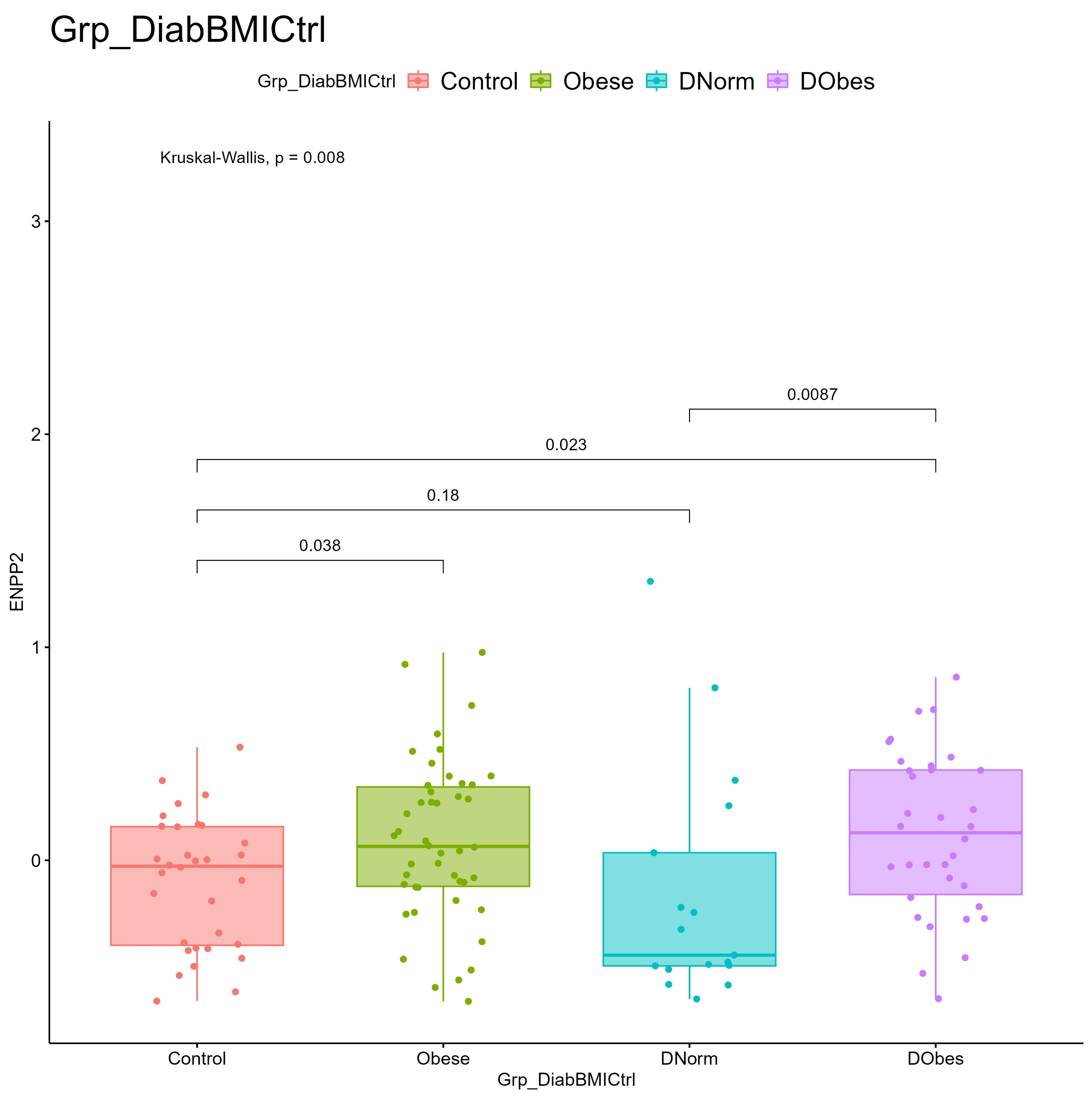

Grp_DiabBMICtrl
Grp_DiabBMICtrl Control Obese DNorm DObes
Kruskal-Wallis, p = 0.008
0.0087
0.023
0.18
0.038
ENPP2
3
2
1
0
Control
Obese
DNorm
DObes
Grp_DiabBMICtrl

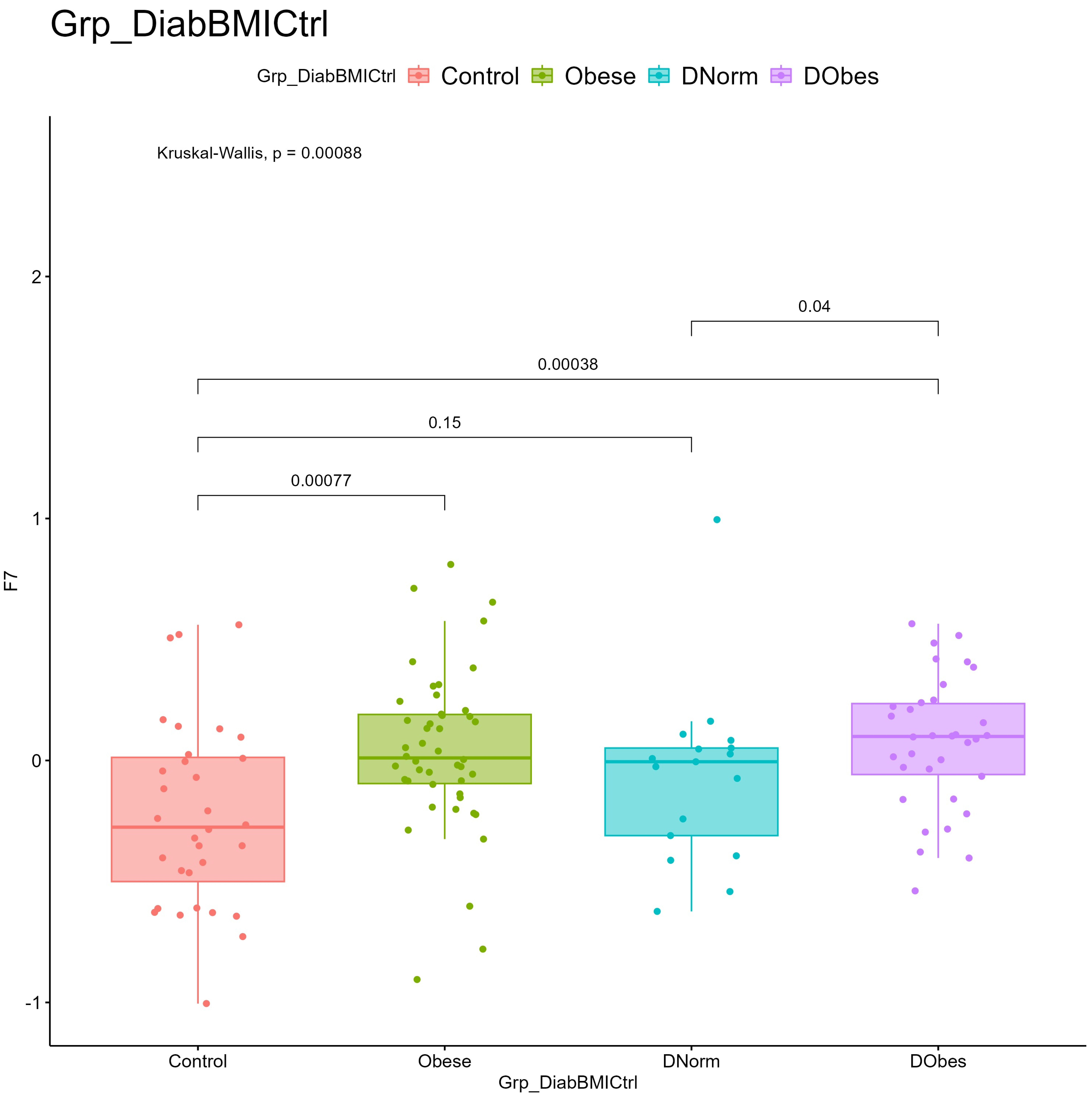
Grp_DiabBMICtrl
Grp_DiabBMICtrl Control Obese DNorm DObes
Kruskal-Wallis, p = 0.00088
0.04
0.00038
0.15
0.00077
F7
2
1
0
-1
Control
Obese
DNorm
DObes
Grp_DiabBMICtrl

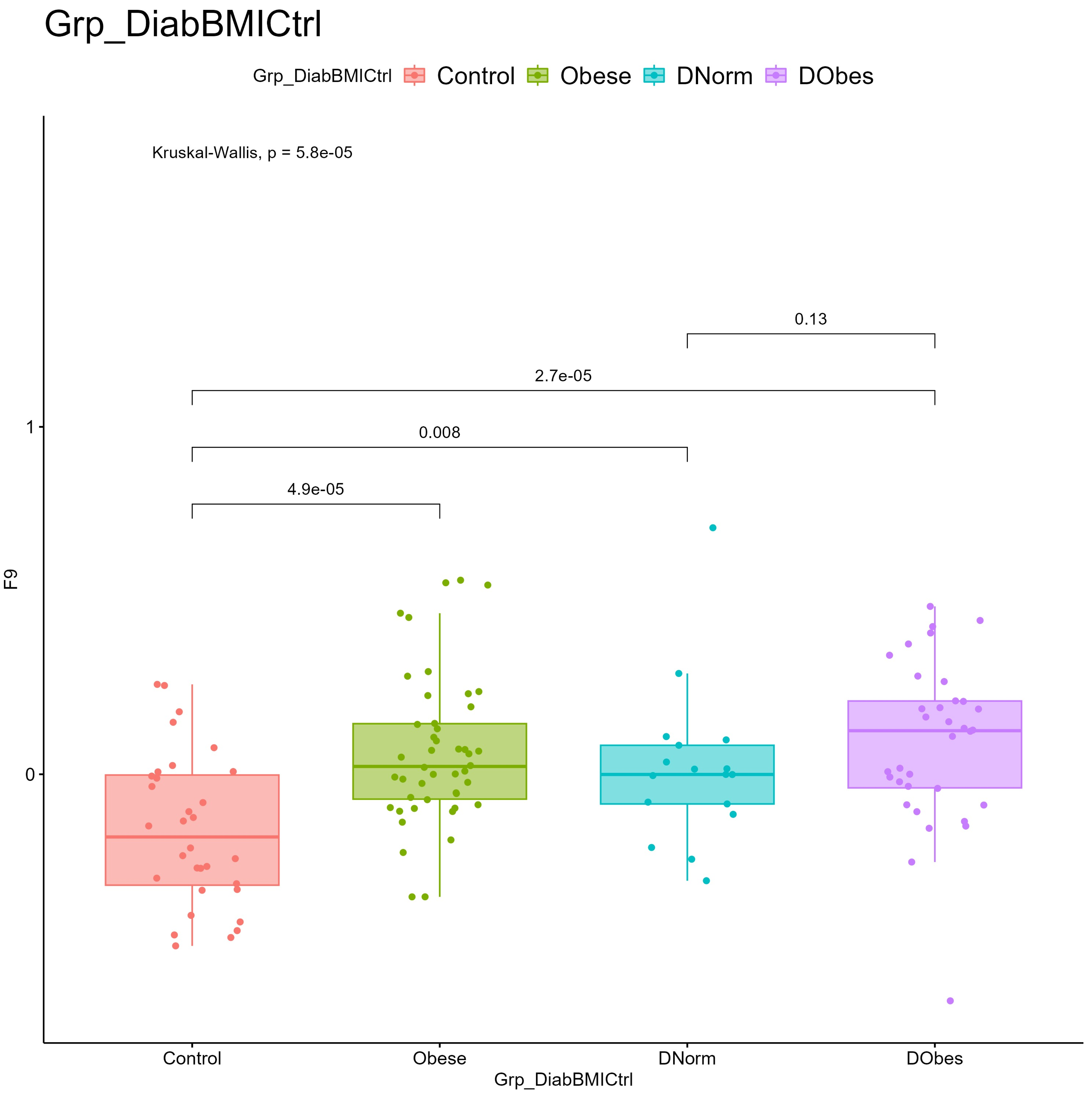

Grp_DiabBMICtrl
Grp_DiabBMICtrl
Control
Obese
DNorm
DObes
Kruskal-Wallis, p = 5.8e-05
0.13
2.7e-05
0.008
4.9e-05
F9
1
0
Control
Obese
DNorm
DObes
Grp_DiabBMICtrl

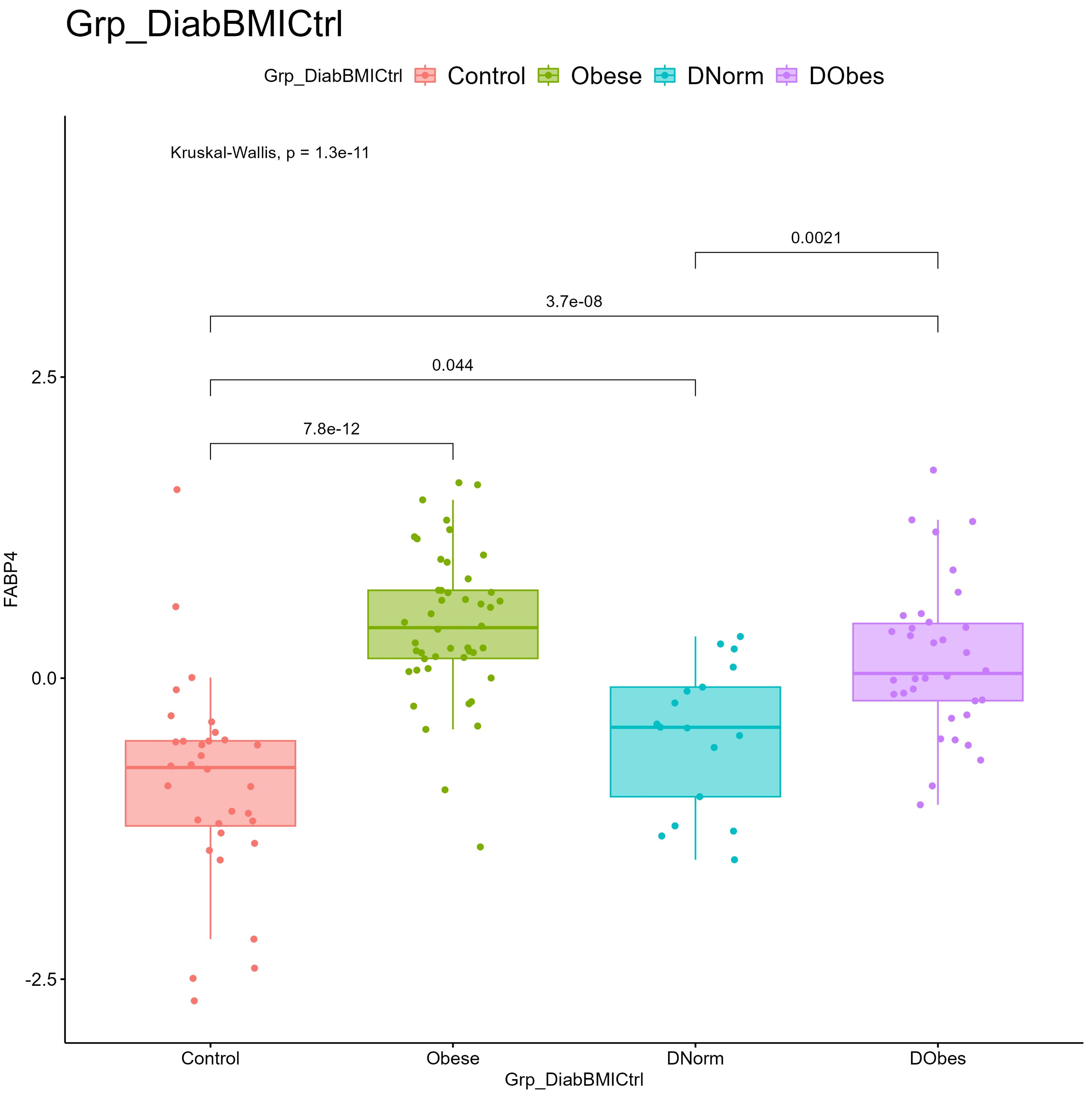

Grp_DiabBMICtrl
Grp_DiabBMICtrl
Control
Obese
DNorm
DObes
Kruskal-Wallis, p = 1.3e-11
0.0021
3.7e-08
0.044
7.8e-12
2.5
0.0
-2.5
FABP4
Control
Obese
DNorm
DObes
Grp_DiabBMICtrl

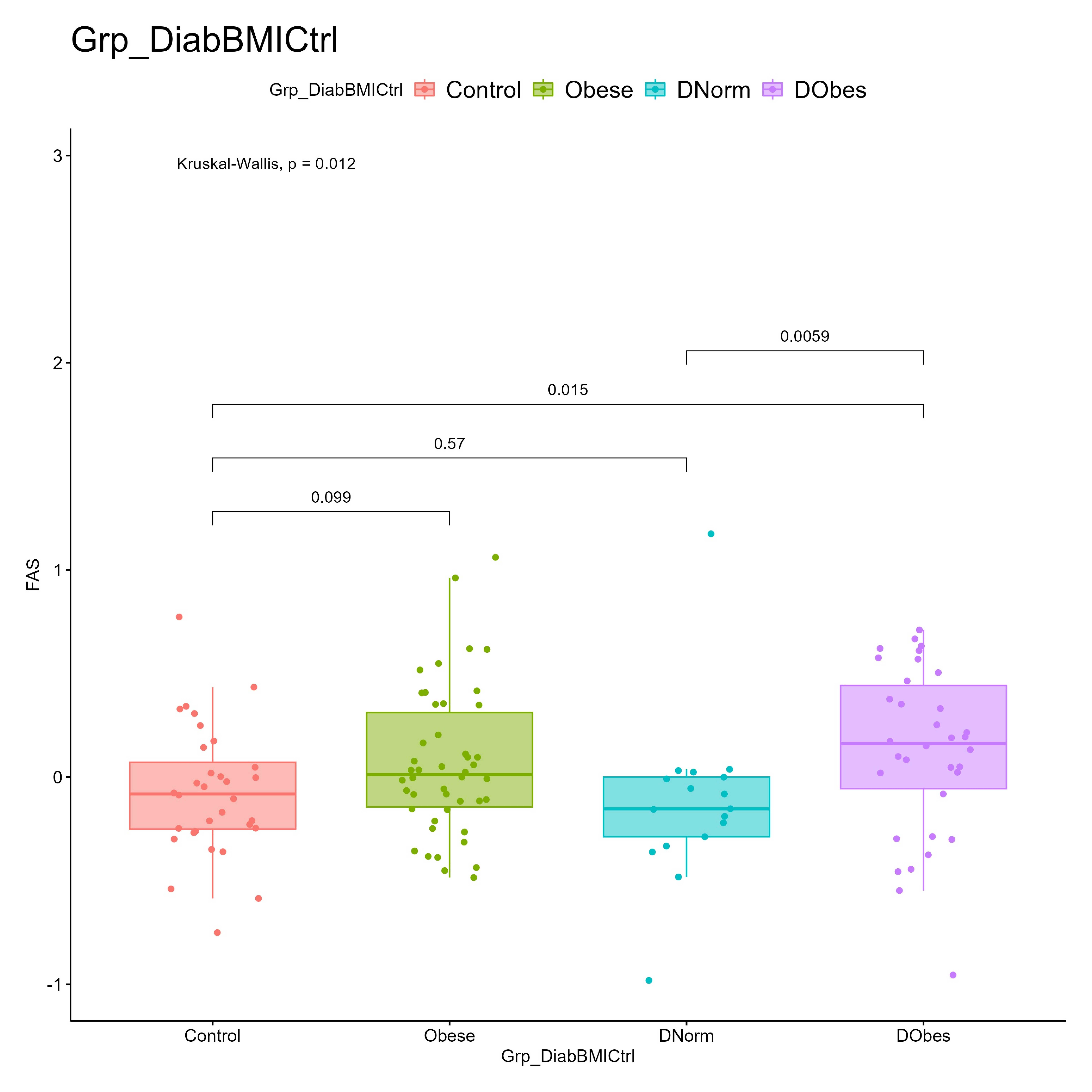

Grp_DiabBMICtrl
Grp_DiabBMICtrl
Control
Obese
DNorm
DObes
Kruskal-Wallis, p = 0.012
0.0059
0.015
0.57
0.099
FAS
3
2
1
0
-1
Control
Obese
DNorm
DObes
Grp_DiabBMICtrl

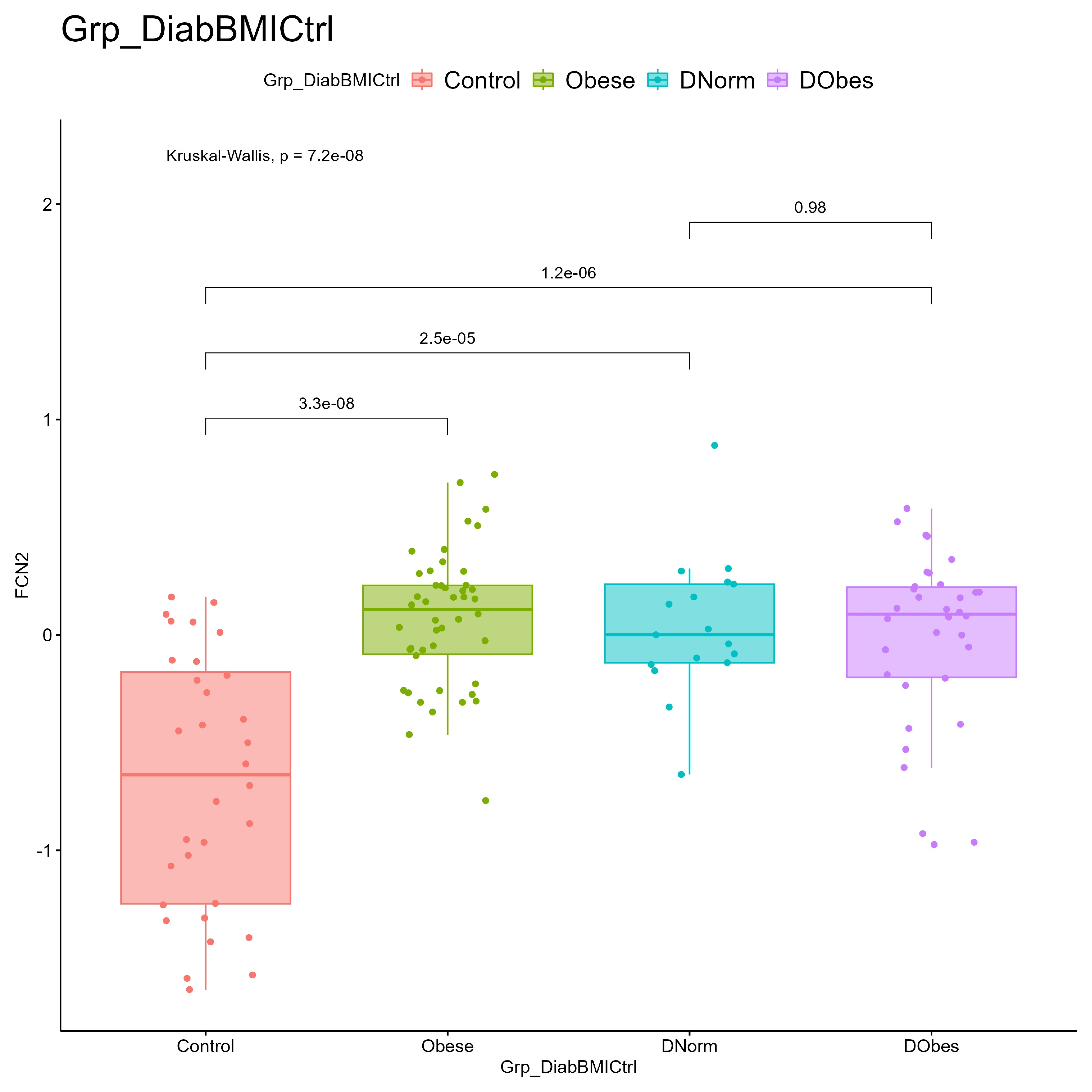

Grp_DiabBMICtrl
Grp_DiabBMICtrl
Control
Obese
DNorm
DObes
Kruskal-Wallis, p = 7.2e-08
0.98
1.2e-06
2.5e-05
3.3e-08
FCN2
2
1
0
-1
Control
Obese
DNorm
DObes
Grp_DiabBMICtrl

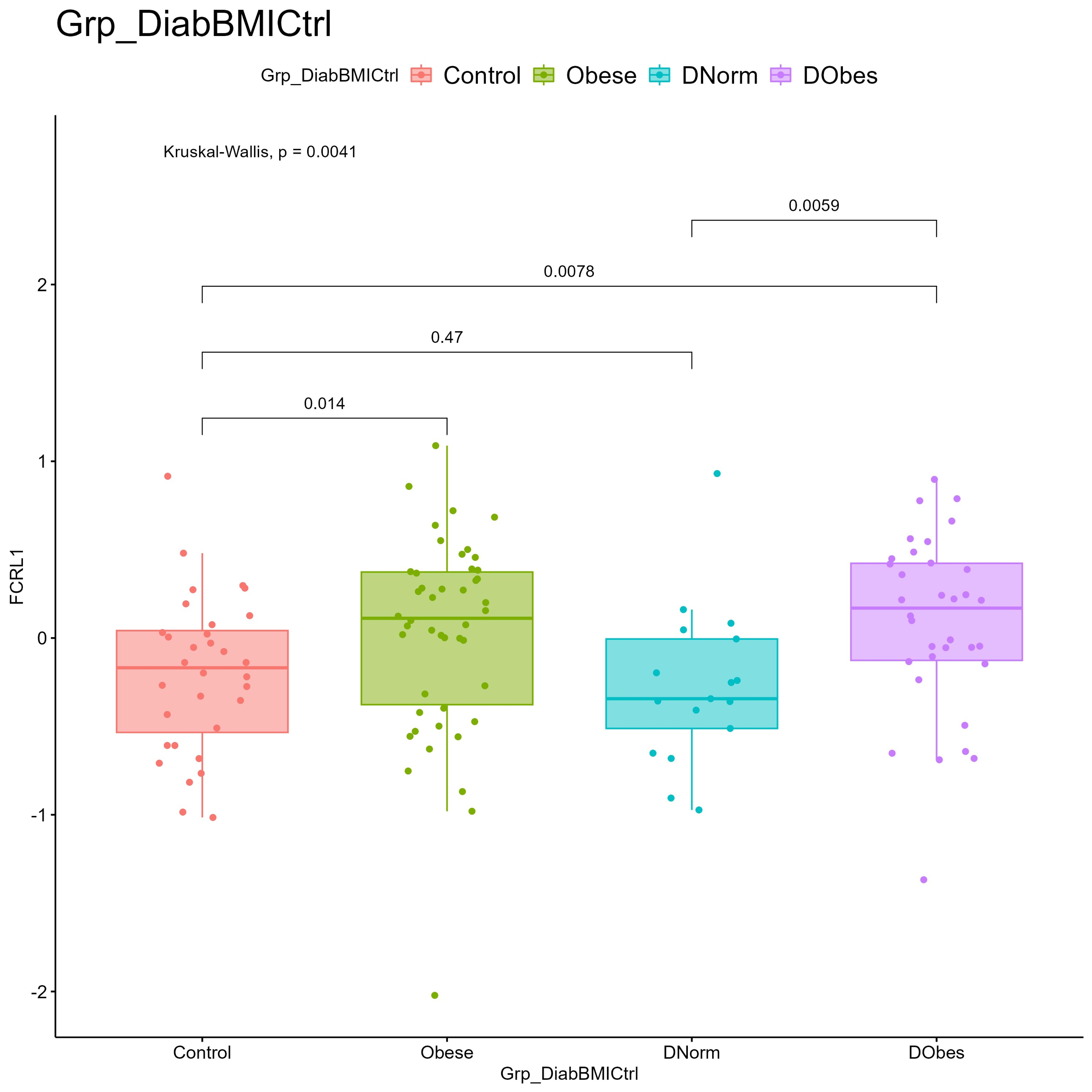

Grp_DiabBMICtrl
Grp_DiabBMICtrl
Control
Obese
DNorm
DObes
Kruskal-Wallis, p = 0.0041
0.0059
0.0078
0.47
0.014
FCRL1
2
1
0
-1
-2
Control
Obese
DNorm
DObes
Grp_DiabBMICtrl

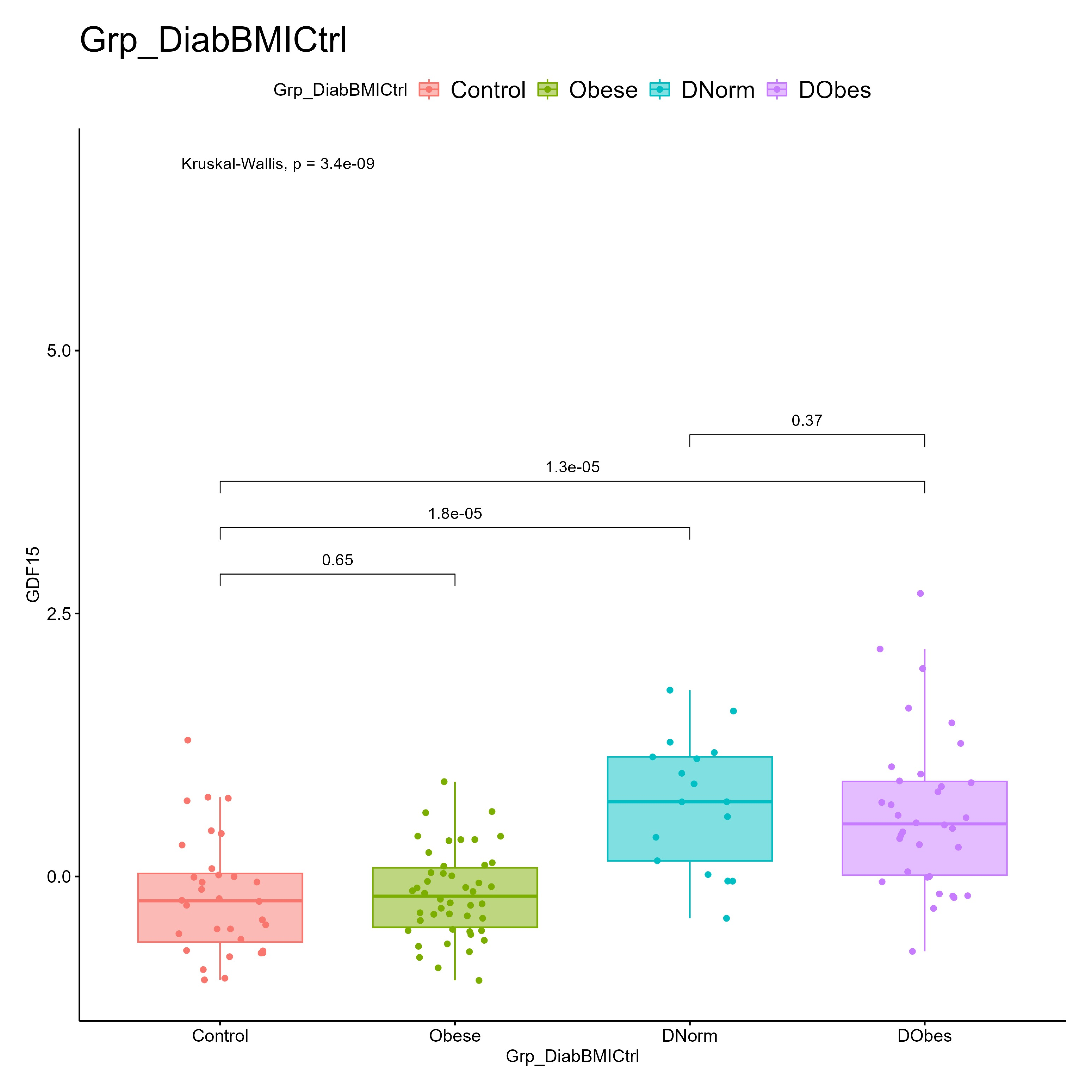
Grp_DiabBMICtrl
Grp_DiabBMICtrl
Control
Obese
DNorm
DObes
Kruskal-Wallis, p = 3.4e-09
0.37
1.3e-05
1.8e-05
0.65
GDF15
5.0
2.5
0.0
Control
Obese
DNorm
DObes
Grp_DiabBMICtrl

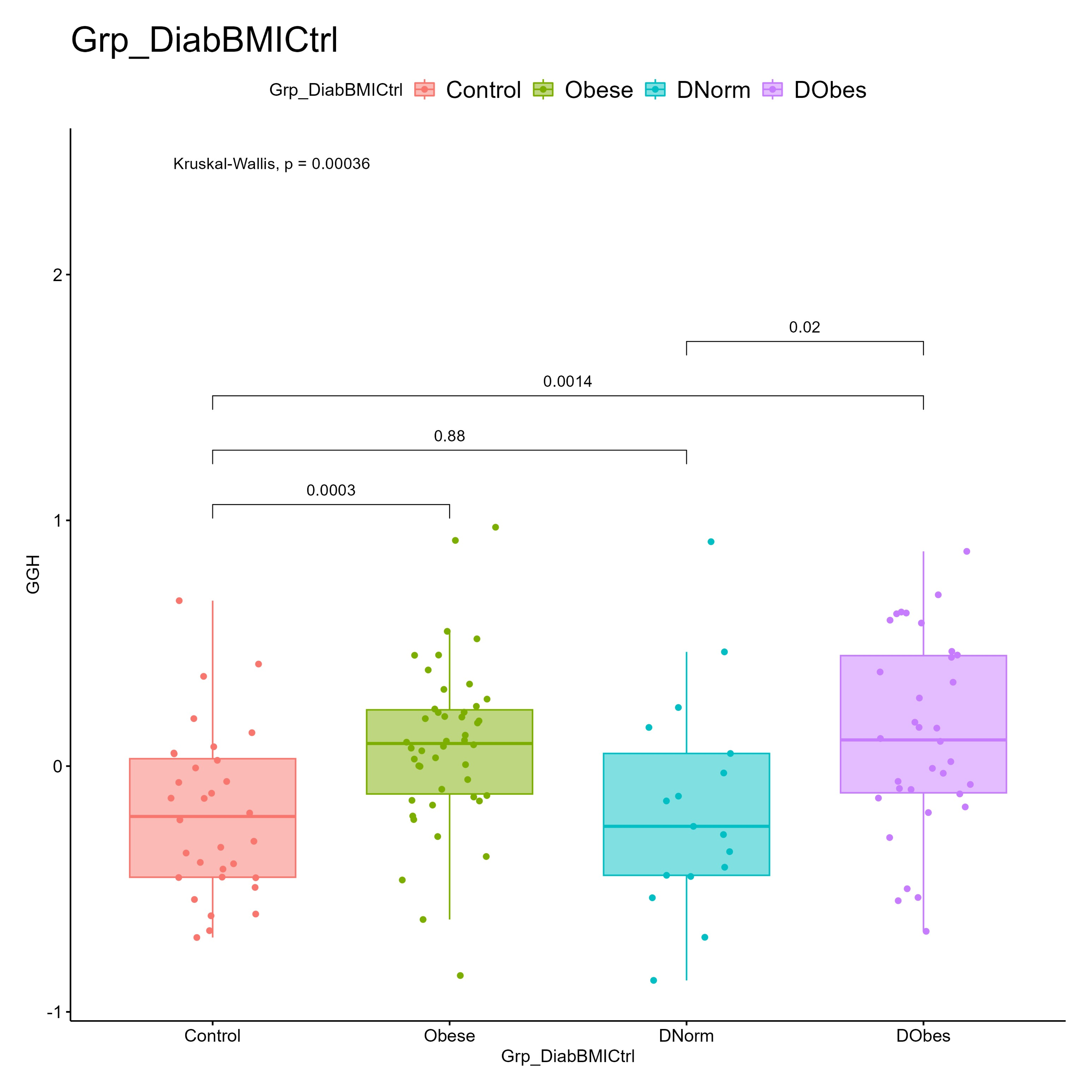
Grp_DiabBMICtrl
Grp_DiabBMICtrl
Control
Obese
DNorm
DObes
Kruskal-Wallis, p = 0.00036
0.02
0.0014
0.88
0.0003
GGH
2
1
0
-1
Control
Obese
DNorm
DObes
Grp_DiabBMICtrl

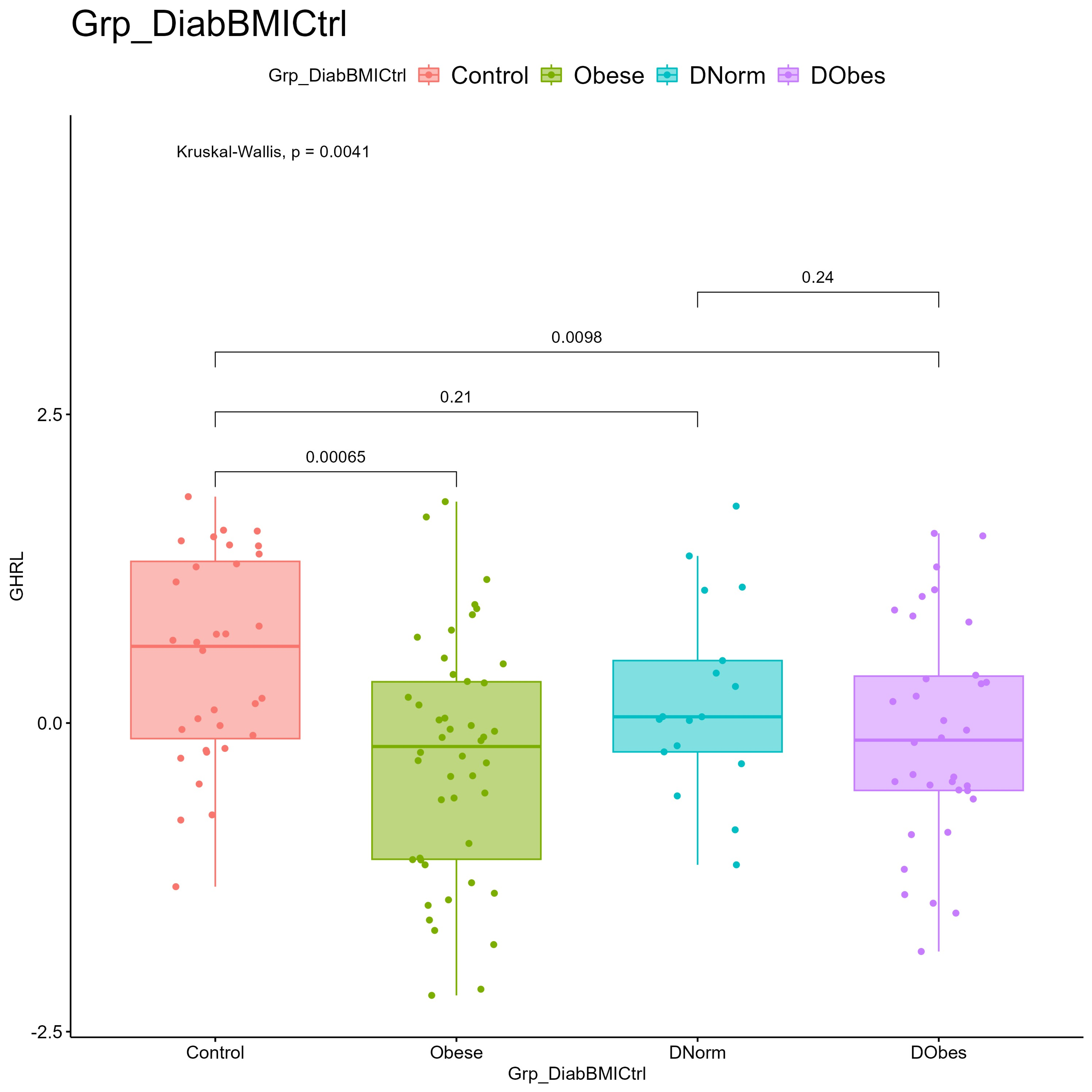

Grp_DiabBMICtrl
Grp_DiabBMICtrl
Control
Obese
DNorm
DObes
Kruskal-Wallis, p = 0.0041
0.24
0.0098
0.21
0.00065
GHRL
2.5
0.0
-2.5
Control
Obese
DNorm
DObes
Grp_DiabBMICtrl

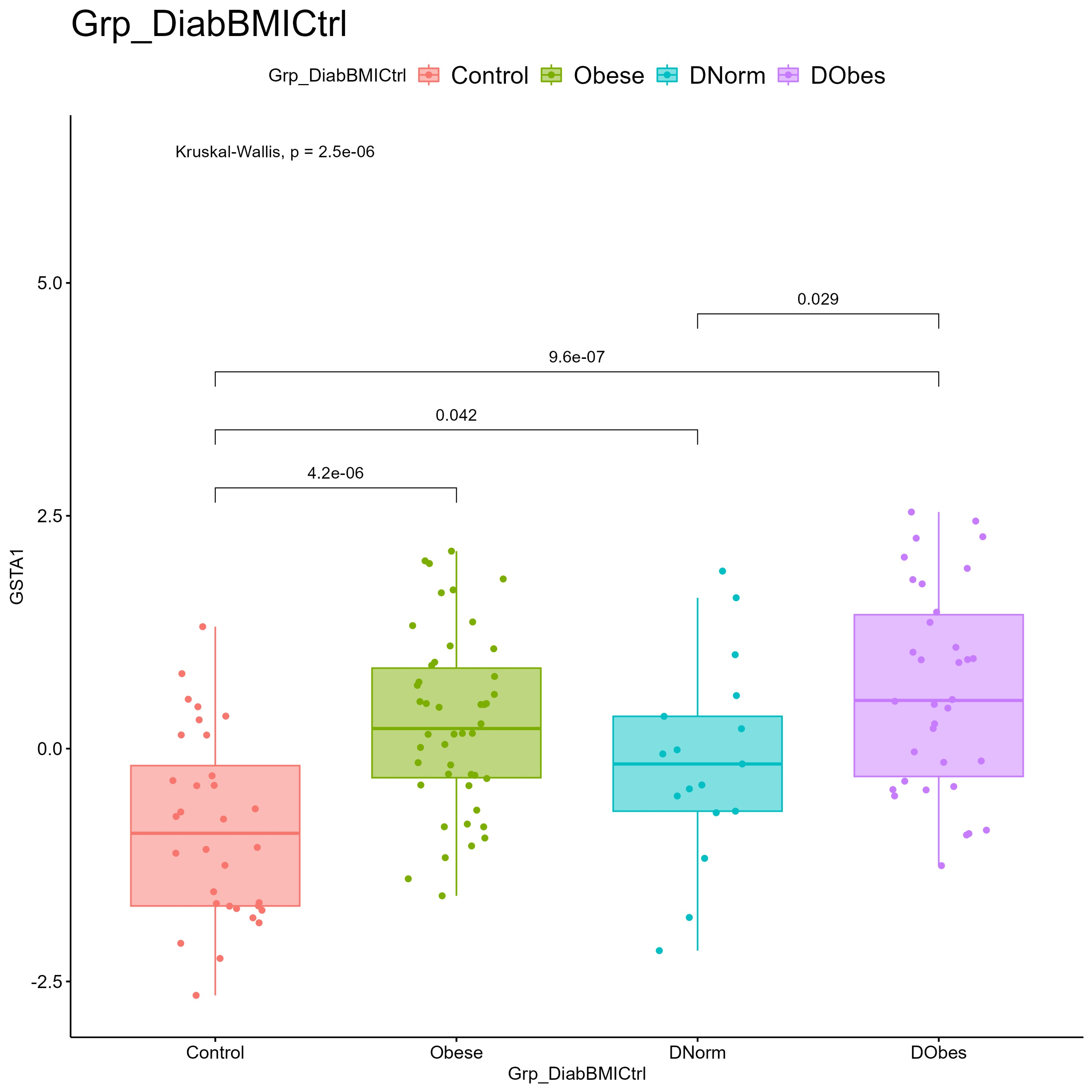

Grp_DiabBMICtrl
Grp_DiabBMICtrl
Control
Obese
DNorm
DObes
Kruskal-Wallis, p = 2.5e-06
0.029
9.6e-07
0.042
4.2e-06
5.0
2.5
0.0
-2.5
GSTA1
Control
Obese
DNorm
DObes
Grp_DiabBMICtrl

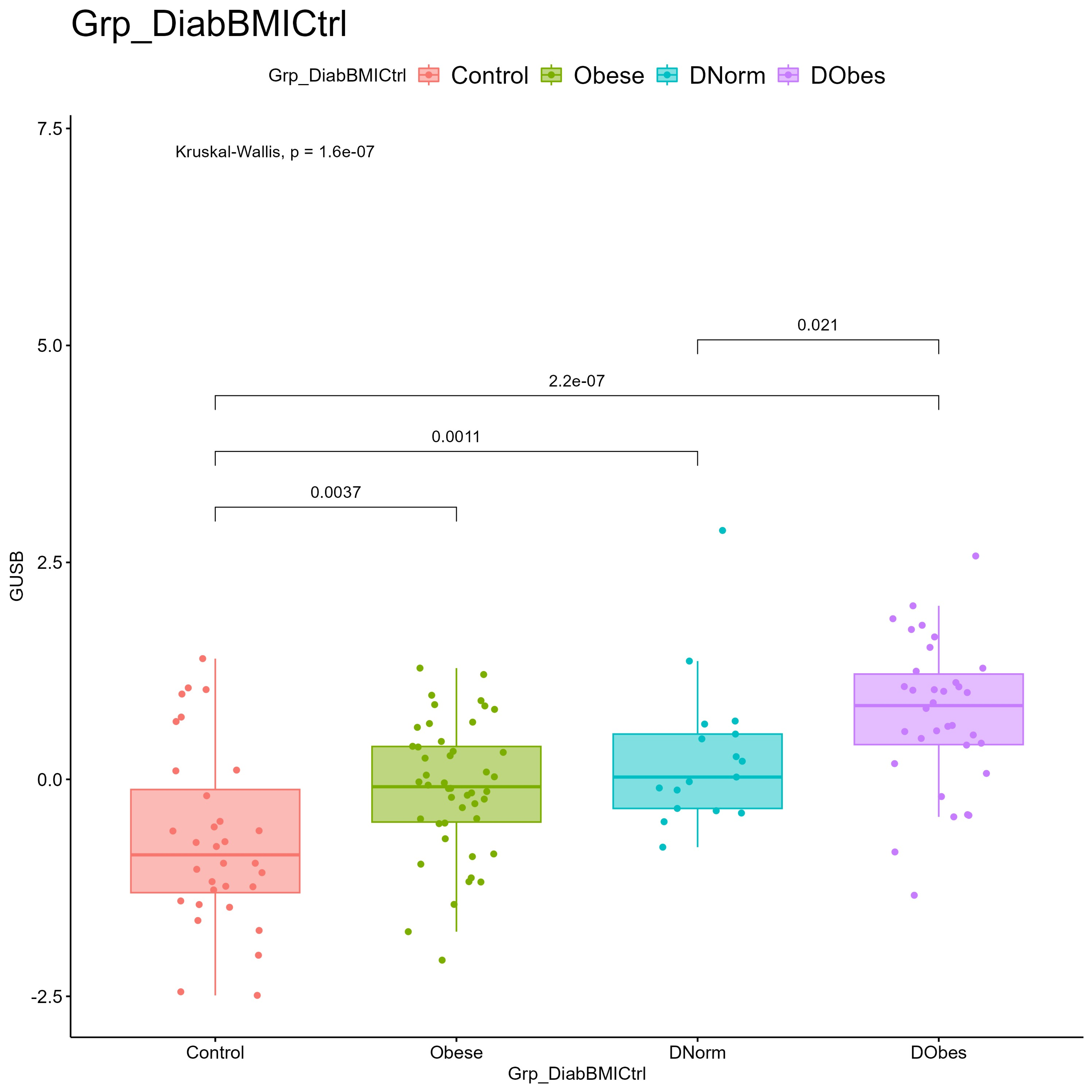
Grp_DiabBMICtrl
Grp_DiabBMICtrl
Control
Obese
DNorm
DObes
Kruskal-Wallis, p = 1.6e-07
0.021
2.2e-07
0.0011
0.0037
7.5
5.0
2.5
0.0
-2.5
GUSB
Control
Obese
DNorm
DObes
Grp_DiabBMICtrl

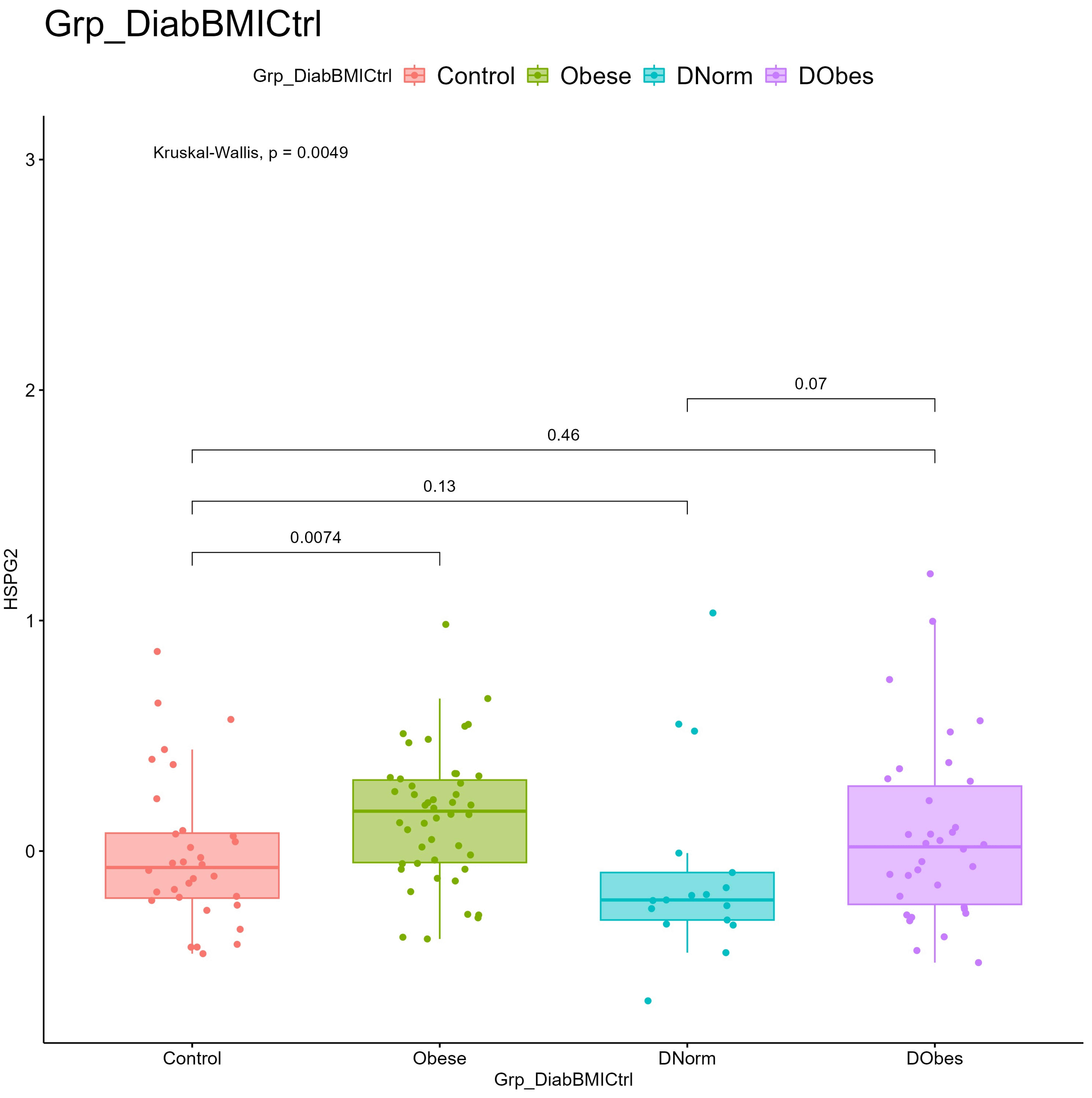
Grp_DiabBMICtrl
Grp_DiabBMICtrl
Control
Obese
DNorm
DObes
Kruskal-Wallis, p = 0.0049
0.07
0.46
0.13
0.0074
3
2
1
0
HSPG2
Control
Obese
DNorm
DObes
Grp_DiabBMICtrl

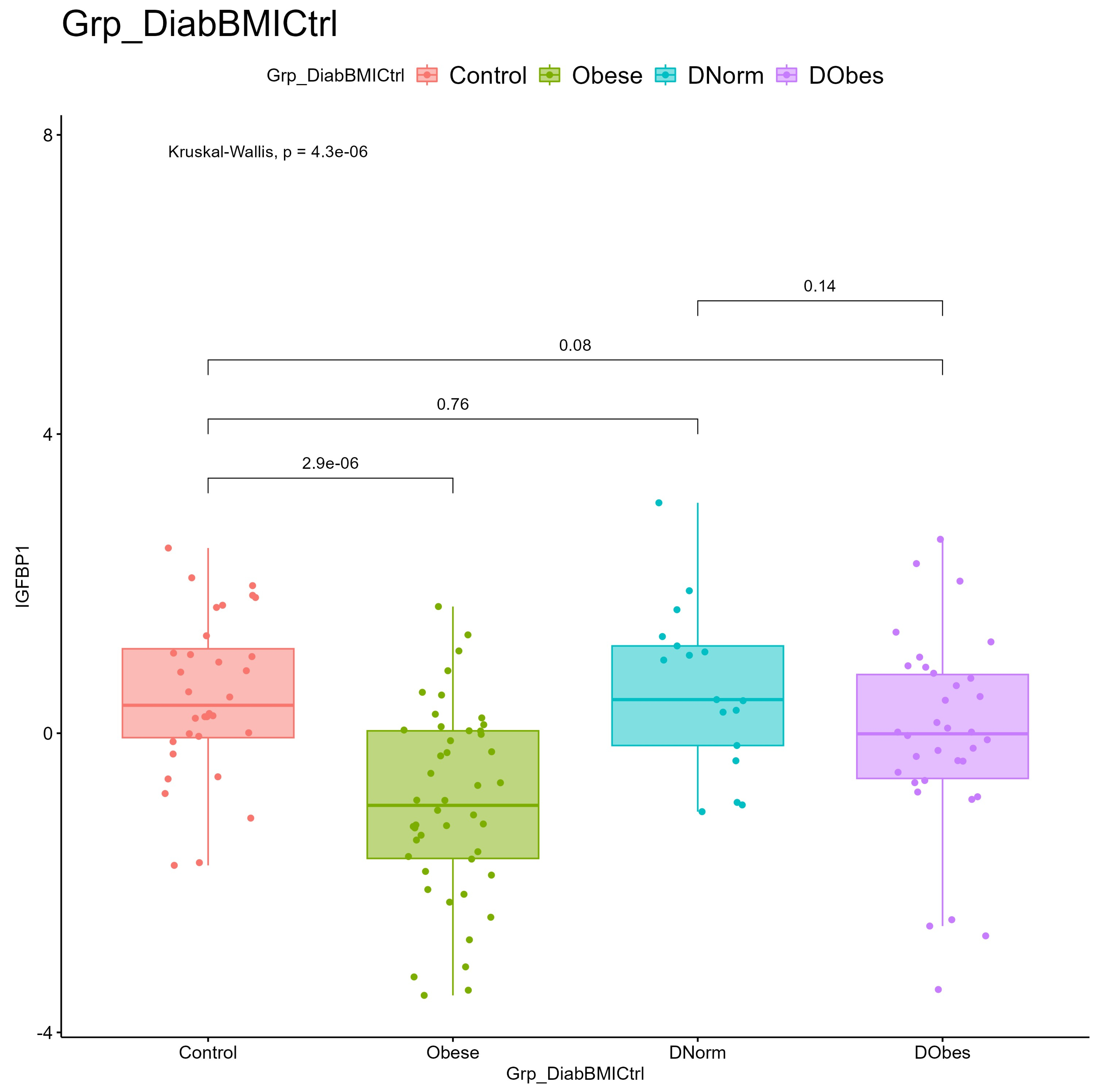

Grp_DiabBMICtrl
Grp_DiabBMICtrl
Control
Obese
DNorm
DObes
Kruskal-Wallis, p = 4.3e-06
0.14
0.08
0.76
2.9e-06
8
4
0
-4
IGFBP1
Control
Obese
DNorm
DObes
Grp_DiabBMICtrl

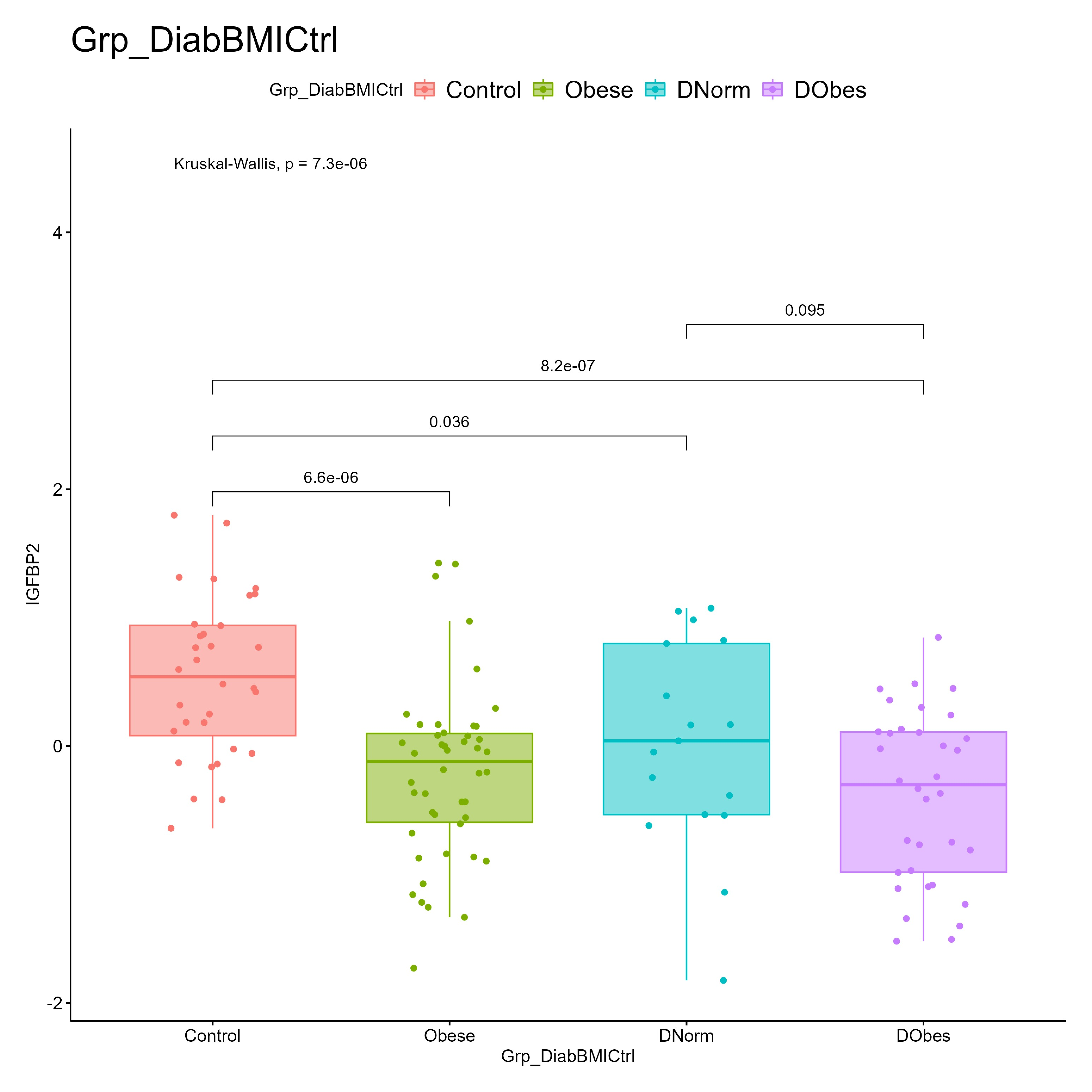

Grp_DiabBMICtrl
Grp_DiabBMICtrl
Control
Obese
DNorm
DObes
Kruskal-Wallis, p = 7.3e-06
0.095
8.2e-07
0.036
6.6e-06
IGFBP2
4
2
0
-2
Control
Obese
DNorm
DObes
Grp_DiabBMICtrl

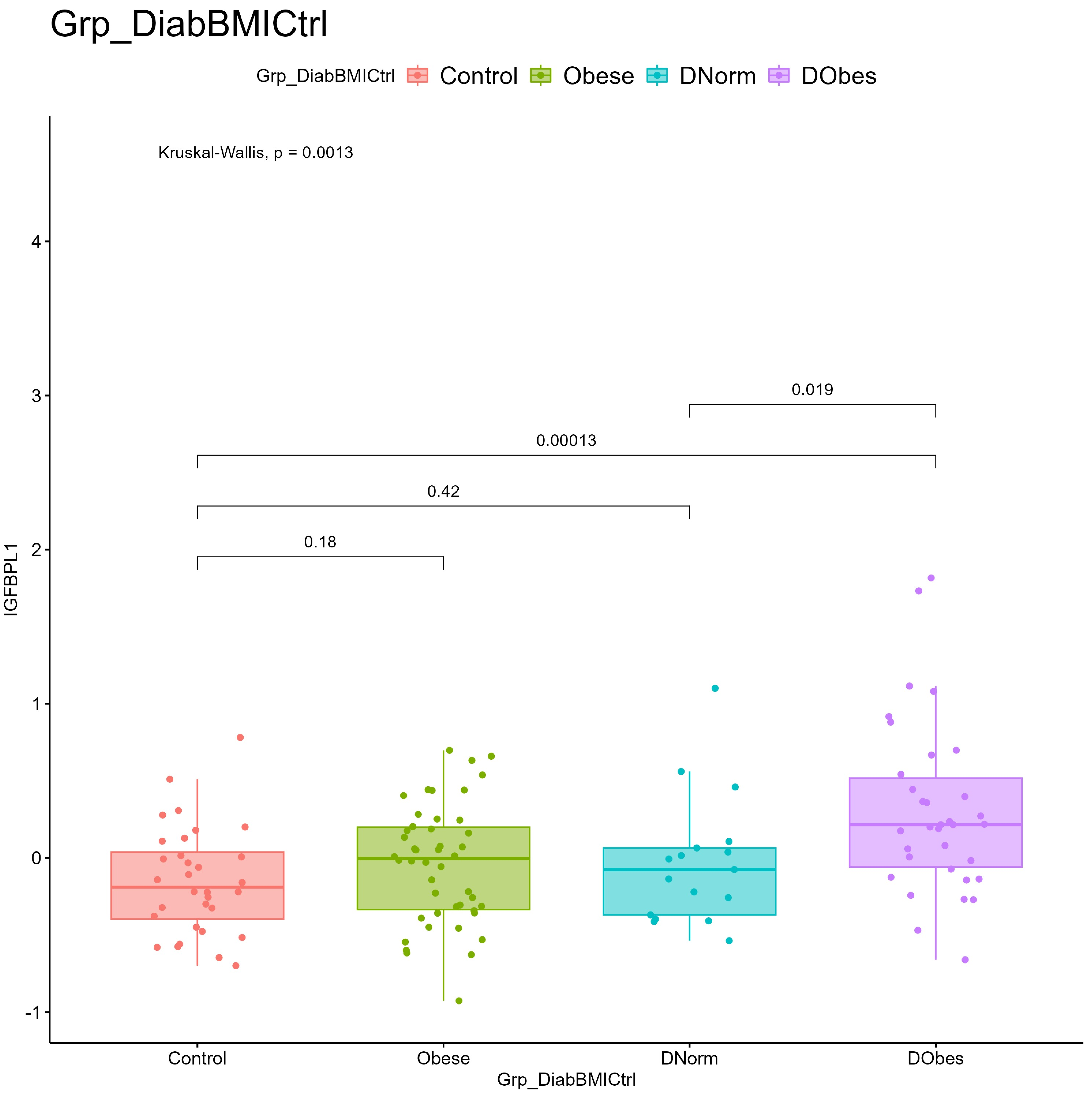
Grp_DiabBMICtrl
Grp_DiabBMICtrl
Control
Obese
DNorm
DObes
Kruskal-Wallis, p = 0.0013
0.019
0.00013
0.42
0.18
IGFBPL1
4
3
2
1
0
-1
Control
Obese
DNorm
DObes
Grp_DiabBMICtrl

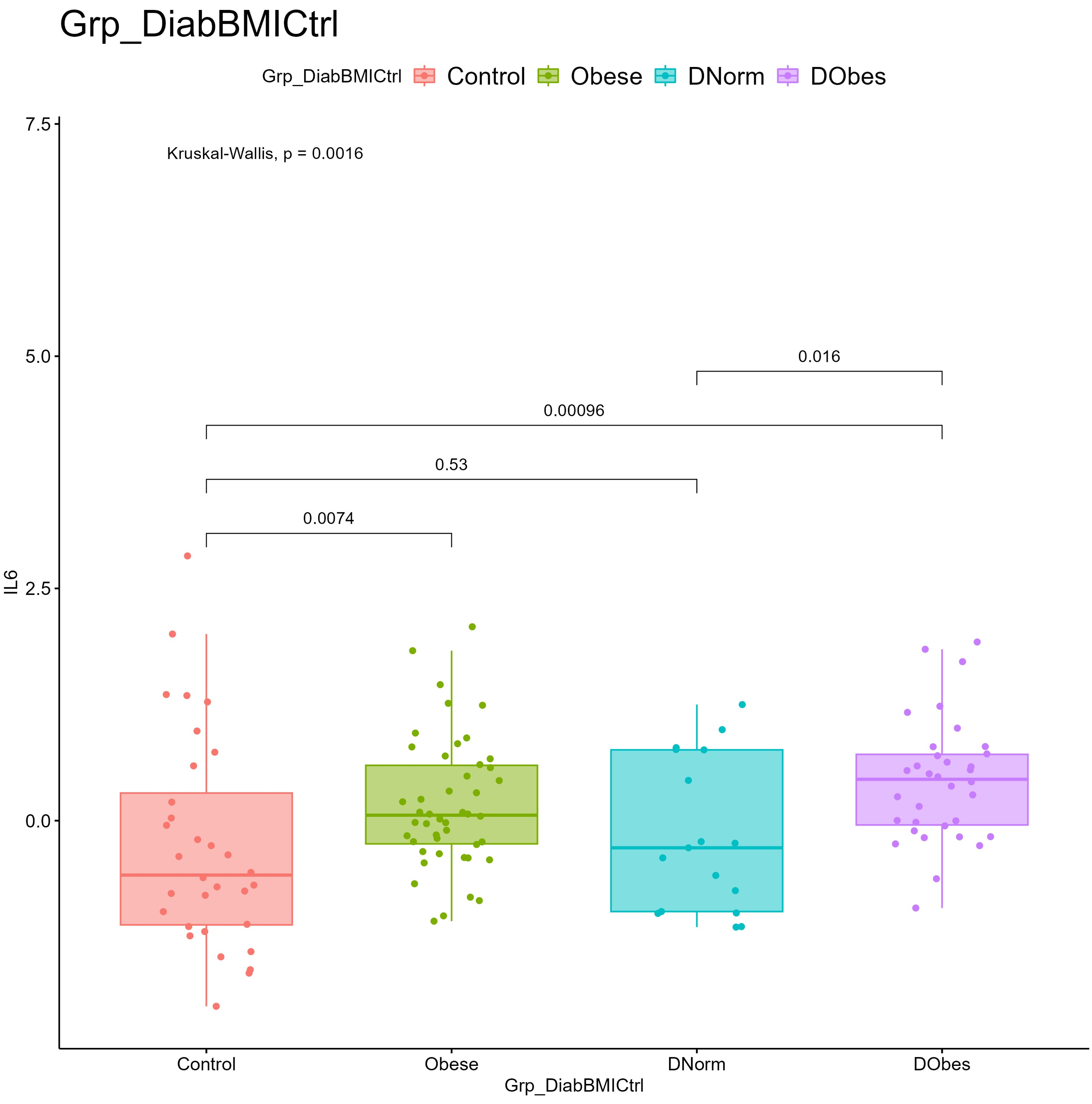

Grp_DiabBMICtrl
Grp_DiabBMICtrl
Control
Obese
DNorm
DObes
Kruskal-Wallis, p = 0.0016
0.016
0.00096
0.53
0.0074
7.5
5.0
2.5
0.0
IL6
Control
Obese
DNorm
DObes
Grp_DiabBMICtrl

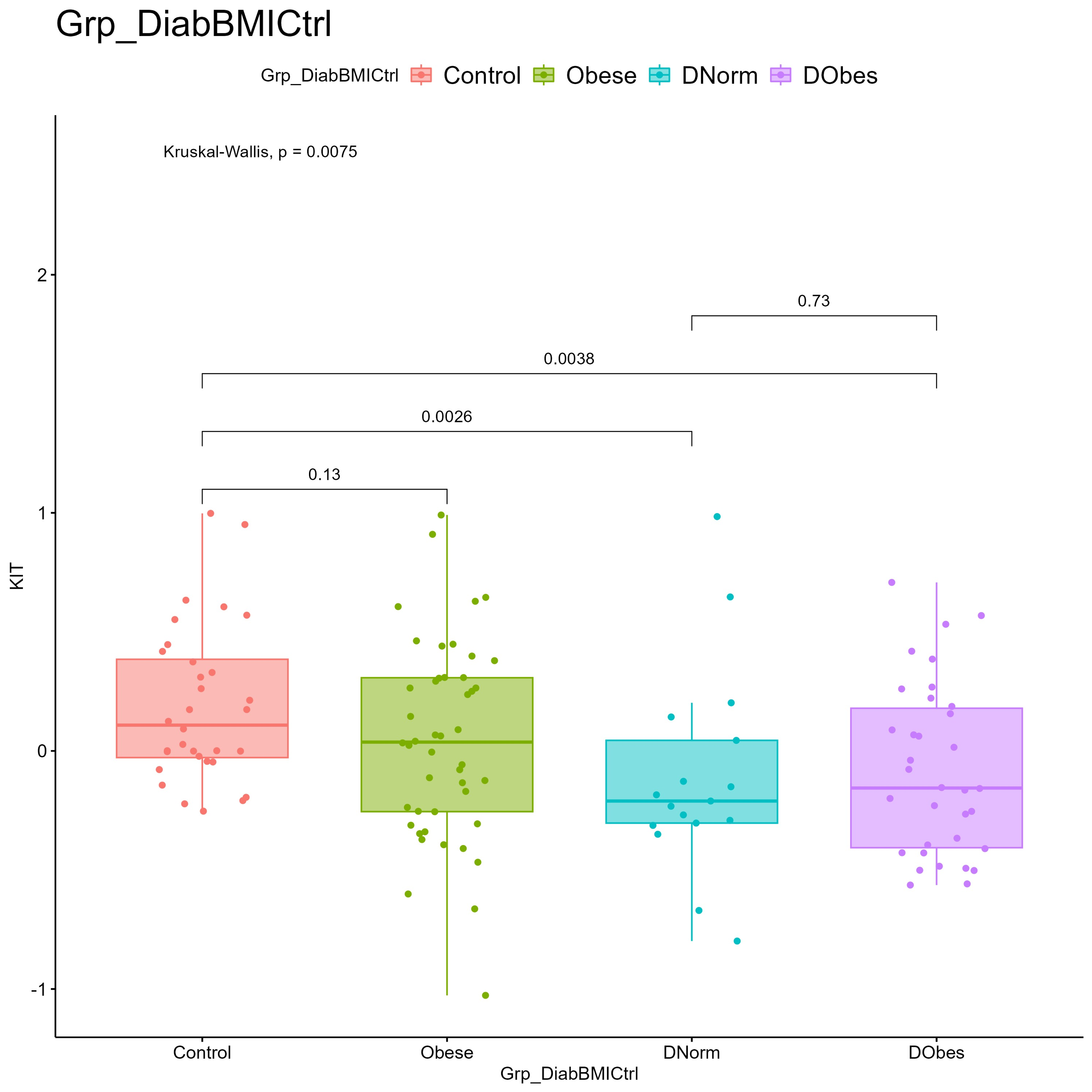
Grp_DiabBMICtrl
Grp_DiabBMICtrl Control Obese DNorm DObes
Kruskal-Wallis, p = 0.0075
0.73
0.0038
0.0026
0.13
KIT
2
1
0
-1
Control
Obese
DNorm
DObes
Grp_DiabBMICtrl

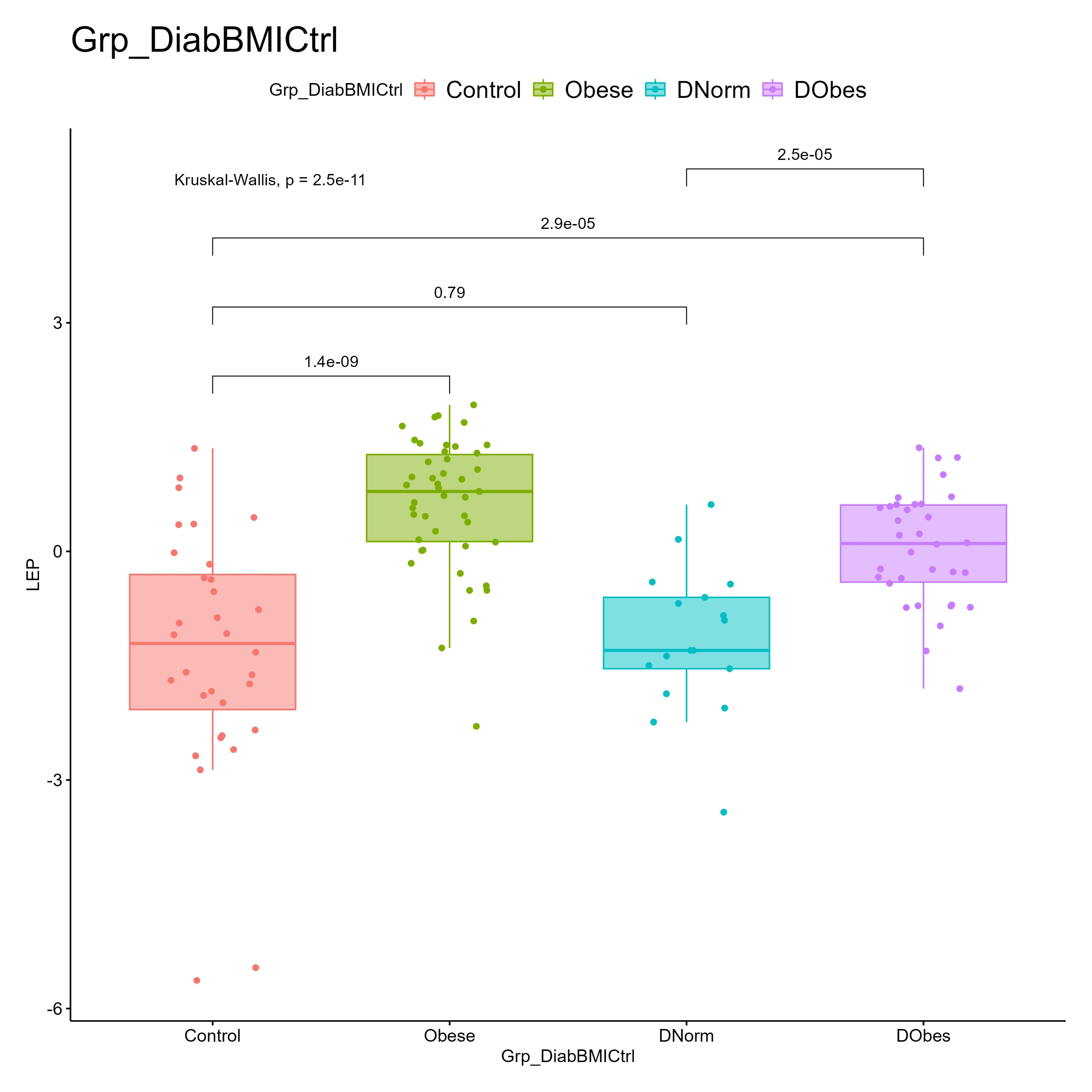

Grp_DiabBMICtrl
Grp_DiabBMICtrl
Control
Obese
DNorm
DObes
Kruskal-Wallis, p = 2.5e-11
2.5e-05
2.9e-05
0.79
1.4e-09
3
0
-3
-6
LEP
Control
Obese
DNorm
DObes
Grp_DiabBMICtrl

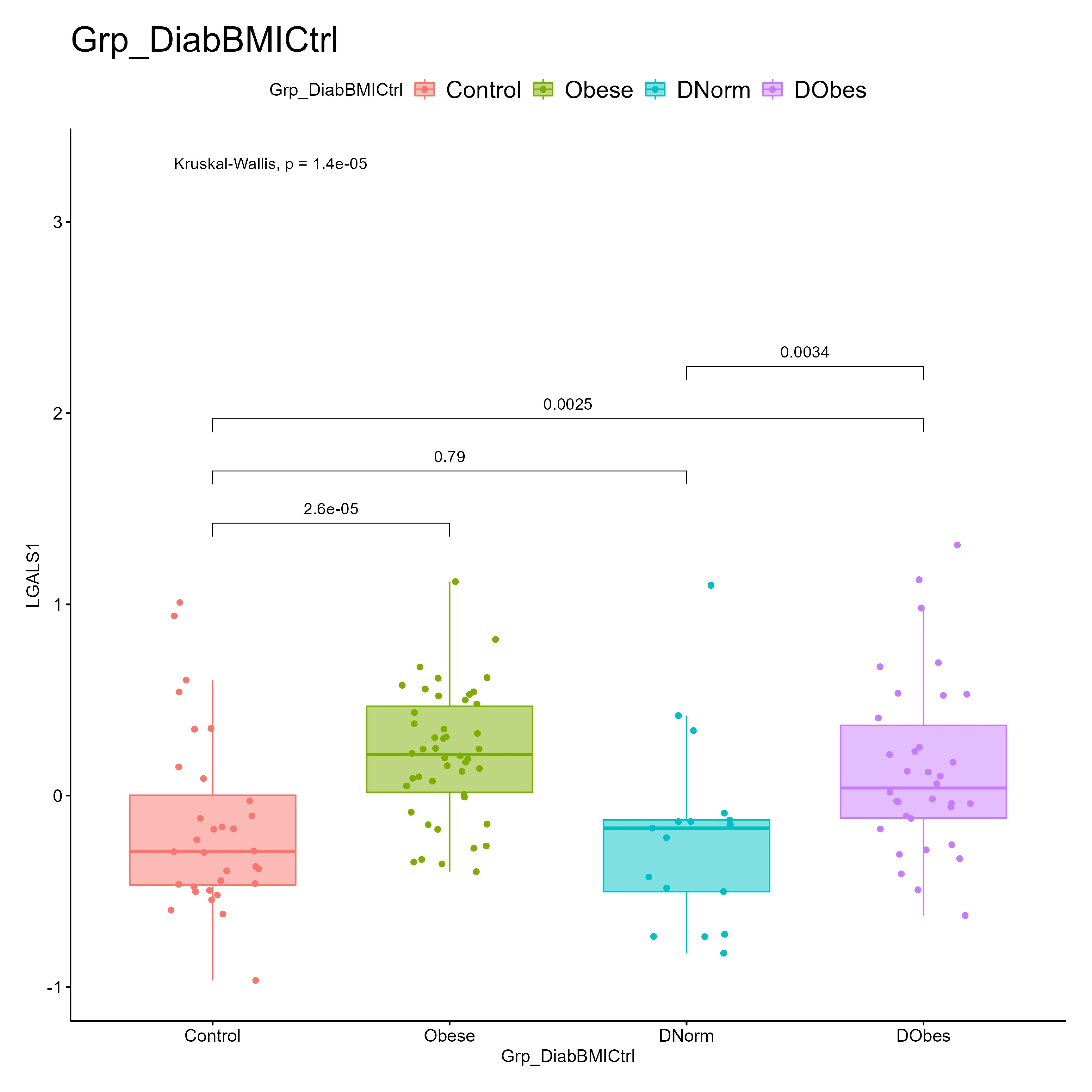
Grp_DiabBMICtrl
Grp_DiabBMICtrl
Control
Obese
DNorm
DObes
Kruskal-Wallis, p = 1.4e-05
0.0034
0.0025
0.79
2.6e-05
LGALS1
3
2
1
0
-1
Control
Obese
DNorm
DObes
Grp_DiabBMICtrl

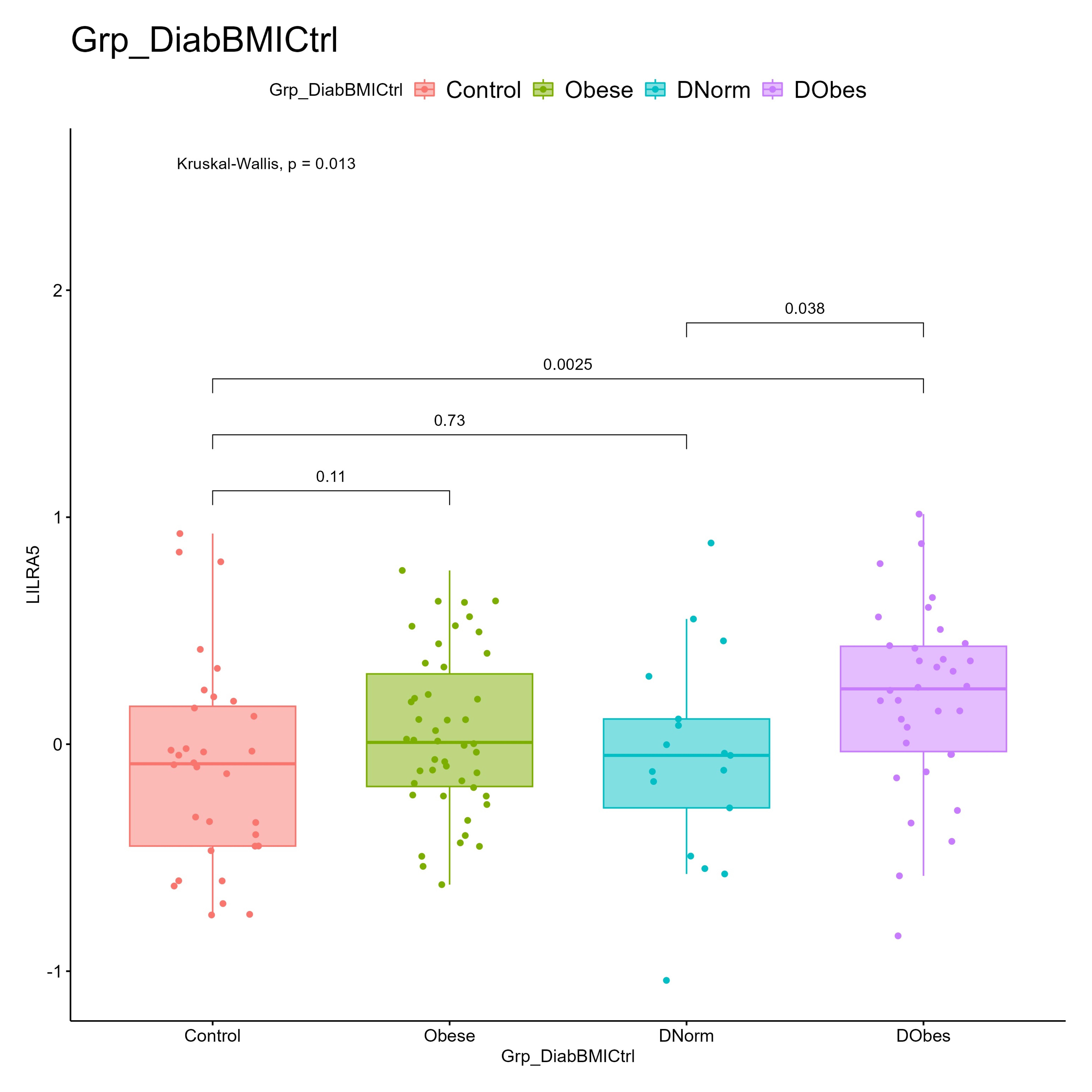
Grp_DiabBMICtrl
Grp_DiabBMICtrl
Control
Obese
DNorm
DObes
Kruskal-Wallis, p = 0.013
0.038
0.0025
0.73
0.11
2
1
0
-1
LILRA5
Control
Obese
DNorm
DObes
Grp_DiabBMICtrl

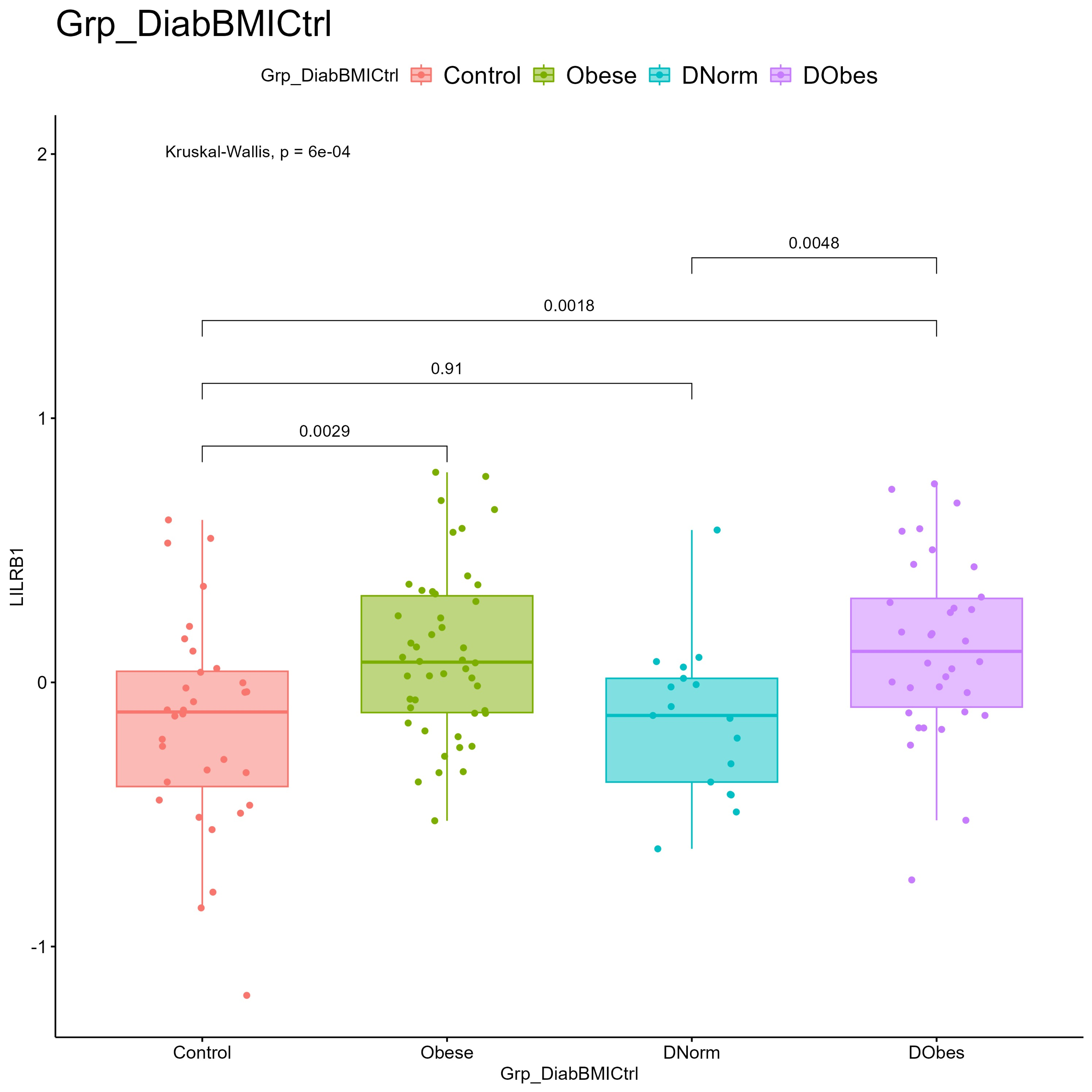

Grp_DiabBMICtrl
Grp_DiabBMICtrl Control Obese DNorm DObes
Kruskal-Wallis, p = 6e-04
0.0048
0.0018
0.91
0.0029
2
1
0
-1
LILRB1
Control
Obese
DNorm
DObes
Grp_DiabBMICtrl

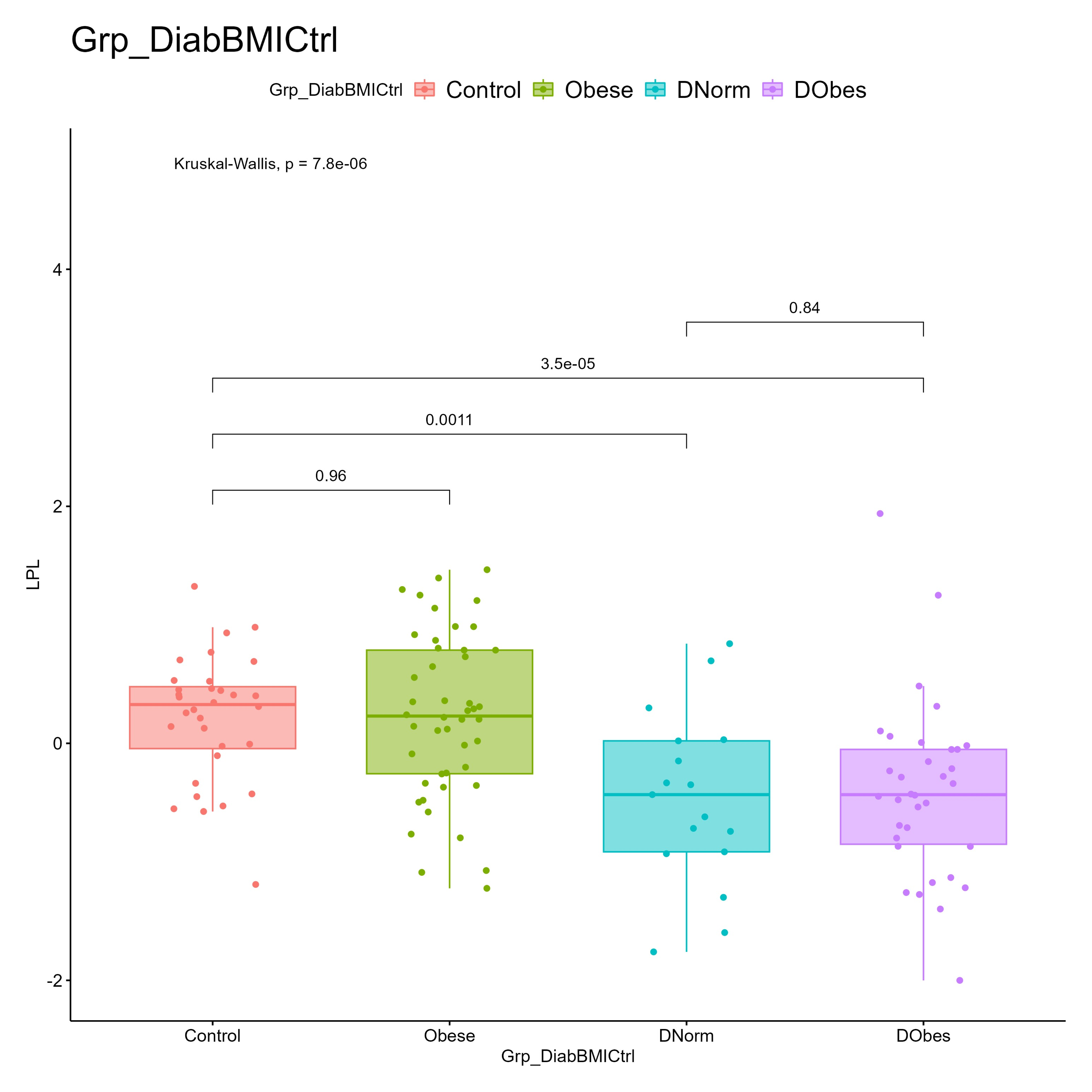

Grp_DiabBMICtrl
Grp_DiabBMICtrl
Control
Obese
DNorm
DObes
Kruskal-Wallis, p = 7.8e-06
0.84
3.5e-05
0.0011
0.96
LPL
4
2
0
-2
Control
Obese
DNorm
DObes
Grp_DiabBMICtrl

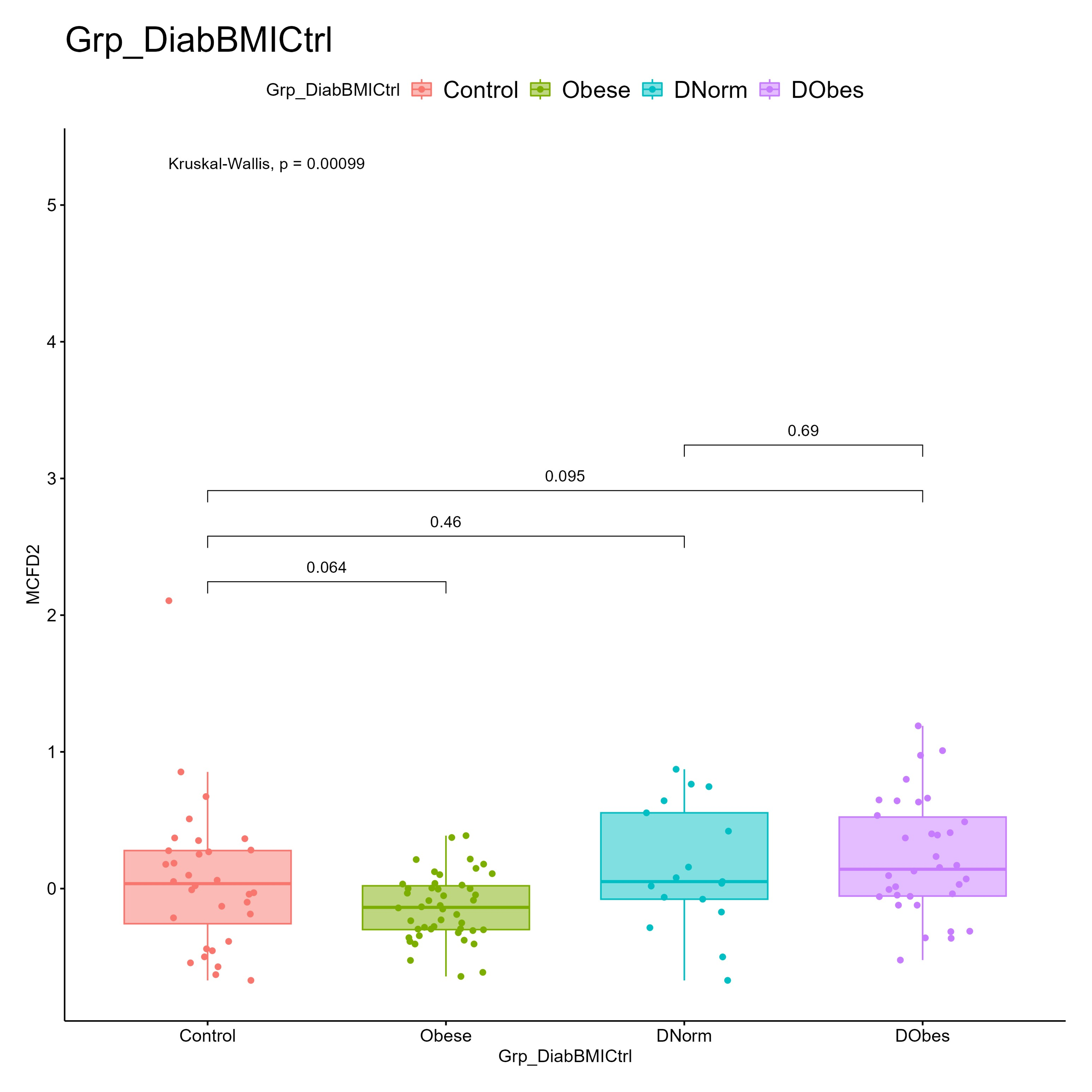

Grp_DiabBMICtrl
Grp_DiabBMICtrl
Control
Obese
DNorm
DObes
Kruskal-Wallis, p = 0.00099
0.69
0.095
0.46
0.064
MCFD2
5
4
3
2
1
0
Control
Obese
DNorm
DObes
Grp_DiabBMICtrl

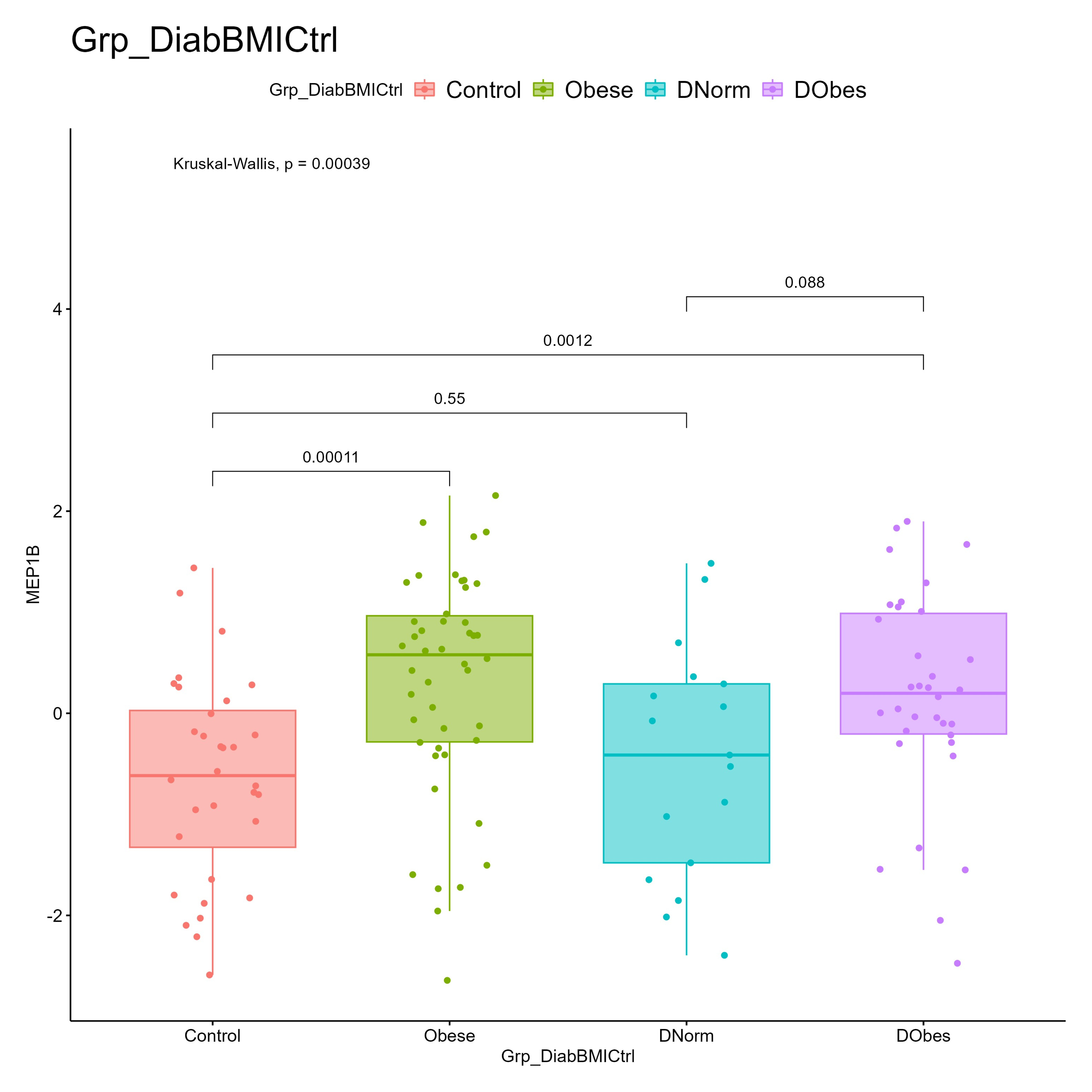

Grp_DiabBMICtrl
Grp_DiabBMICtrl
Control
Obese
DNorm
DObes
Kruskal-Wallis, p = 0.00039
0.088
0.0012
0.55
0.00011
MEP1B
4
2
0
-2
Control
Obese
DNorm
DObes
Grp_DiabBMICtrl

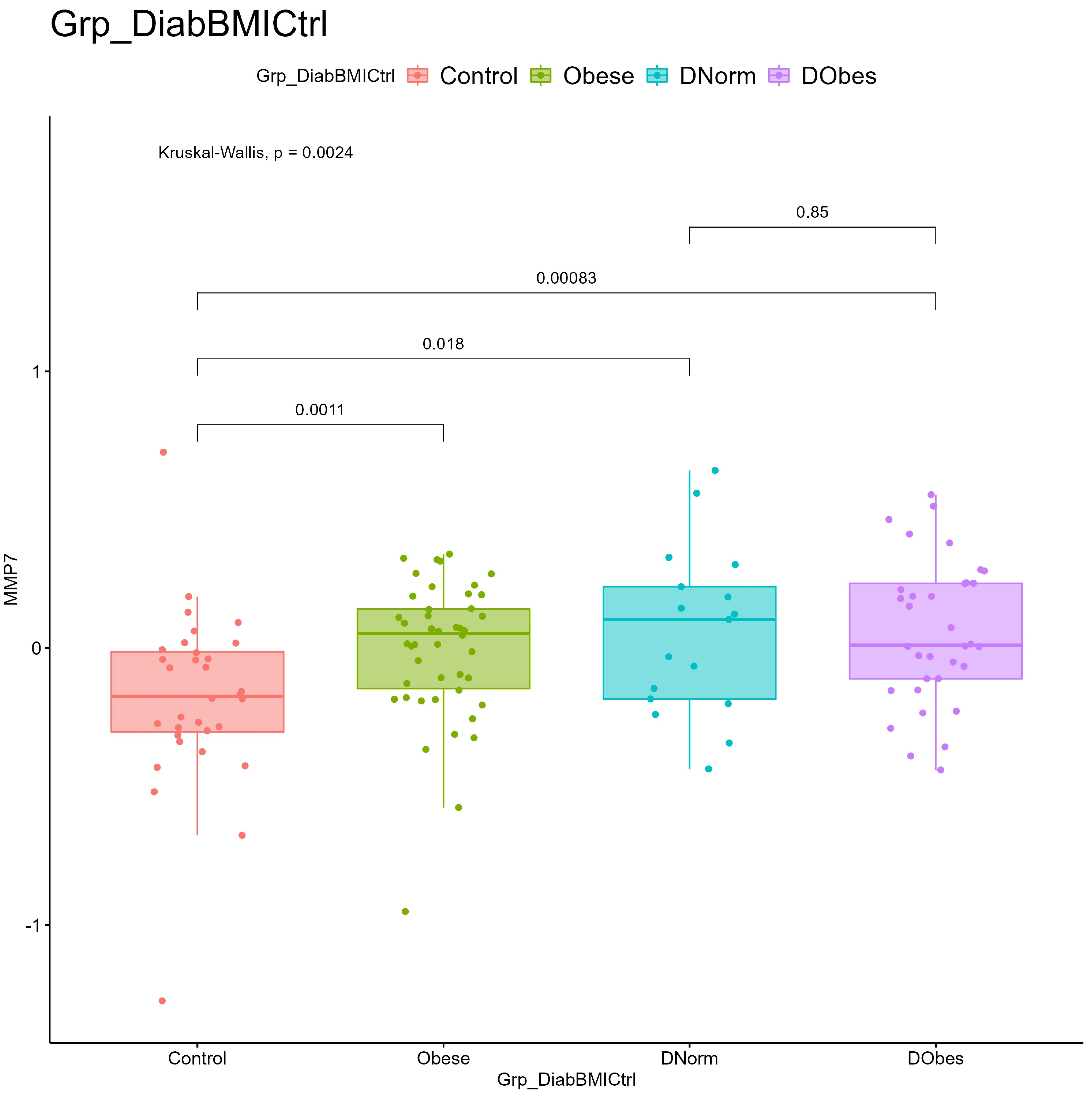
Grp_DiabBMICtrl
Grp_DiabBMICtrl
Control
Obese
DNorm
DObes
Kruskal-Wallis, p = 0.0024
0.85
0.00083
0.018
0.0011
MMP7
1
0
-1
Control
Obese
DNorm
DObes
Grp_DiabBMICtrl

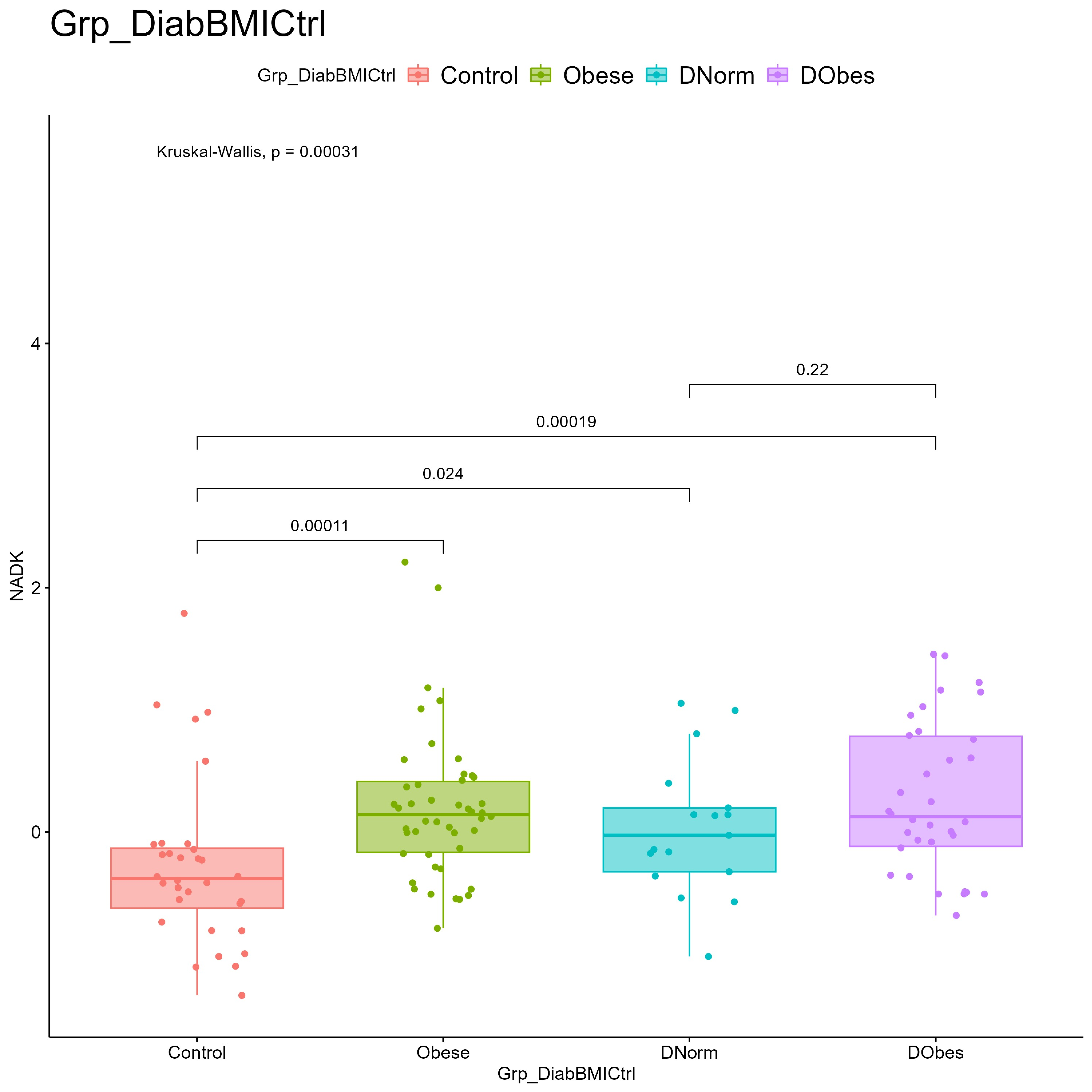
Grp_DiabBMICtrl
Grp_DiabBMICtrl
Control
Obese
DNorm
DObes
Kruskal-Wallis, p = 0.00031
0.22
0.00019
0.024
0.00011
NADK
4
2
0
Control
Obese
DNorm
DObes
Grp_DiabBMICtrl

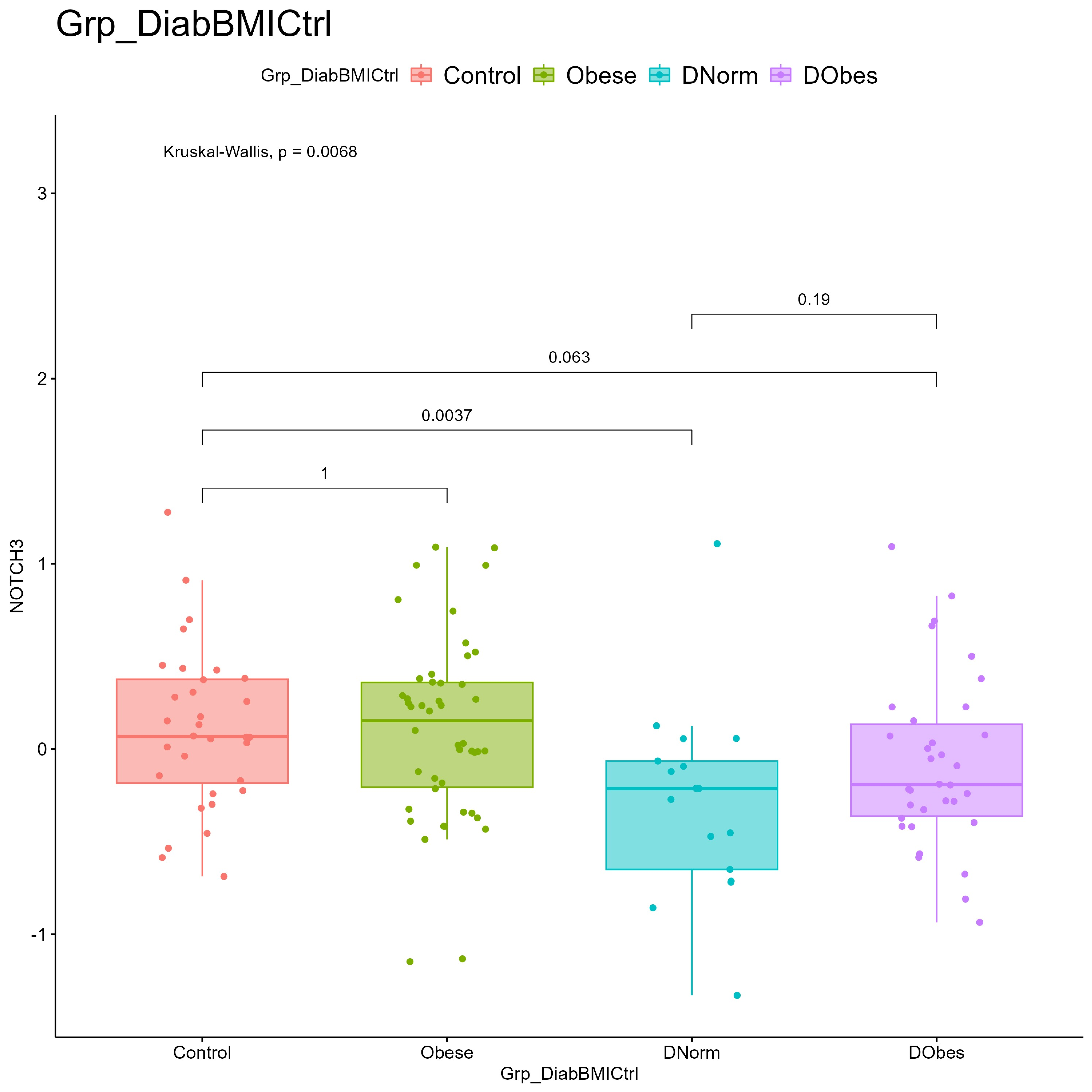

Grp_DiabBMICtrl
Grp_DiabBMICtrl Control Obese DNorm DObes
Kruskal-Wallis, p = 0.0068
0.19
0.063
0.0037
1
NOTCH3
3
2
1
0
-1
Control
Obese
DNorm
DObes
Grp_DiabBMICtrl

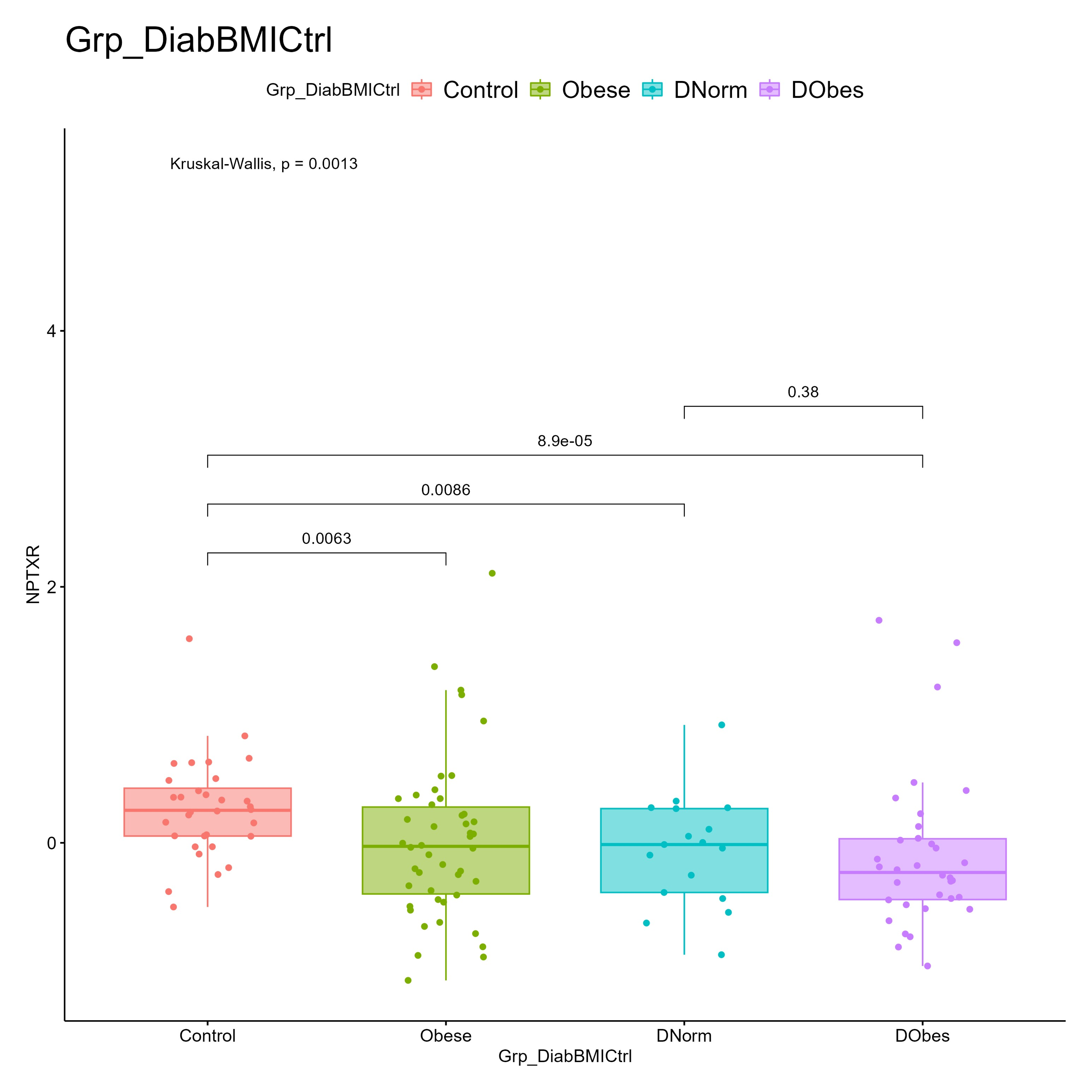

Grp_DiabBMICtrl
Grp_DiabBMICtrl
Control
Obese
DNorm
DObes
Kruskal-Wallis, p = 0.0013
0.38
8.9e-05
0.0086
0.0063
NPTXR
4
2
0
Control
Obese
DNorm
DObes
Grp_DiabBMICtrl

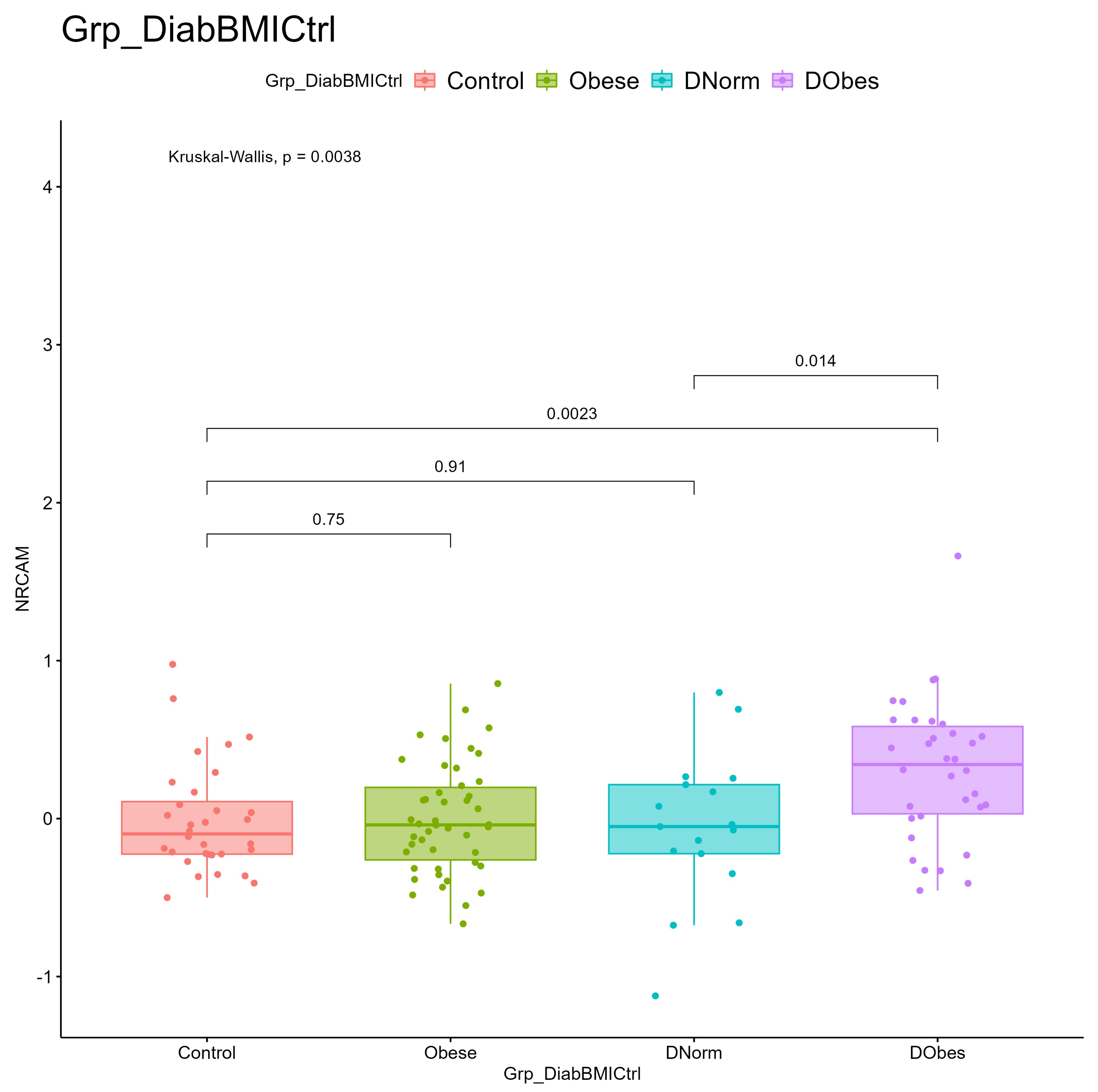
Grp_DiabBMICtrl
Grp_DiabBMICtrl
Control
Obese
DNorm
DObes
Kruskal-Wallis, p = 0.0038
0.014
0.0023
0.91
0.75
NRCAM
4
3
2
1
0
-1
Control
Obese
DNorm
DObes
Grp_DiabBMICtrl

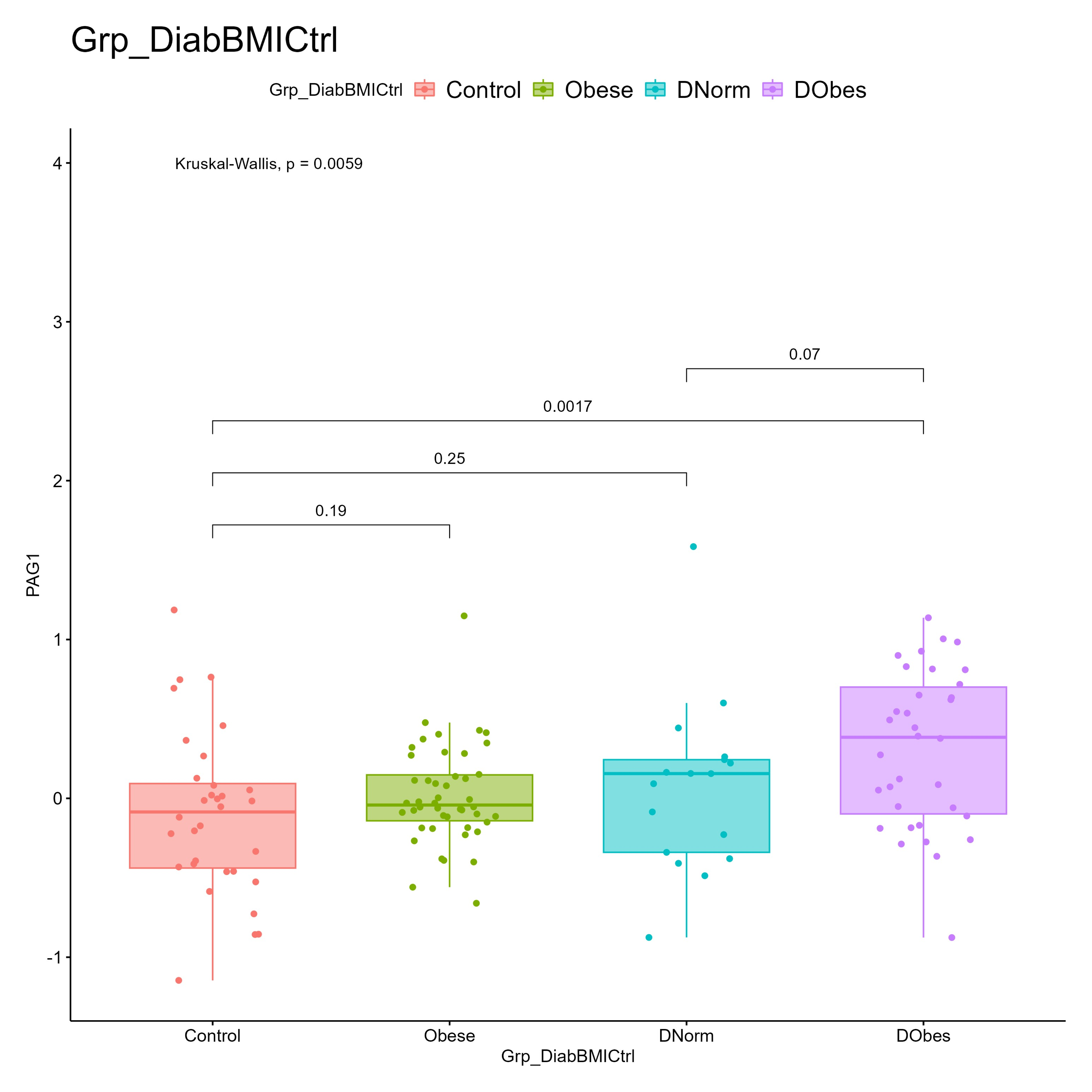

Grp_DiabBMICtrl
Grp_DiabBMICtrl
Control
Obese
DNorm
DObes
Kruskal-Wallis, p = 0.0059
0.07
0.0017
0.25
0.19
PAG1
4
3
2
1
0
-1
Control
Obese
DNorm
DObes
Grp_DiabBMICtrl

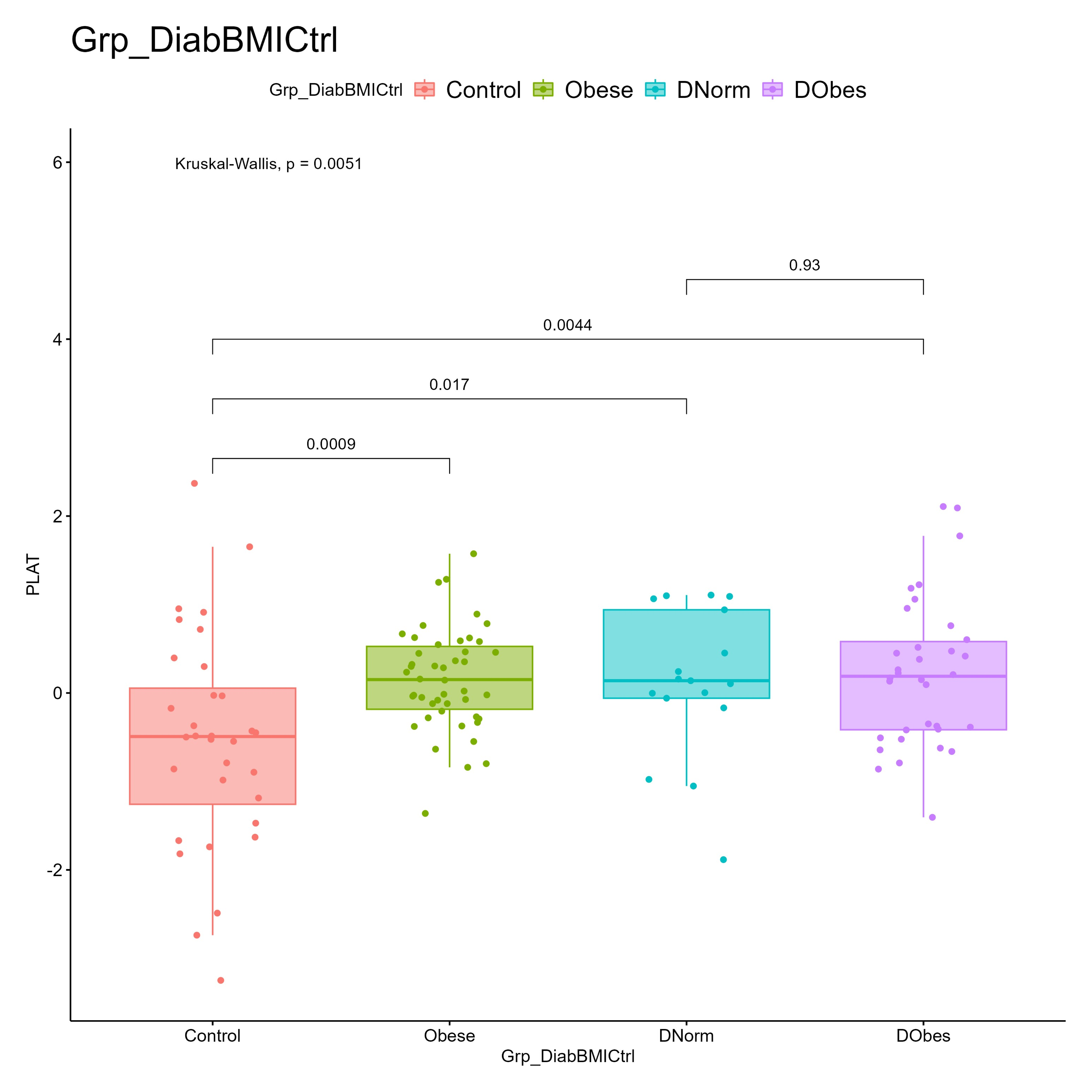

Grp_DiabBMICtrl
Grp_DiabBMICtrl
Control
Obese
DNorm
DObes
Kruskal-Wallis, p = 0.0051
0.93
0.0044
0.017
0.0009
PLAT
6
4
2
0
-2
Control
Obese
DNorm
DObes
Grp_DiabBMICtrl

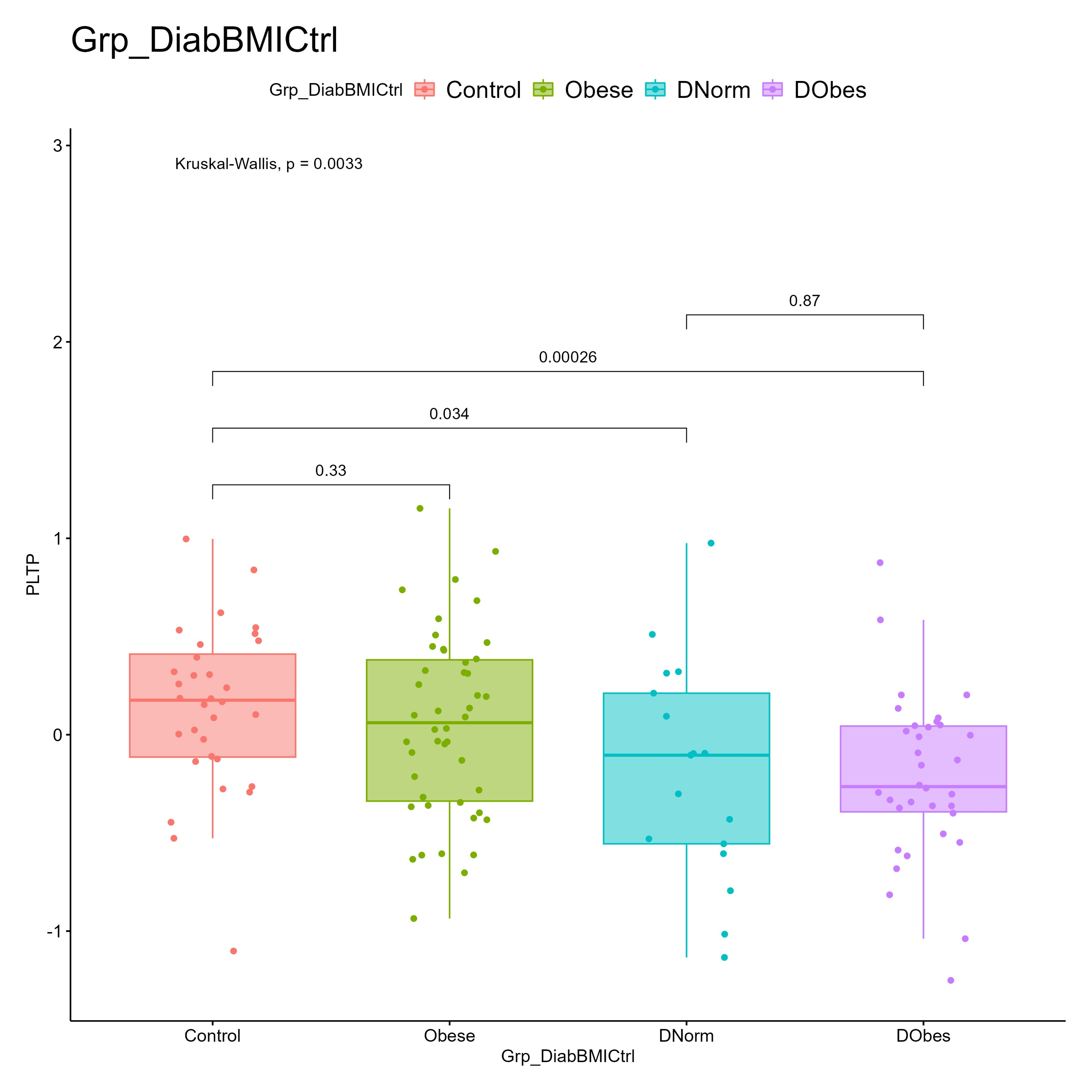
Grp_DiabBMICtrl
Grp_DiabBMICtrl
Control
Obese
DNorm
DObes
Kruskal-Wallis, p = 0.0033
0.87
0.00026
0.034
0.33
PLTP
3
2
1
0
-1
Control
Obese
DNorm
DObes
Grp_DiabBMICtrl

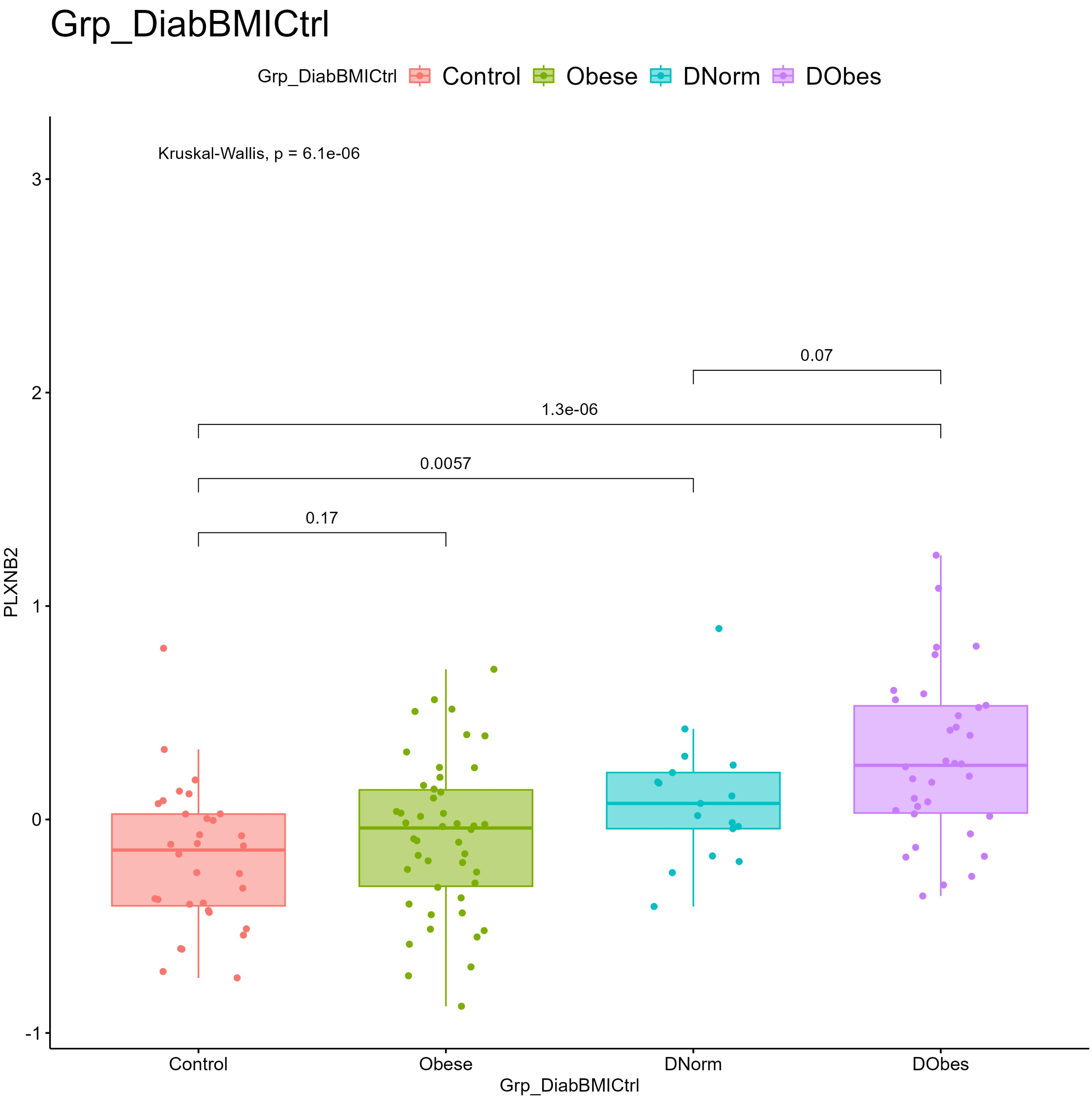

Grp_DiabBMICtrl
Grp_DiabBMICtrl
Control
Obese
DNorm
DObes
Kruskal-Wallis, p = 6.1e-06
0.07
1.3e-06
0.0057
0.17
PLXNB2
3
2
1
0
-1
Control
Obese
DNorm
DObes
Grp_DiabBMICtrl

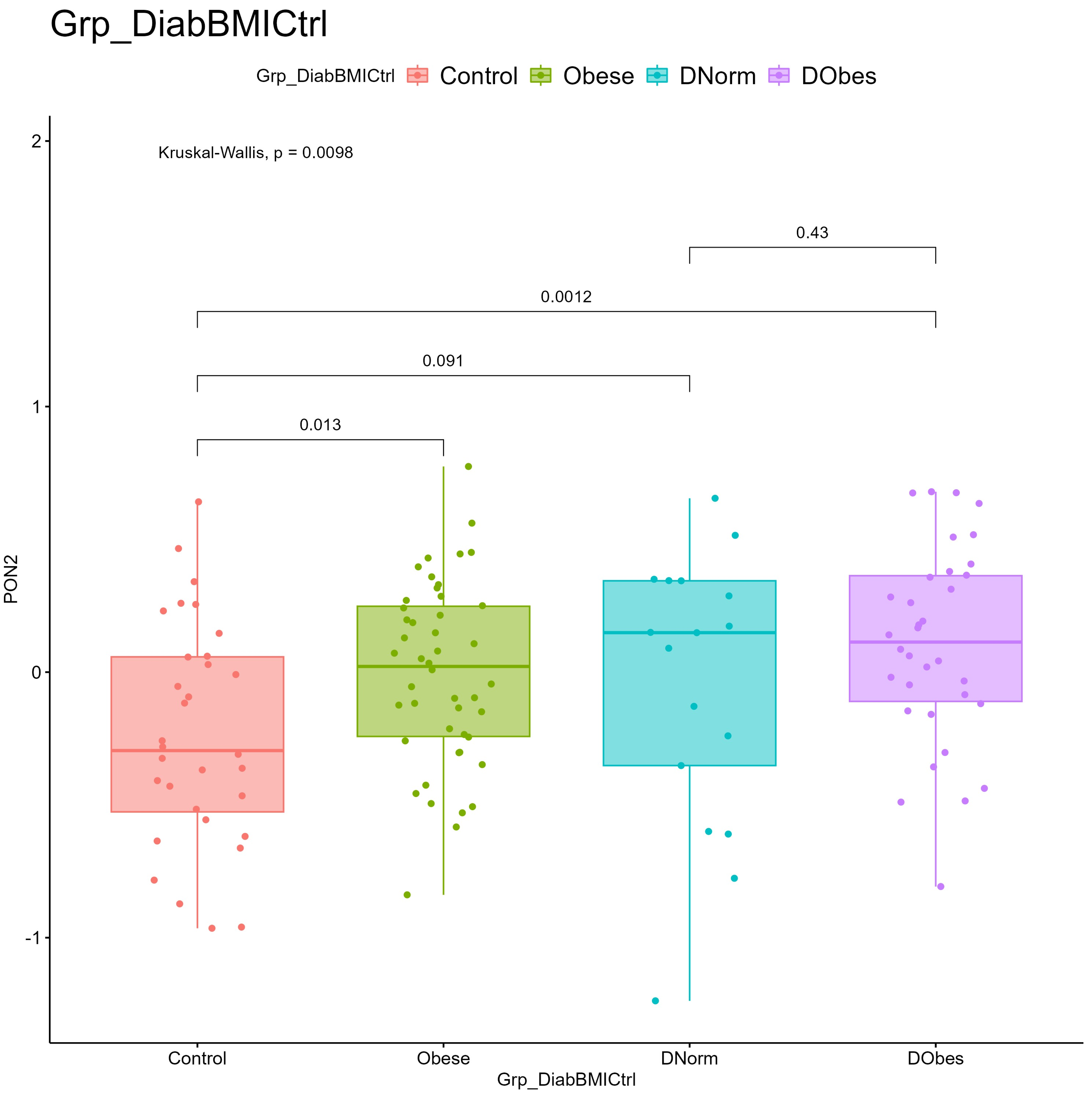

Grp_DiabBMICtrl
Grp_DiabBMICtrl
Control
Obese
DNorm
DObes
Kruskal-Wallis, p = 0.0098
0.43
0.0012
0.091
0.013
2
1
0
-1
PON2
Control
Obese
DNorm
DObes
Grp_DiabBMICtrl

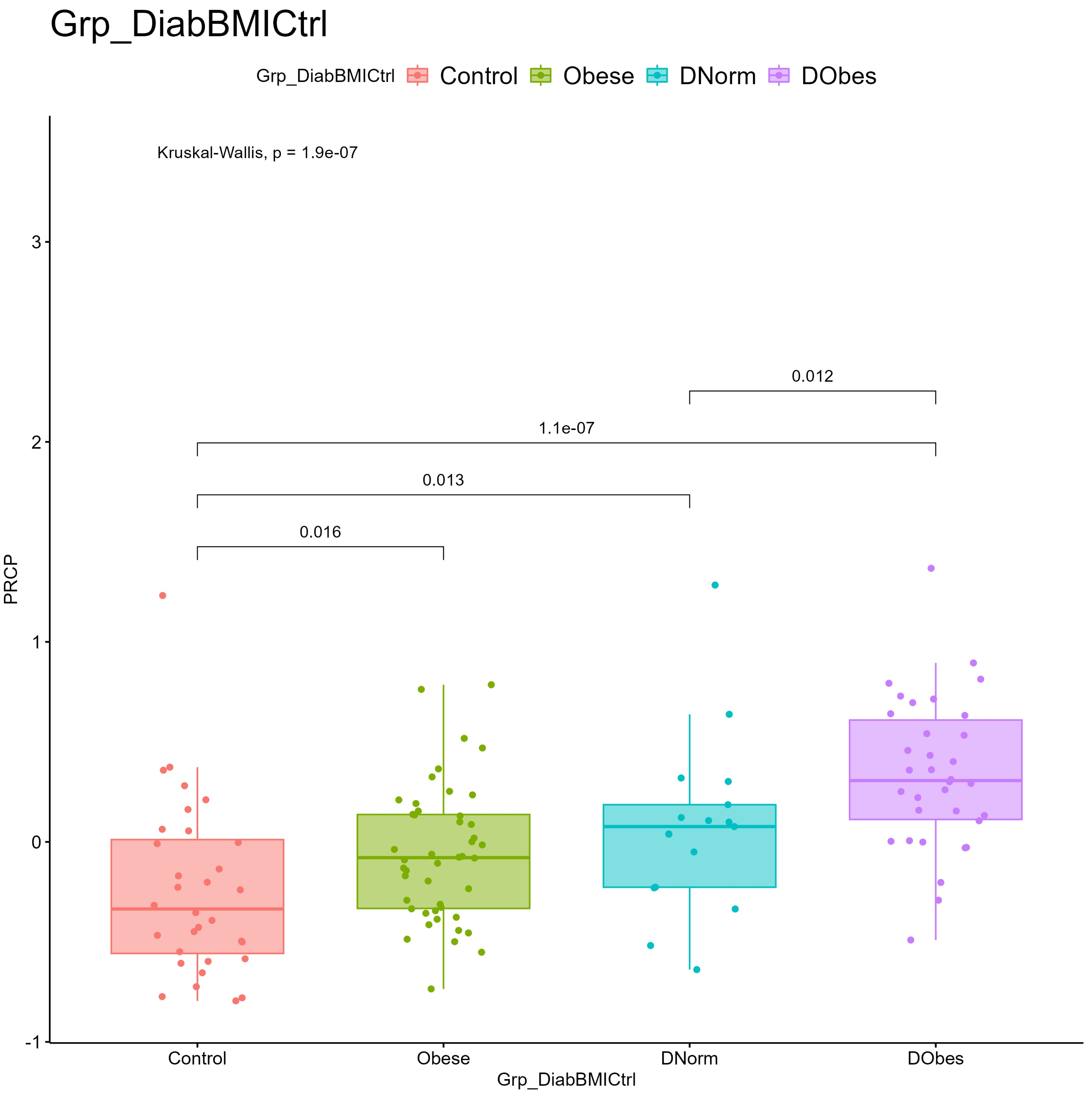

Grp_DiabBMICtrl
Grp_DiabBMICtrl Control Obese DNorm DObes
Kruskal-Wallis, p = 1.9e-07
0.012
1.1e-07
0.013
0.016
PRCP
3
2
1
0
-1
Control
Obese
DNorm
DObes
Grp_DiabBMICtrl

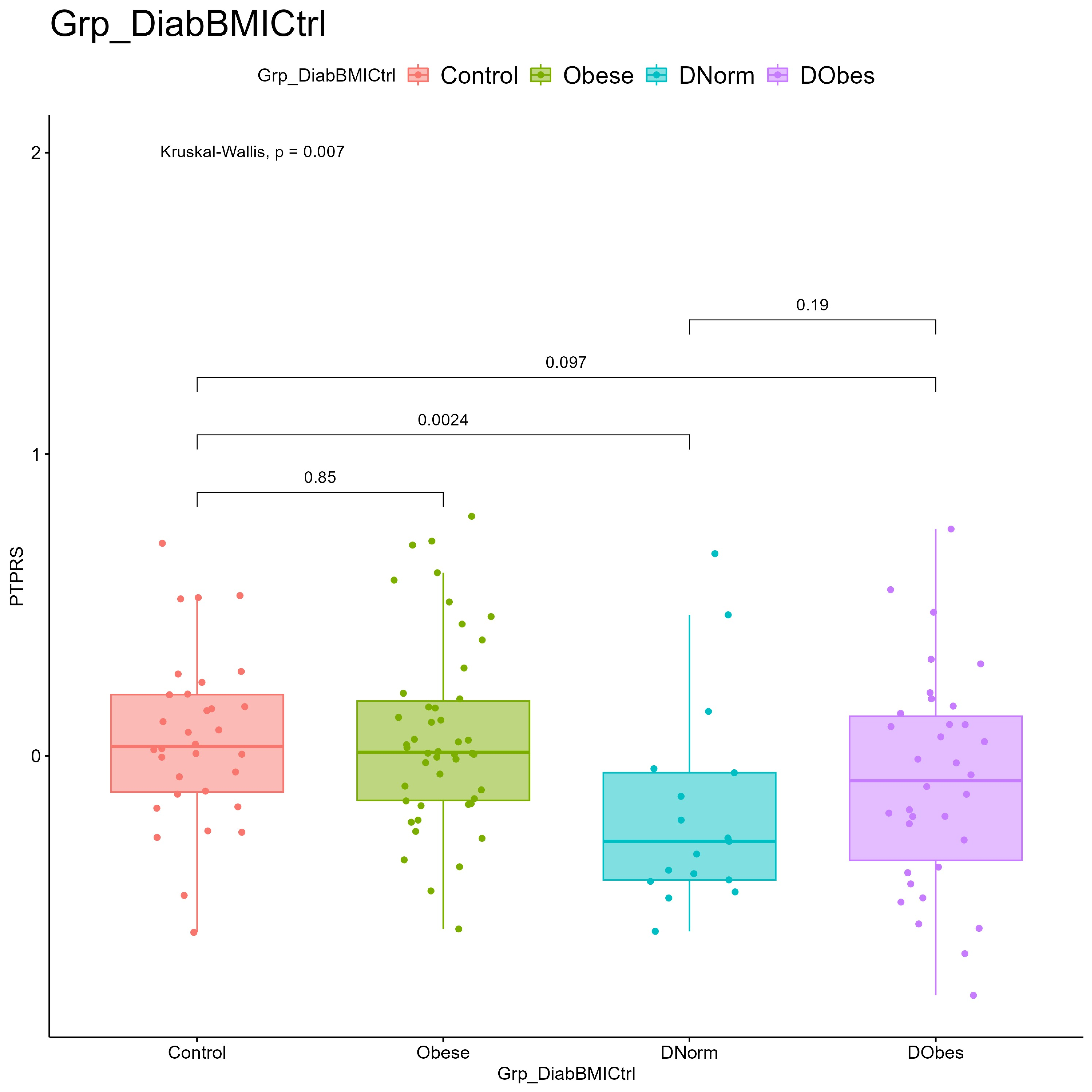
Grp_DiabBMICtrl
Grp_DiabBMICtrl
Control
Obese
DNorm
DObes
Kruskal-Wallis, p = 0.007
0.19
0.097
0.0024
0.85
2
1
0
PTPRS
Control
Obese
DNorm
DObes
Grp_DiabBMICtrl

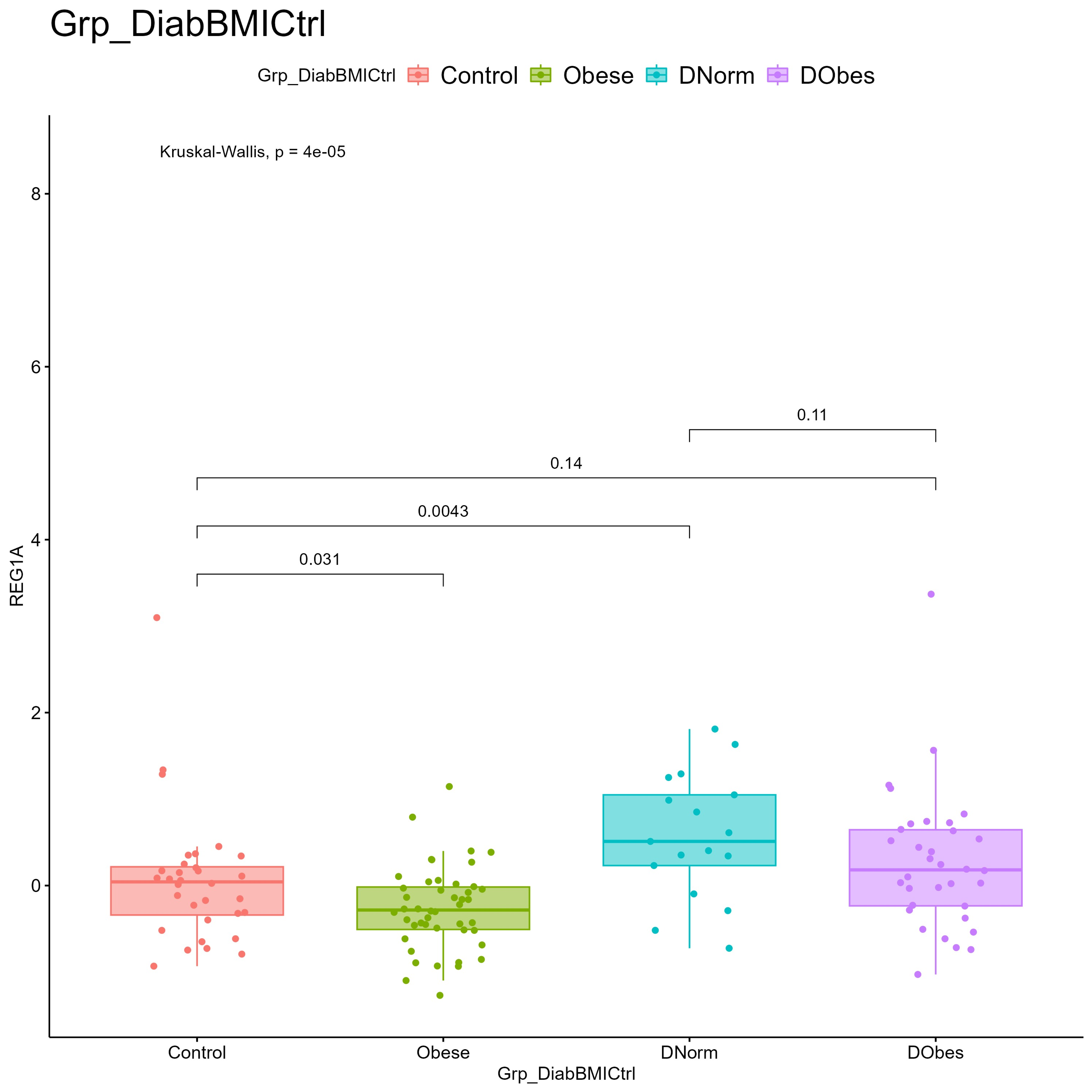

Grp_DiabBMICtrl
Grp_DiabBMICtrl
Control
Obese
DNorm
DObes
Kruskal-Wallis, p = 4e-05
0.11
0.14
0.0043
0.031
REG1A
8
6
4
2
0
Control
Obese
DNorm
DObes
Grp_DiabBMICtrl

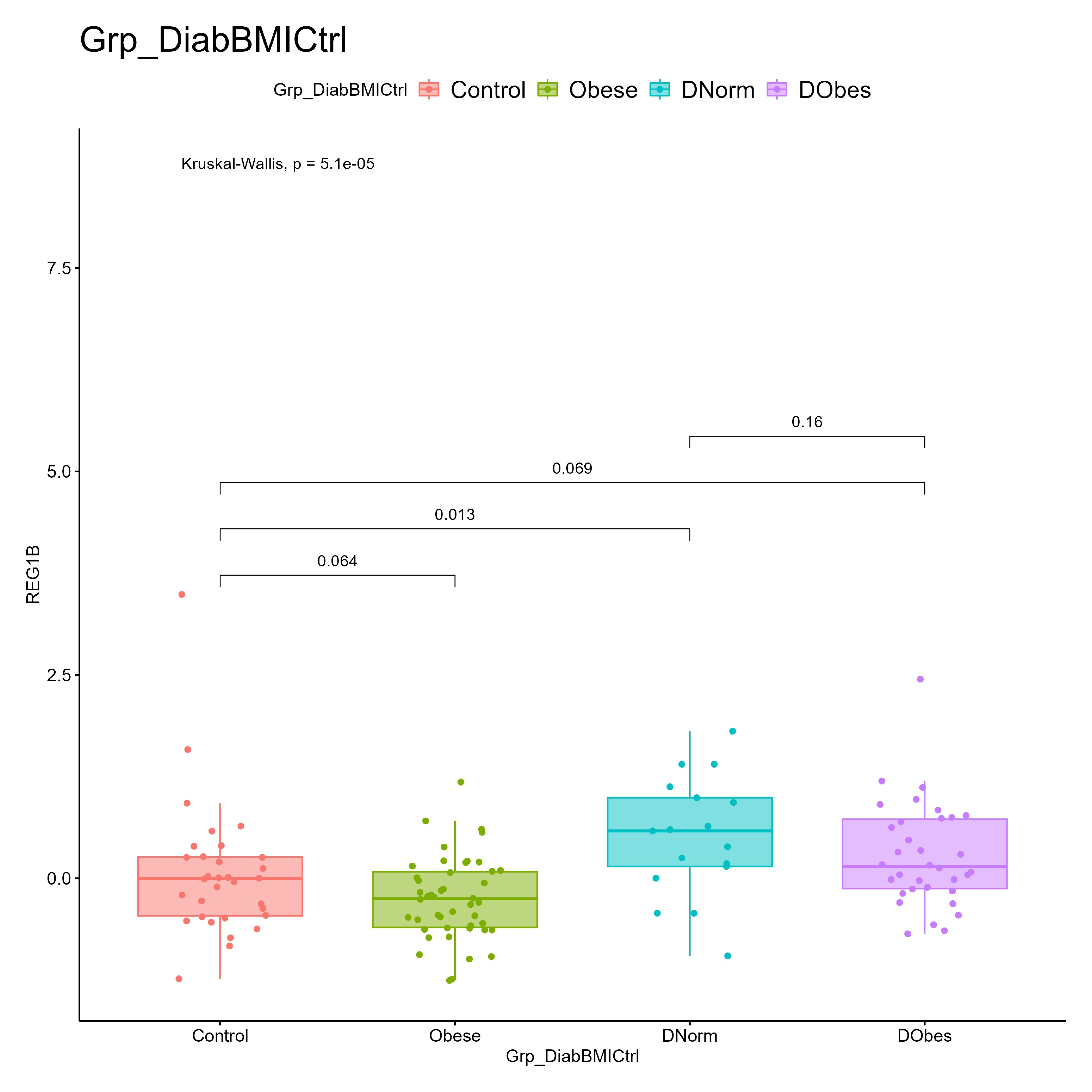
Grp_DiabBMICtrl
Grp_DiabBMICtrl
Control
Obese
DNorm
DObes
Kruskal-Wallis, p = 5.1e-05
0.16
0.069
0.013
0.064
7.5
5.0
2.5
0.0
REG1B
Control
Obese
DNorm
DObes
Grp_DiabBMICtrl

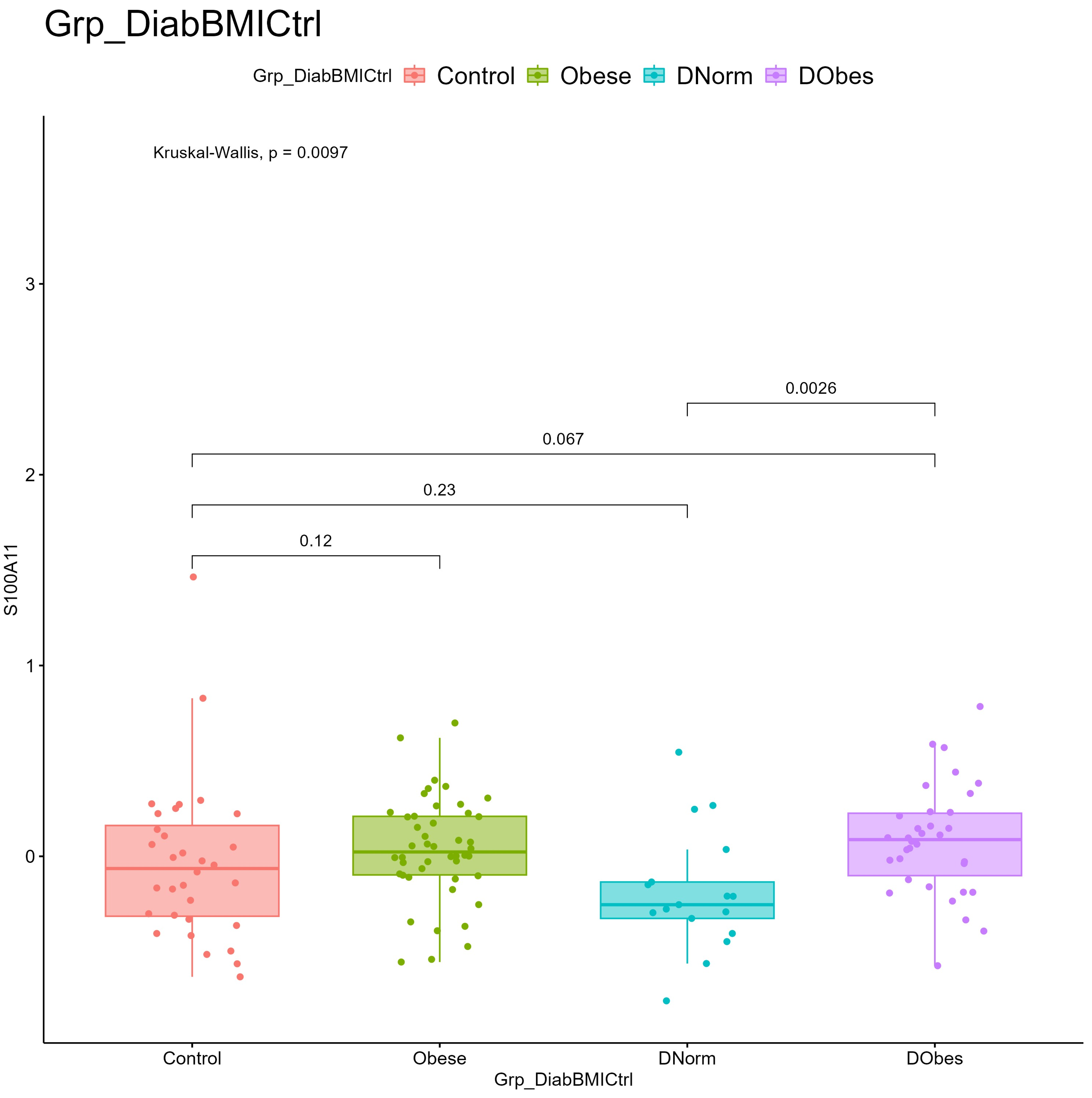
Grp_DiabBMICtrl
Grp_DiabBMICtrl
Control
Obese
DNorm
DObes
Kruskal-Wallis, p = 0.0097
0.0026
0.067
0.23
0.12
S100A11
3
2
1
0
Control
Obese
DNorm
DObes
Grp_DiabBMICtrl

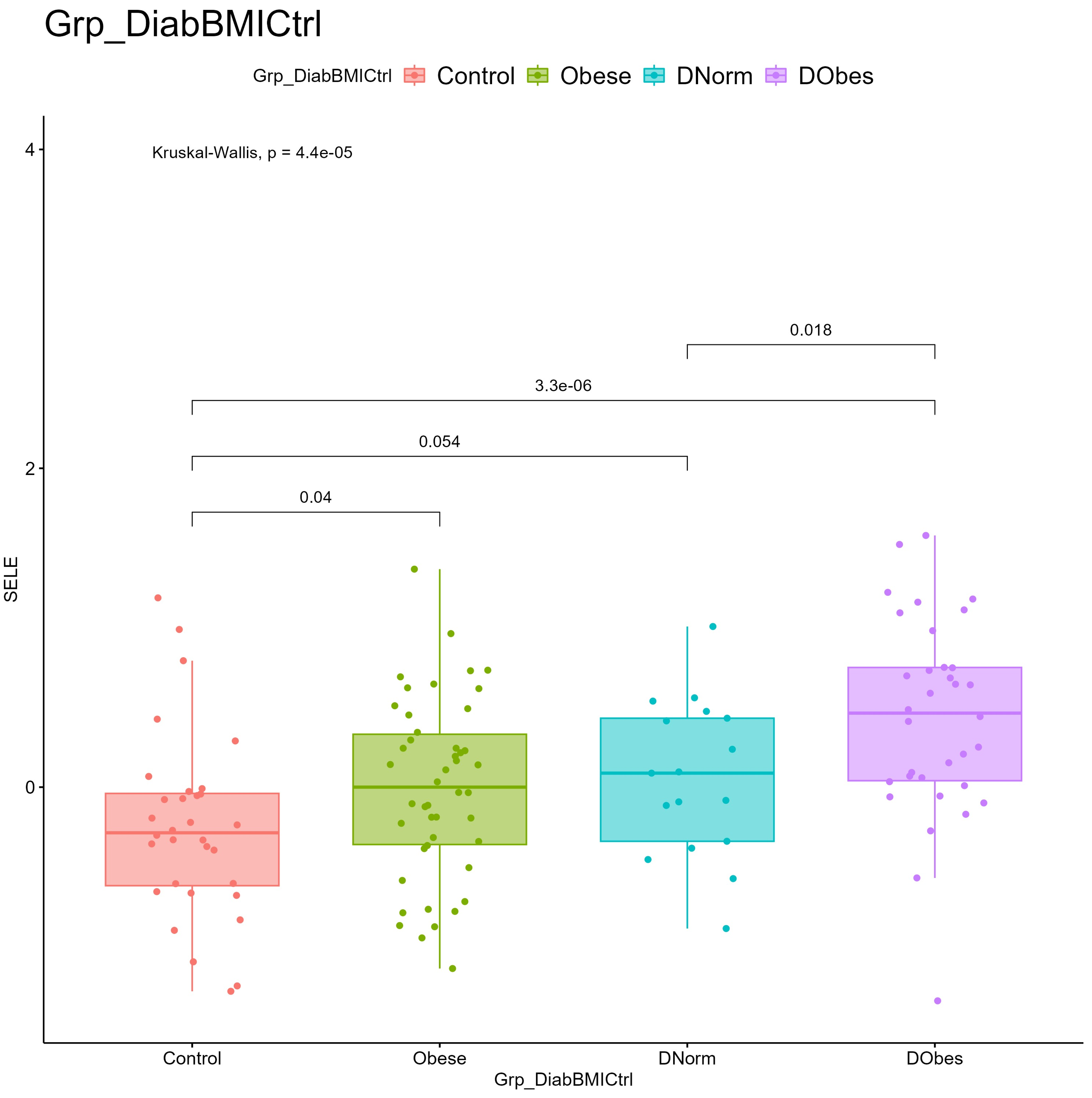

Grp_DiabBMICtrl
Grp_DiabBMICtrl
Control
Obese
DNorm
DObes
Kruskal-Wallis, p = 4.4e-05
0.018
3.3e-06
0.054
0.04
SELE
4
2
0
Control
Obese
DNorm
DObes
Grp_DiabBMICtrl

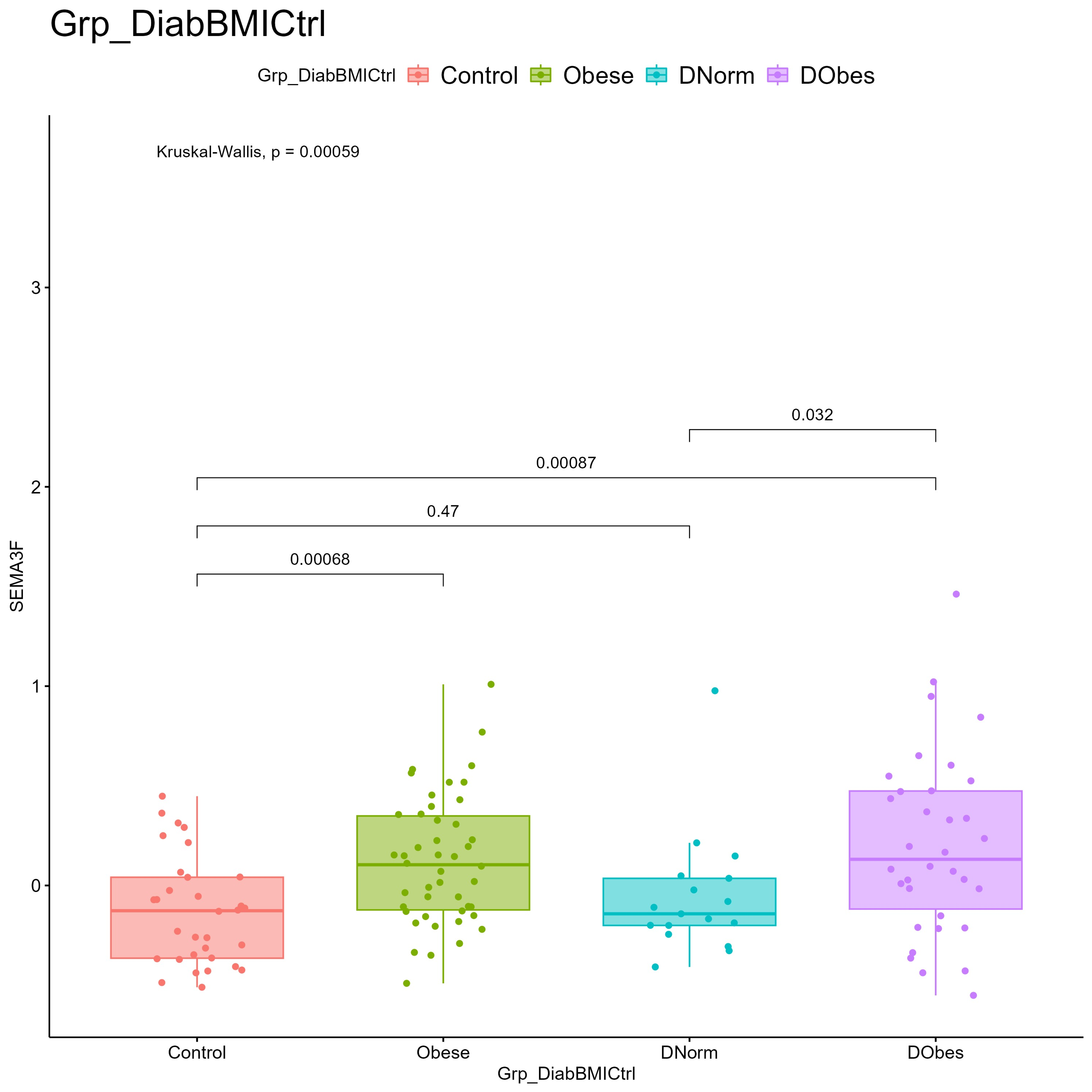
Grp_DiabBMICtrl
Grp_DiabBMICtrl
Control
Obese
DNorm
DObes
Kruskal-Wallis, p = 0.00059
0.032
0.00087
0.47
0.00068
SEMA3F
3
2
1
0
Control
Obese
DNorm
DObes
Grp_DiabBMICtrl

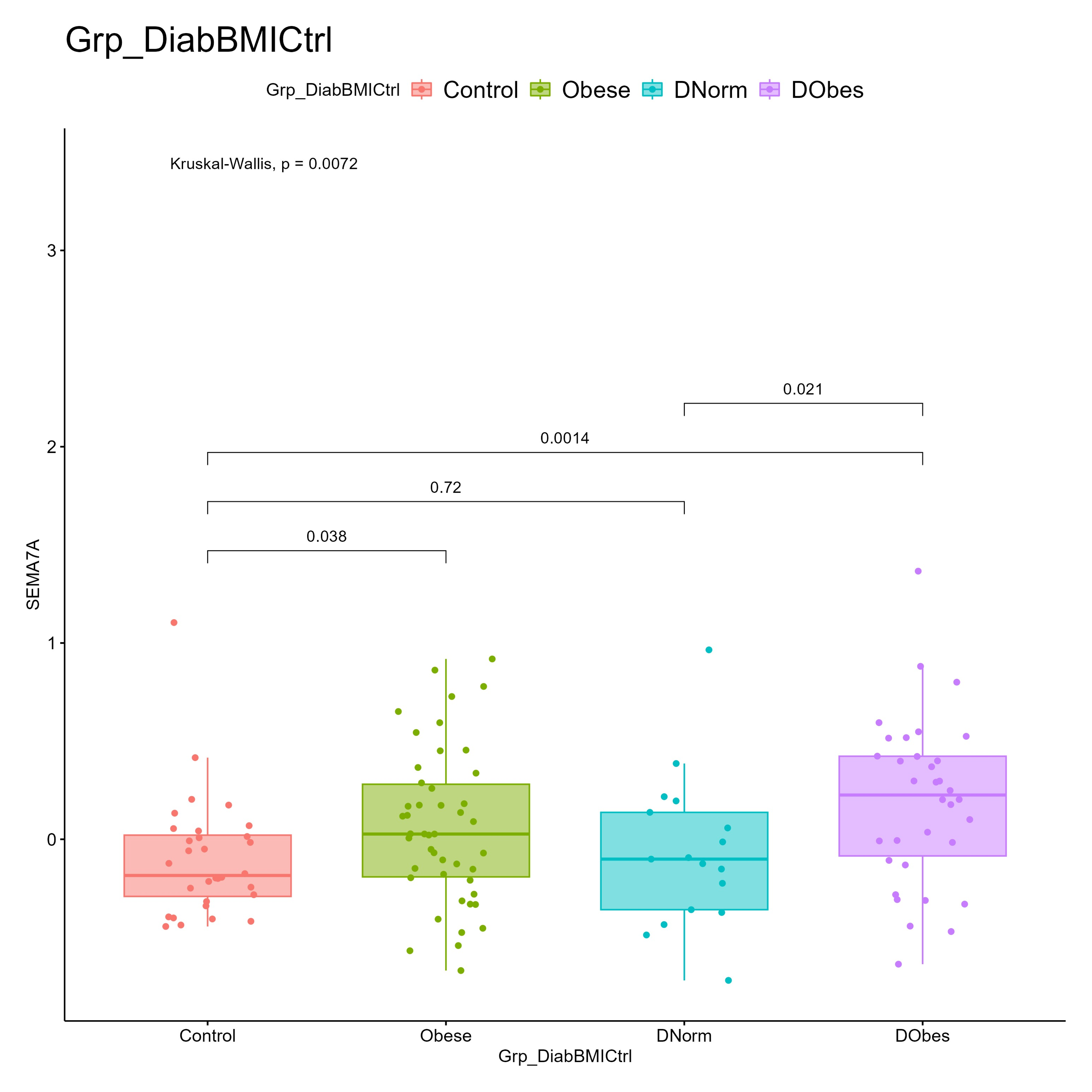
Grp_DiabBMICtrl
Grp_DiabBMICtrl
Control
Obese
DNorm
DObes
Kruskal-Wallis, p = 0.0072
0.021
0.0014
0.72
0.038
SEMA7A
3
2
1
0
Control
Obese
DNorm
DObes
Grp_DiabBMICtrl

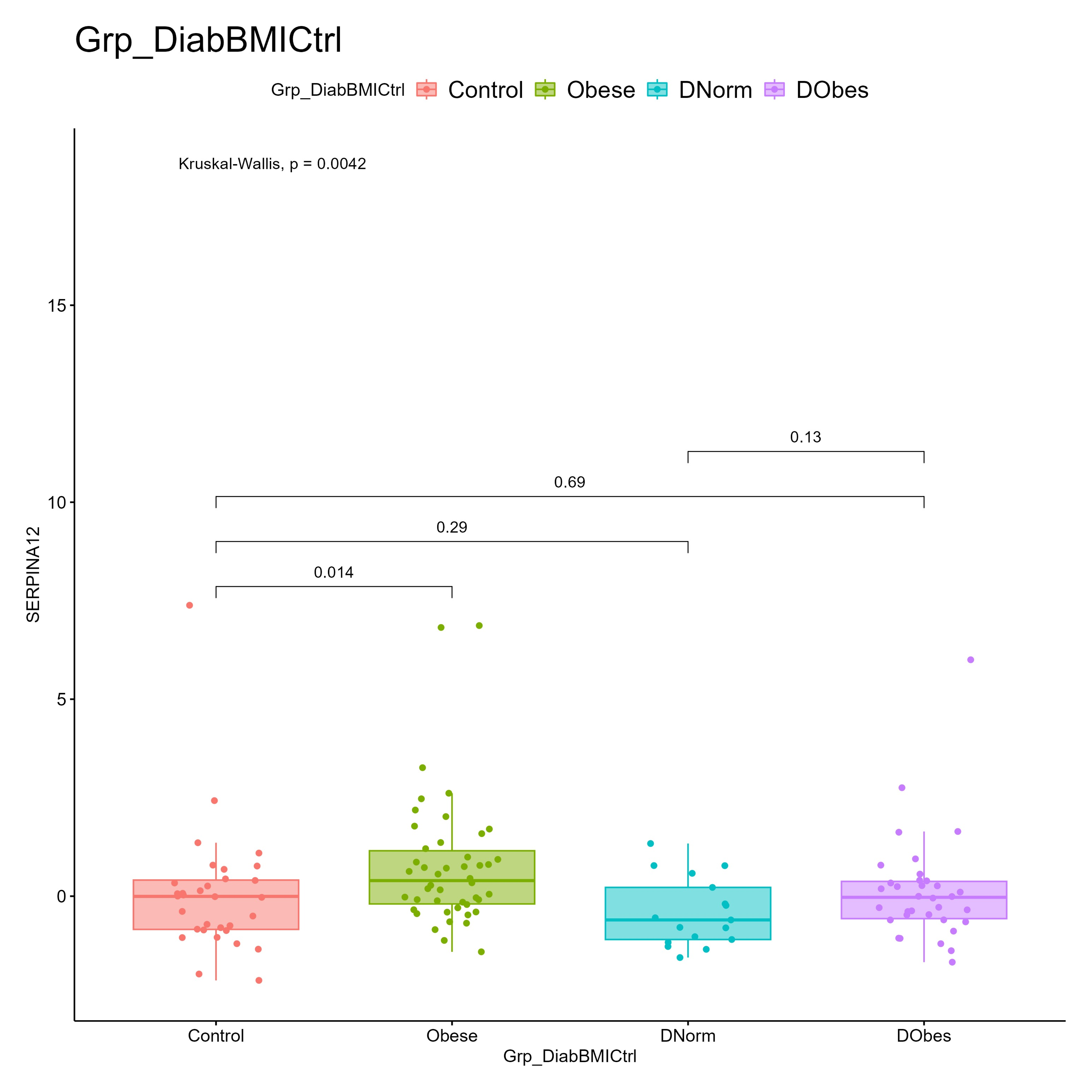

Grp_DiabBMICtrl
Grp_DiabBMICtrl
Control
Obese
DNorm
DObes
Kruskal-Wallis, p = 0.0042
0.13
0.69
0.29
0.014
15
10
5
0
SERPINA12
Control
Obese
DNorm
DObes
Grp_DiabBMICtrl

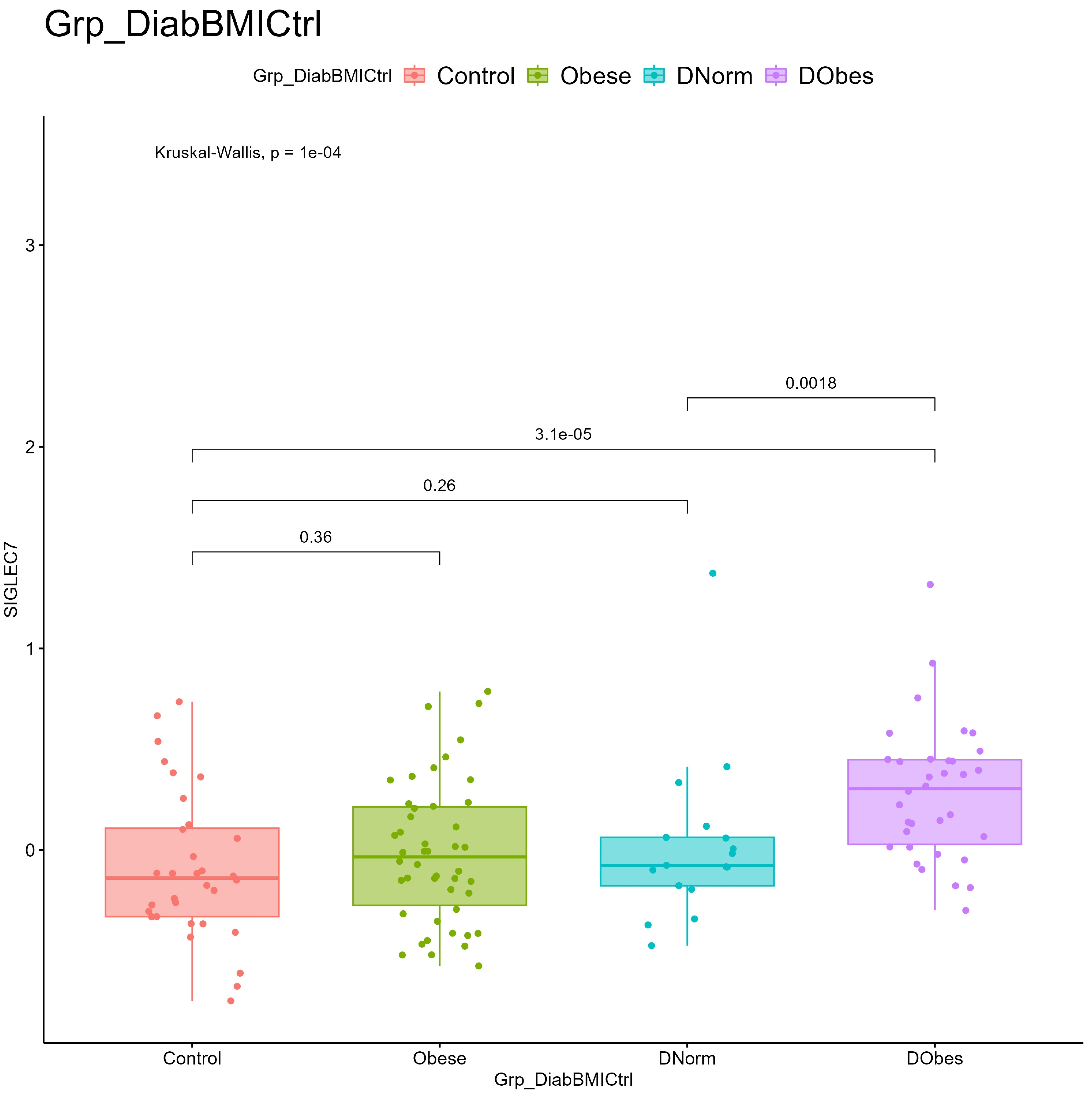

Grp_DiabBMICtrl
Grp_DiabBMICtrl
Control
Obese
DNorm
DObes
Kruskal-Wallis, p = 1e-04
0.0018
3.1e-05
0.26
0.36
SIGLEC7
3
2
1
0
Control
Obese
DNorm
DObes
Grp_DiabBMICtrl

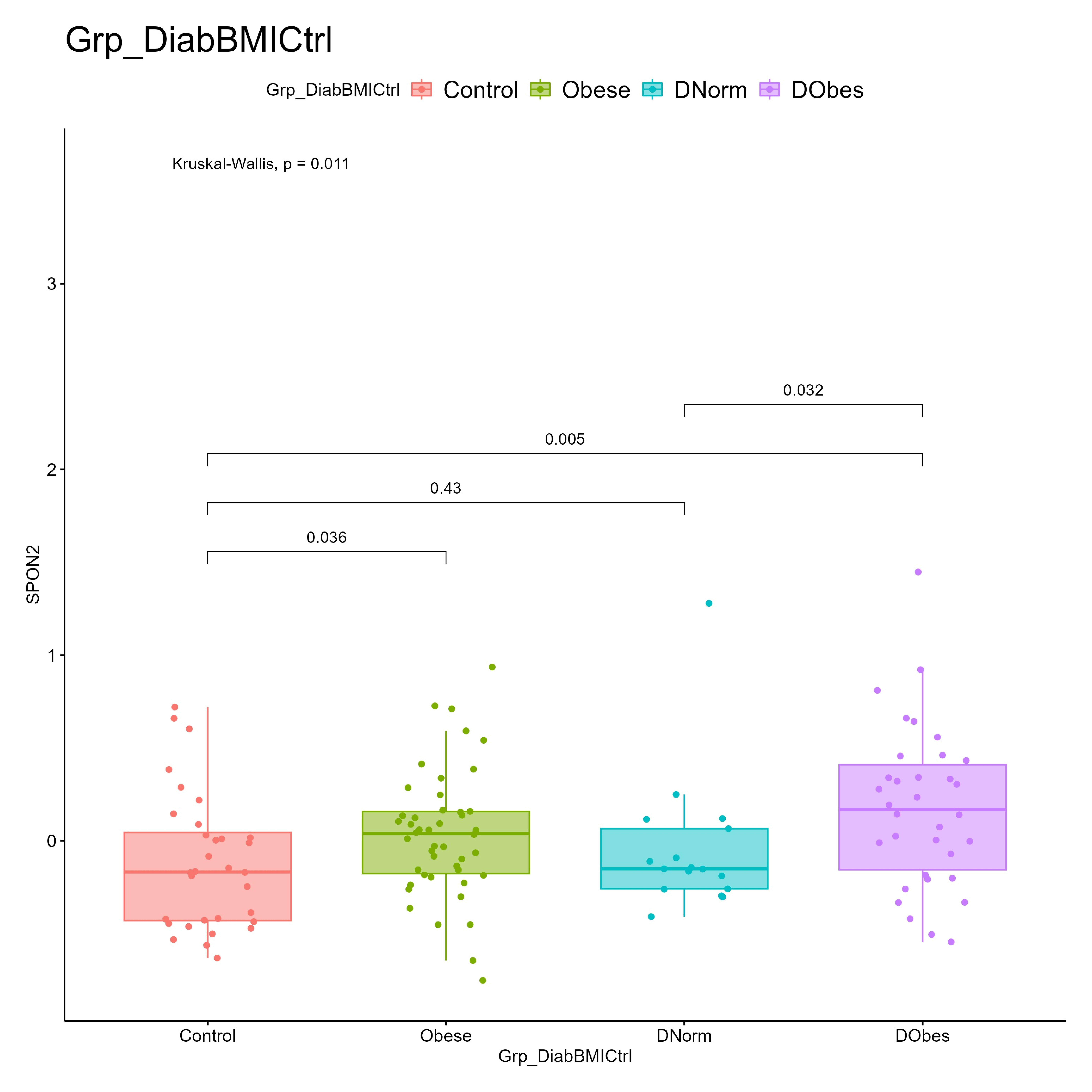

Grp_DiabBMICtrl
Grp_DiabBMICtrl
Control
Obese
DNorm
DObes
Kruskal-Wallis, p = 0.011
0.032
0.005
0.43
0.036
SPON2
3
2
1
0
Control
Obese
DNorm
DObes
Grp_DiabBMICtrl

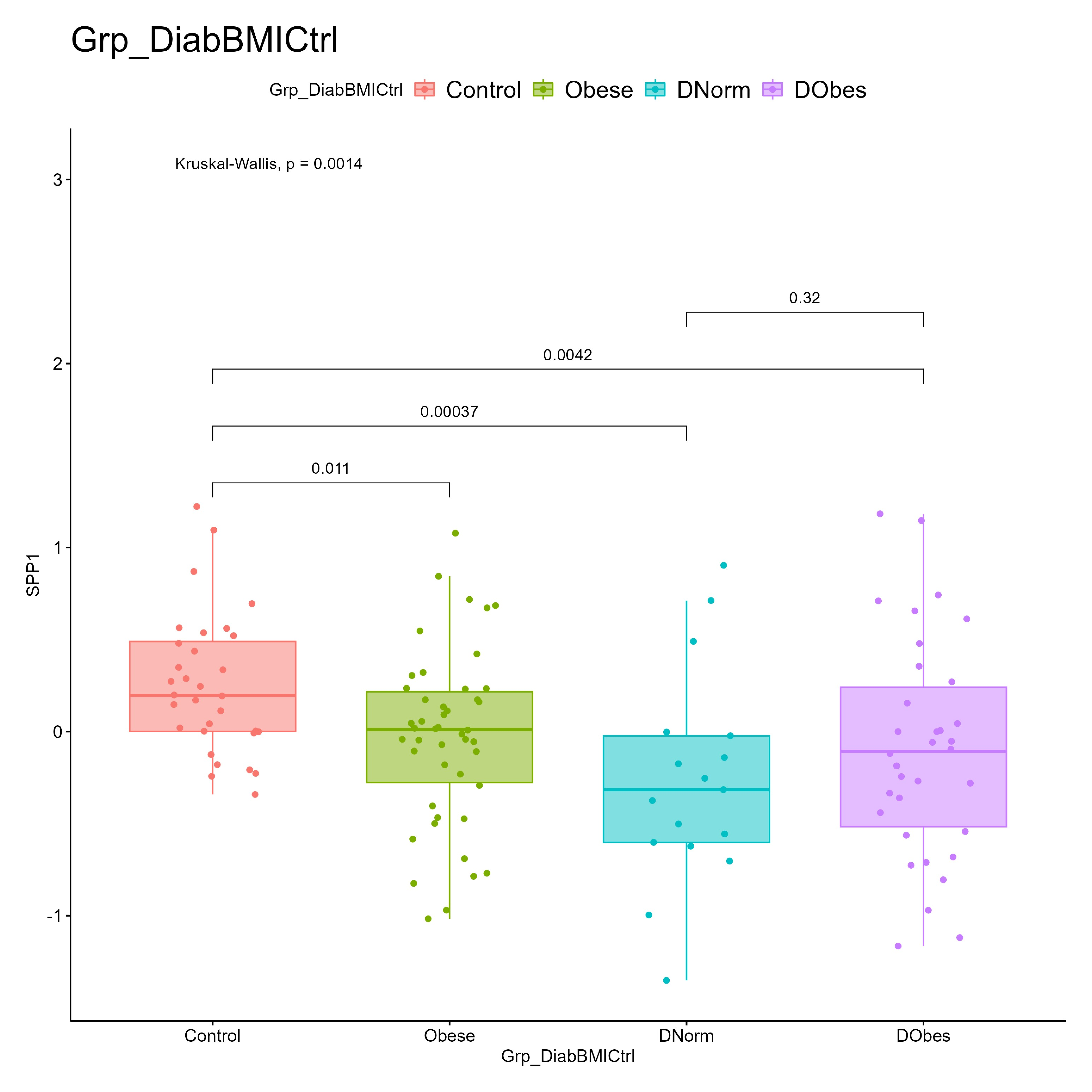

Grp_DiabBMICtrl
Grp_DiabBMICtrl
Control
Obese
DNorm
DObes
Kruskal-Wallis, p = 0.0014
0.32
0.0042
0.00037
0.011
SPP1
3
2
1
0
-1
Control
Obese
DNorm
DObes
Grp_DiabBMICtrl

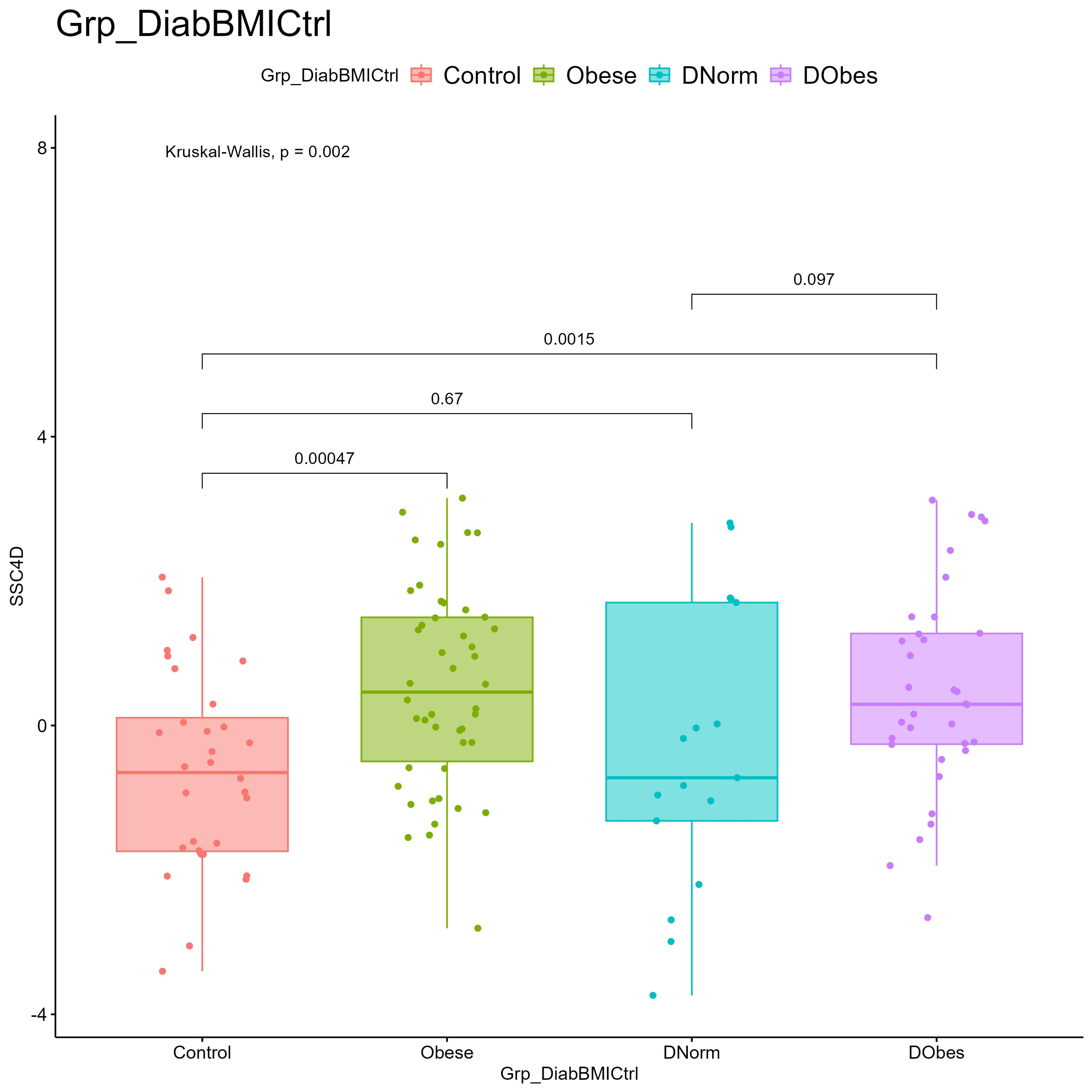
Grp_DiabBMICtrl
Grp_DiabBMICtrl
Control
Obese
DNorm
DObes
Kruskal-Wallis, p = 0.002
0.097
0.0015
0.67
0.00047
8
4
0
-4
SSC4D
Control
Obese
DNorm
DObes
Grp_DiabBMICtrl

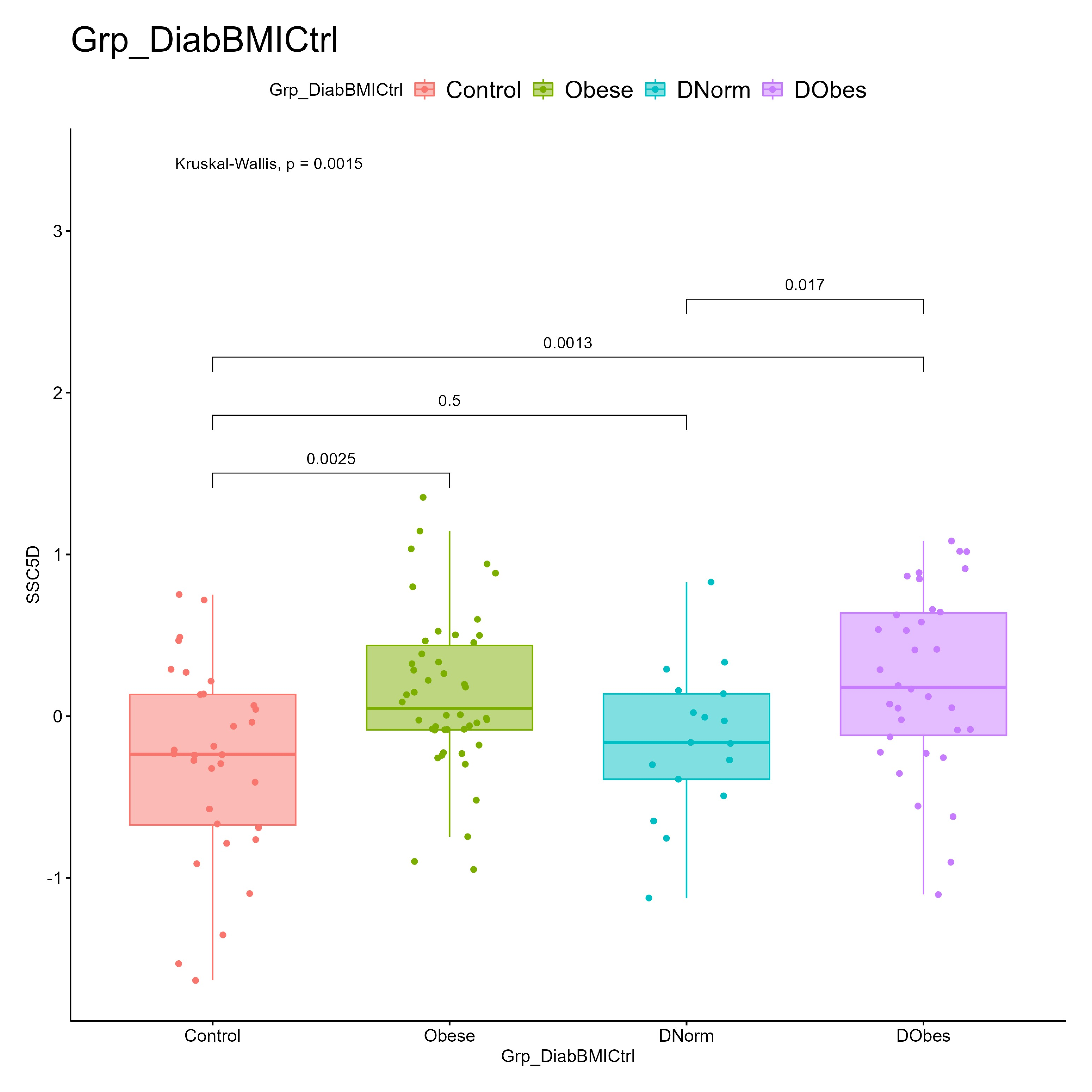

Grp_DiabBMICtrl
Grp_DiabBMICtrl
Control
Obese
DNorm
DObes
Kruskal-Wallis, p = 0.0015
0.017
0.0013
0.5
0.0025
3
2
1
0
-1
SSC5D
Control
Obese
DNorm
DObes
Grp_DiabBMICtrl

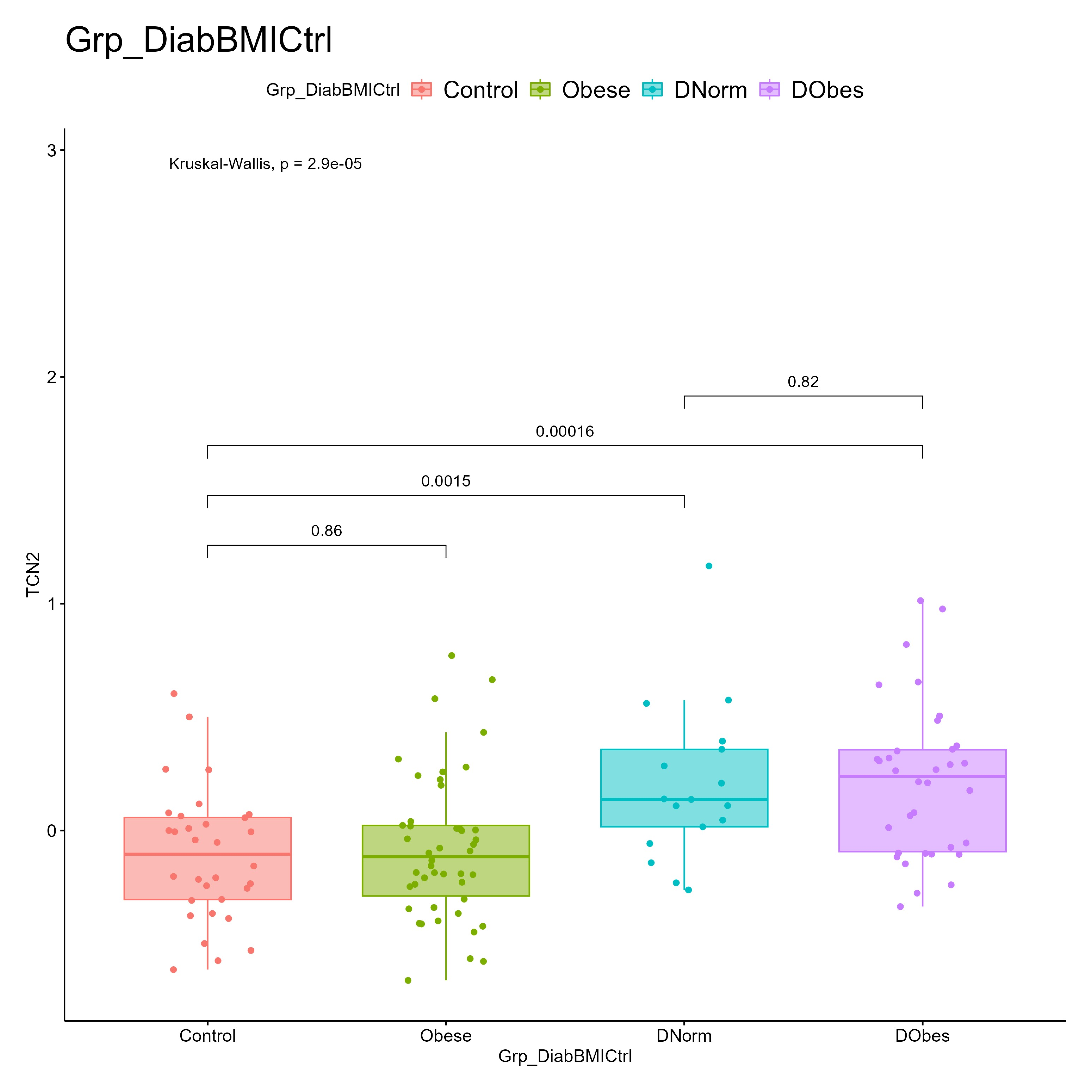

Grp_DiabBMICtrl
Grp_DiabBMICtrl
Control
Obese
DNorm
DObes
Kruskal-Wallis, p = 2.9e-05
0.82
0.00016
0.0015
0.86
TCN2
3
2
1
0
Control
Obese
DNorm
DObes
Grp_DiabBMICtrl

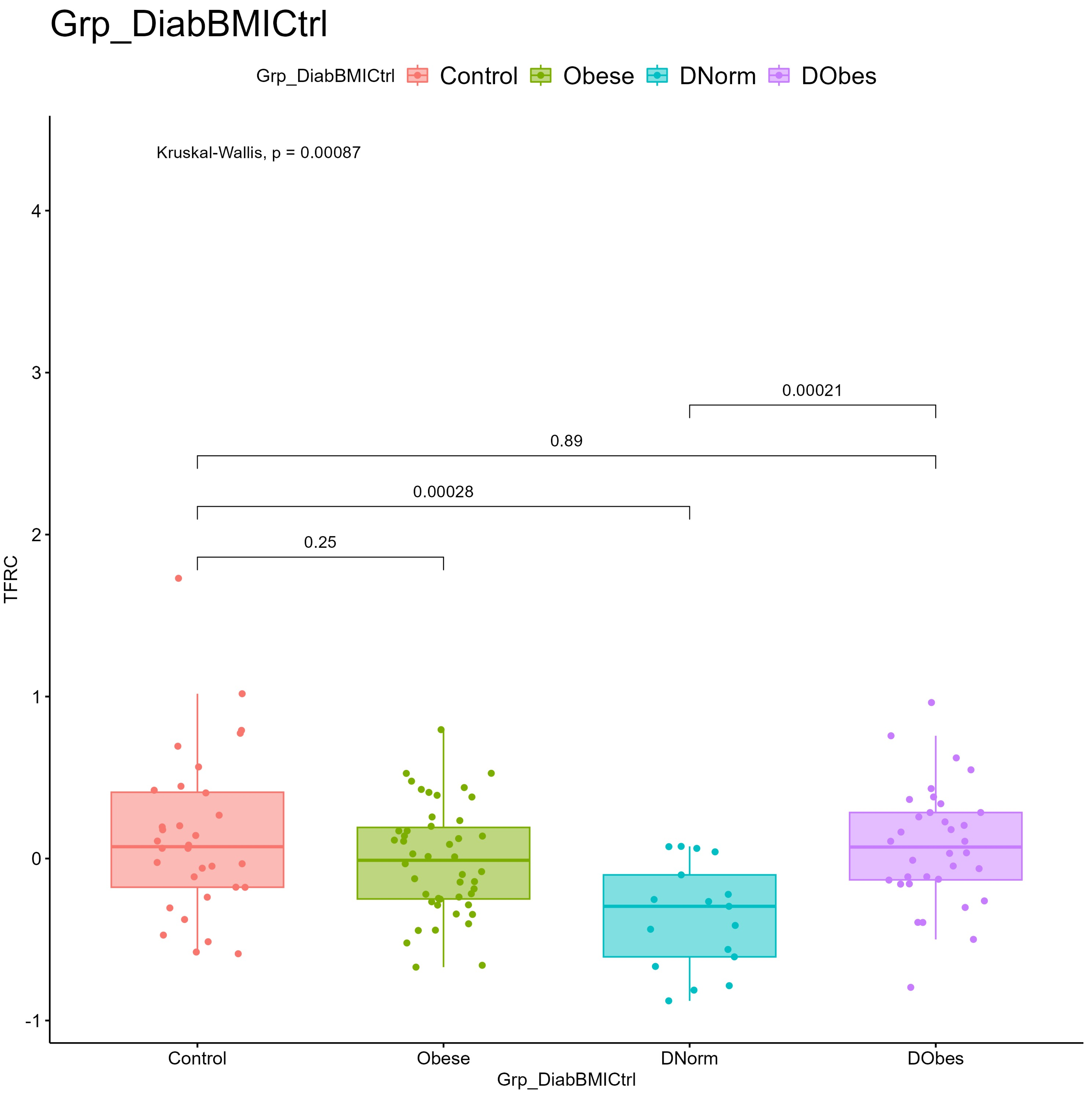

Grp_DiabBMICtrl
Grp_DiabBMICtrl
Control
Obese
DNorm
DObes
Kruskal-Wallis, p = 0.00087
0.00021
0.89
0.00028
0.25
TFRC
4
3
2
1
0
-1
Control
Obese
DNorm
DObes
Grp_DiabBMICtrl

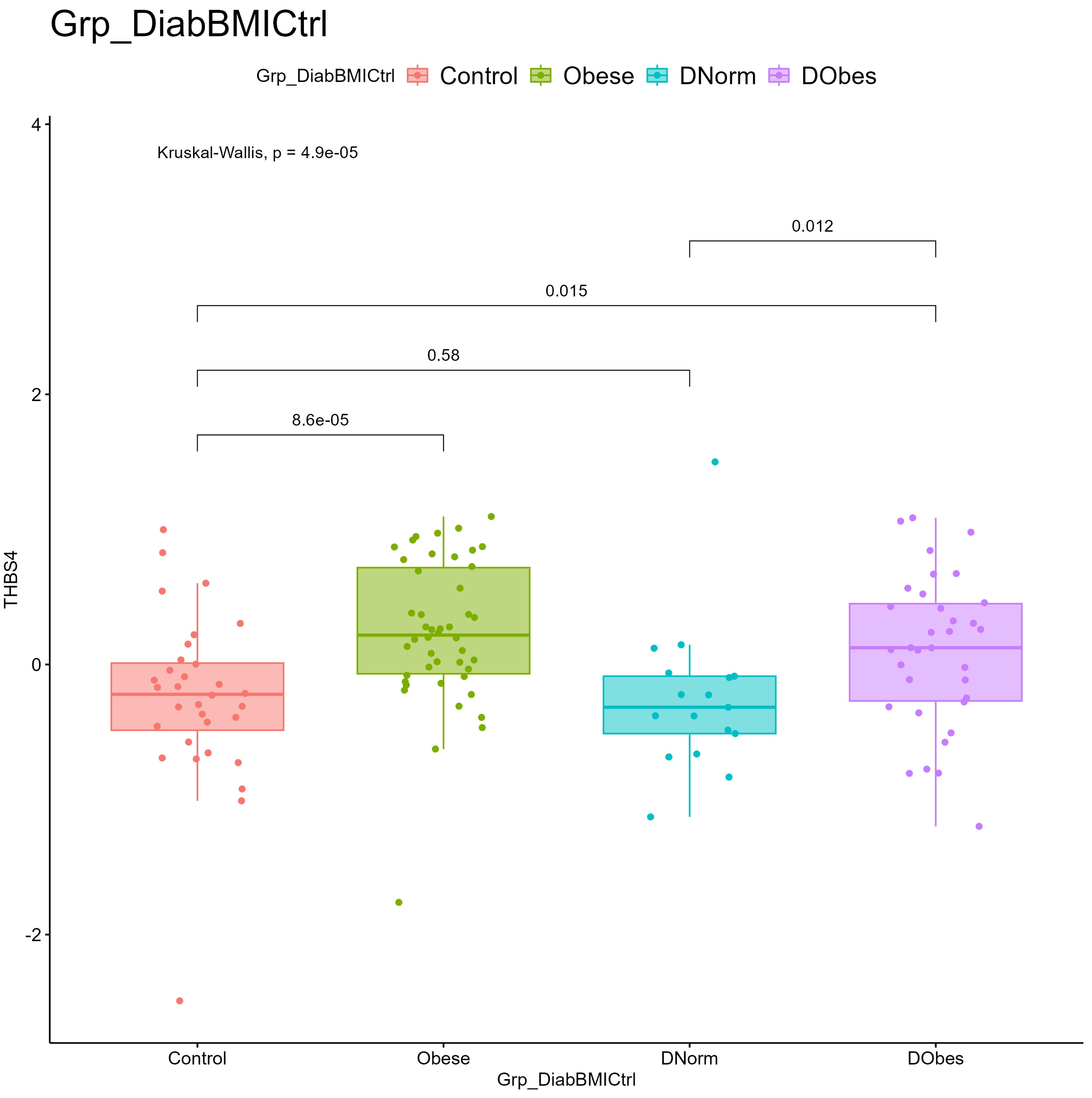

Grp_DiabBMICtrl
Grp_DiabBMICtrl
Control
Obese
DNorm
DObes
Kruskal-Wallis, p = 4.9e-05
0.012
0.015
0.58
8.6e-05
4
2
0
-2
THBS4
Control
Obese
DNorm
DObes
Grp_DiabBMICtrl

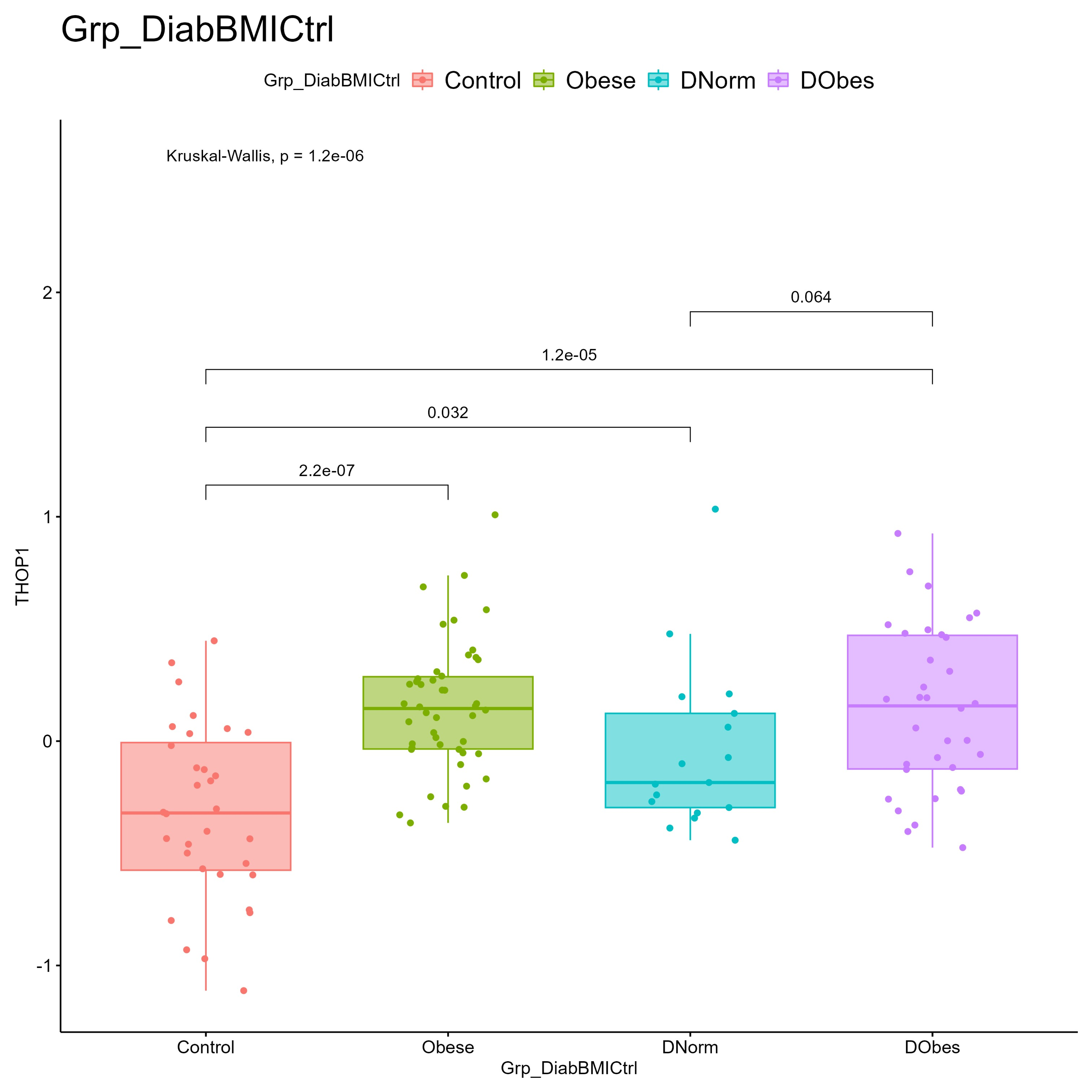

Grp_DiabBMICtrl
Grp_DiabBMICtrl
Control
Obese
DNorm
DObes
Kruskal-Wallis, p = 1.2e-06
0.064
1.2e-05
0.032
2.2e-07
THOP1
2
1
0
-1
Control
Obese
DNorm
DObes
Grp_DiabBMICtrl

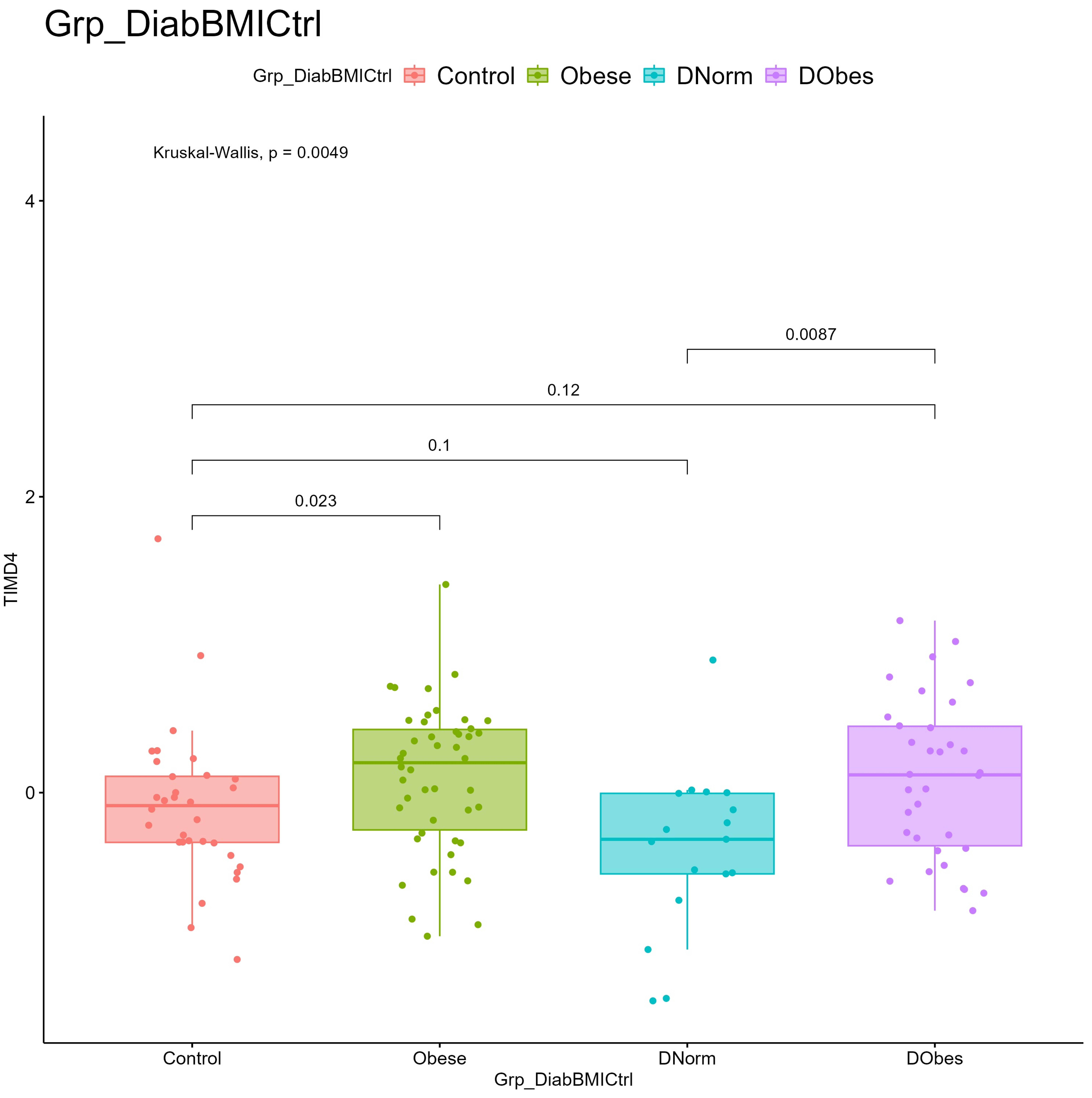

Grp_DiabBMICtrl
Grp_DiabBMICtrl
Control
Obese
DNorm
DObes
Kruskal-Wallis, p = 0.0049
0.0087
0.12
0.1
0.023
4
2
0
TIMD4
Control
Obese
DNorm
DObes
Grp_DiabBMICtrl

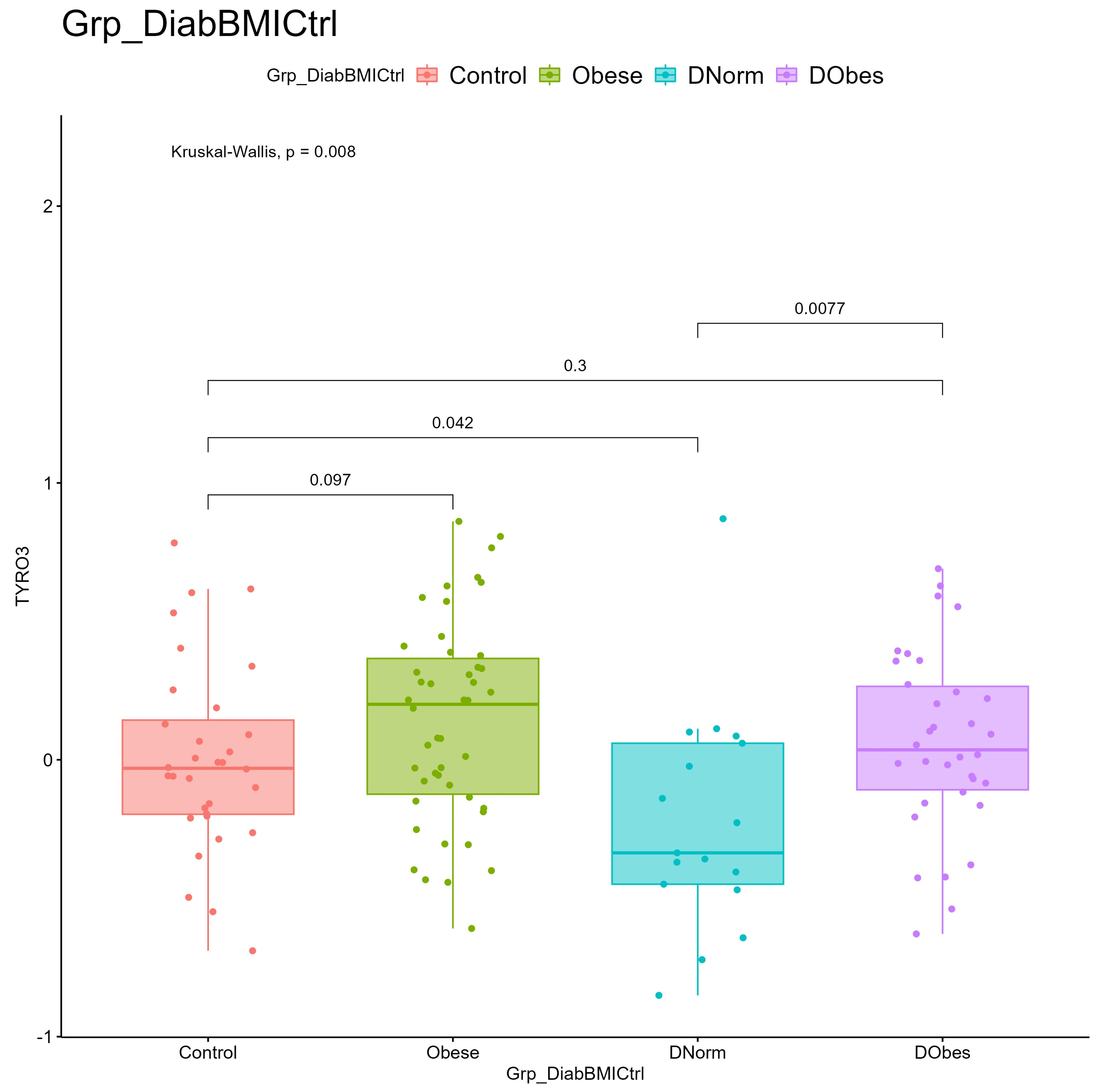

Grp_DiabBMICtrl
Grp_DiabBMICtrl
Control
Obese
DNorm
DObes
Kruskal-Wallis, p = 0.008
0.0077
0.3
0.042
0.097
TYRO3
2
1
0
-1
Control
Obese
DNorm
DObes
Grp_DiabBMICtrl

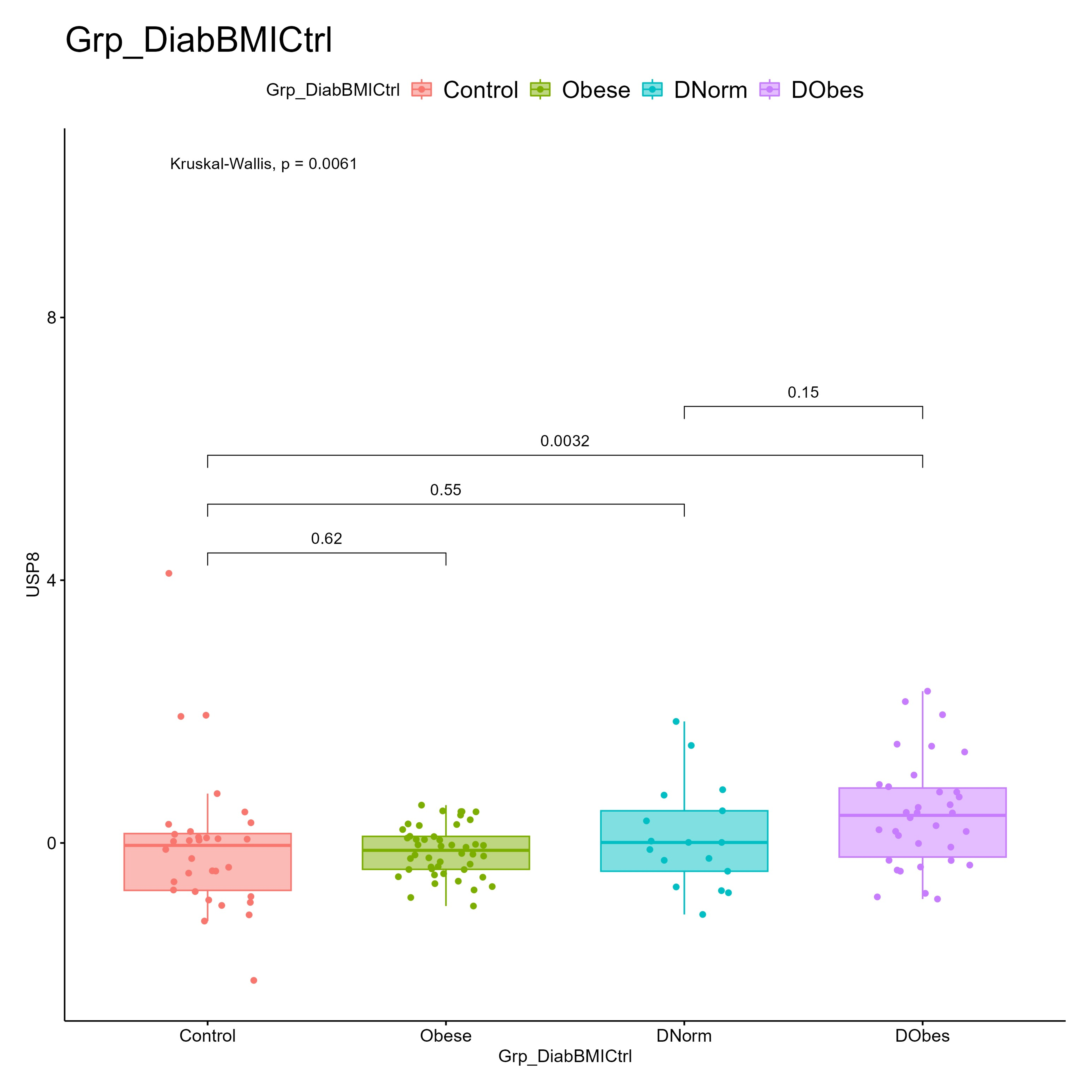

Grp_DiabBMICtrl
Grp_DiabBMICtrl
Control
Obese
DNorm
DObes
Kruskal-Wallis, p = 0.0061
0.15
0.0032
0.55
0.62
USP8
8
4
0
Control
Obese
DNorm
DObes
Grp_DiabBMICtrl

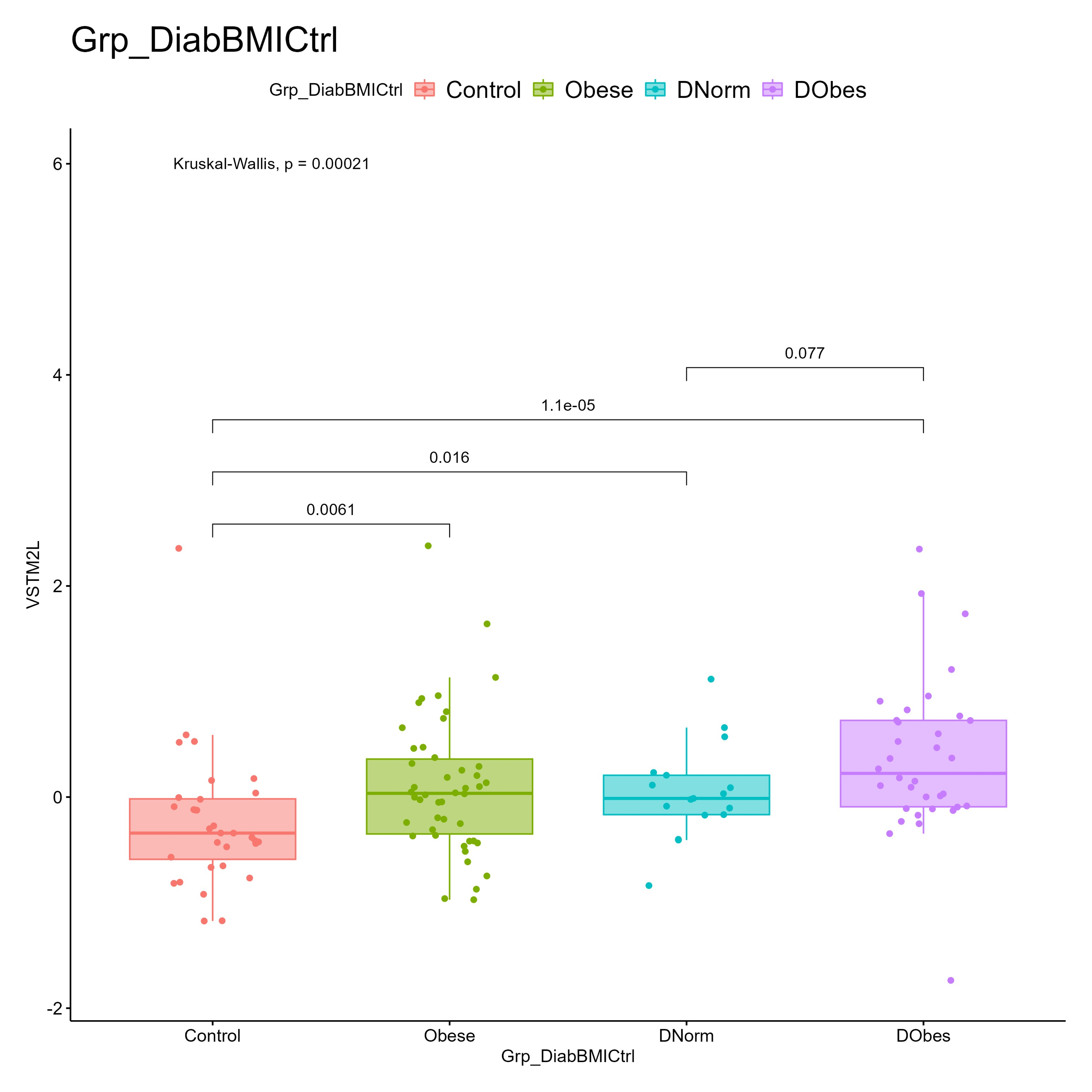

Grp_DiabBMICtrl
Grp_DiabBMICtrl
Control
Obese
DNorm
DObes
Kruskal-Wallis, p = 0.00021
0.077
1.1e-05
0.016
0.0061
6
4
2
0
-2
VSTM2L
Control
Obese
DNorm
DObes
Grp_DiabBMICtrl

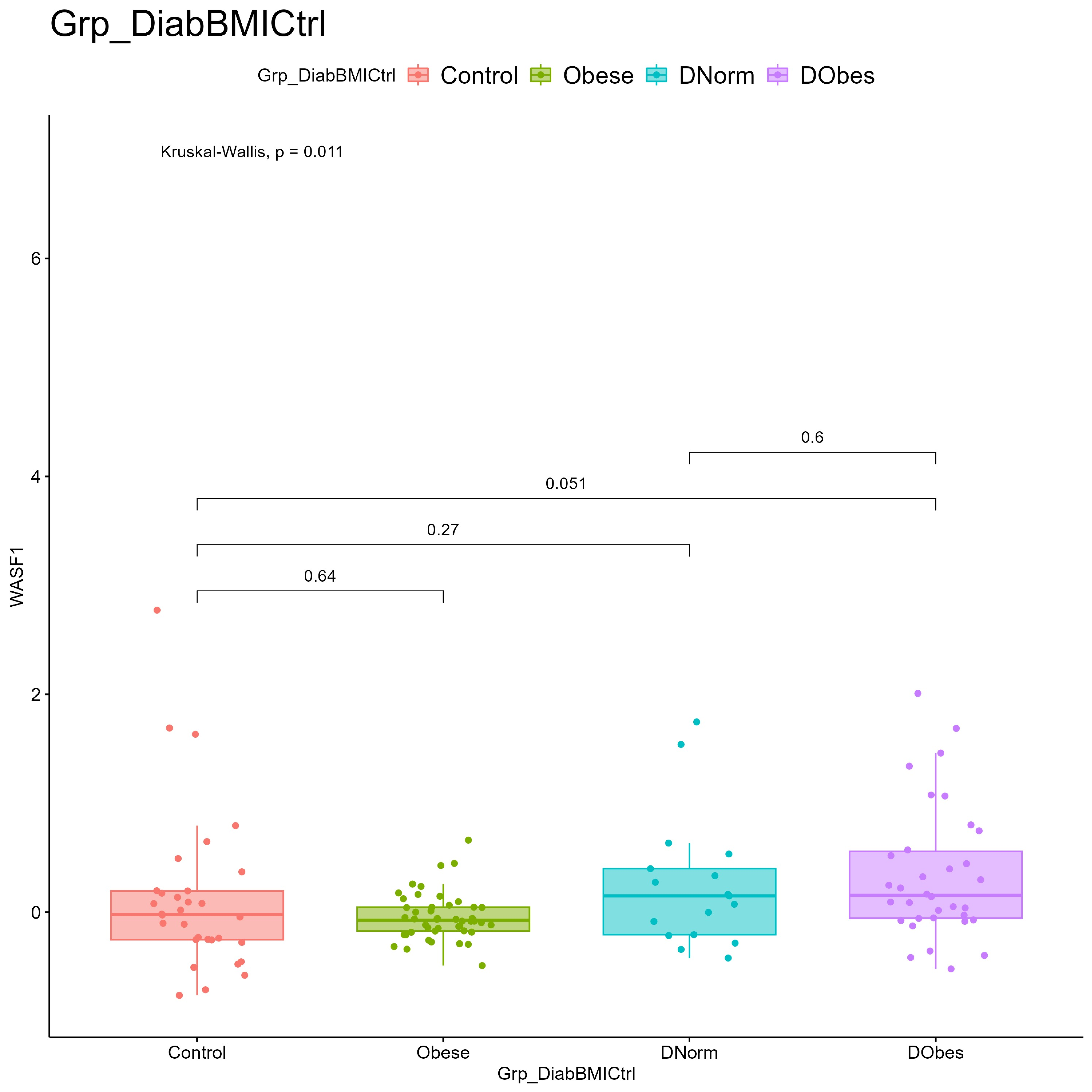

Grp_DiabBMICtrl
Grp_DiabBMICtrl
Control
Obese
DNorm
DObes
Kruskal-Wallis, p = 0.011
0.6
0.051
0.27
0.64
WASF1
6
4
2
0
Control
Obese
DNorm
DObes
Grp_DiabBMICtrl

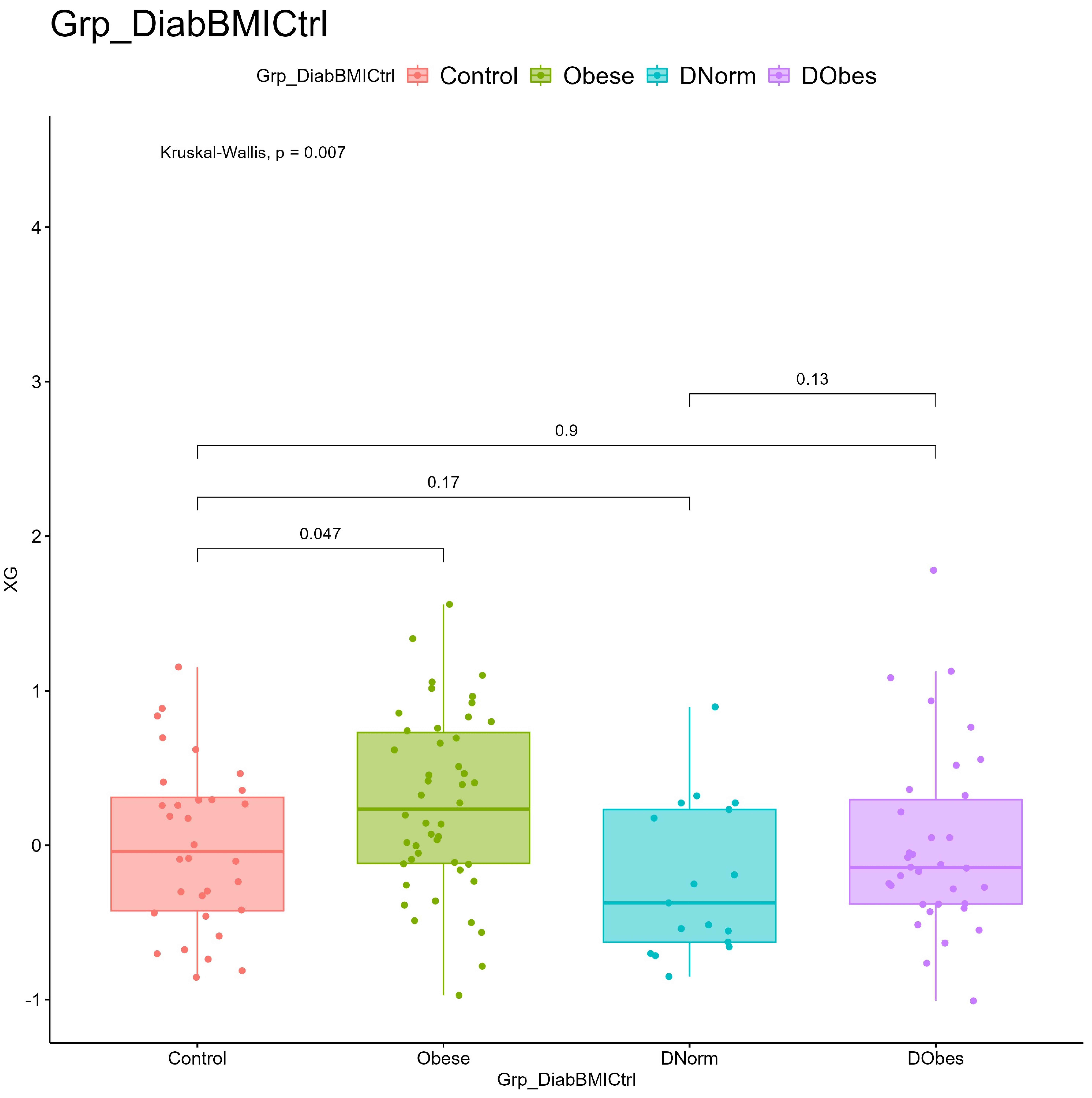
Grp_DiabBMICtrl
Grp_DiabBMICtrl
Control
Obese
DNorm
DObes
Kruskal-Wallis, p = 0.007
0.13
0.9
0.17
0.047
XG
4
3
2
1
0
-1
Control
Obese
DNorm
DObes
Grp_DiabBMICtrl
