# Supplementary figures and images for: Examination of the Complex Molecular Landscape in Obesity and Type 2 Diabetes
Source: Int J Mol Sci. 2024 Apr 27;25(9):4781. doi: 10.3390/ijms25094781 (PMC11084226; doi:10.3390/ijms25094781)

# Supplementary Figure 2

A

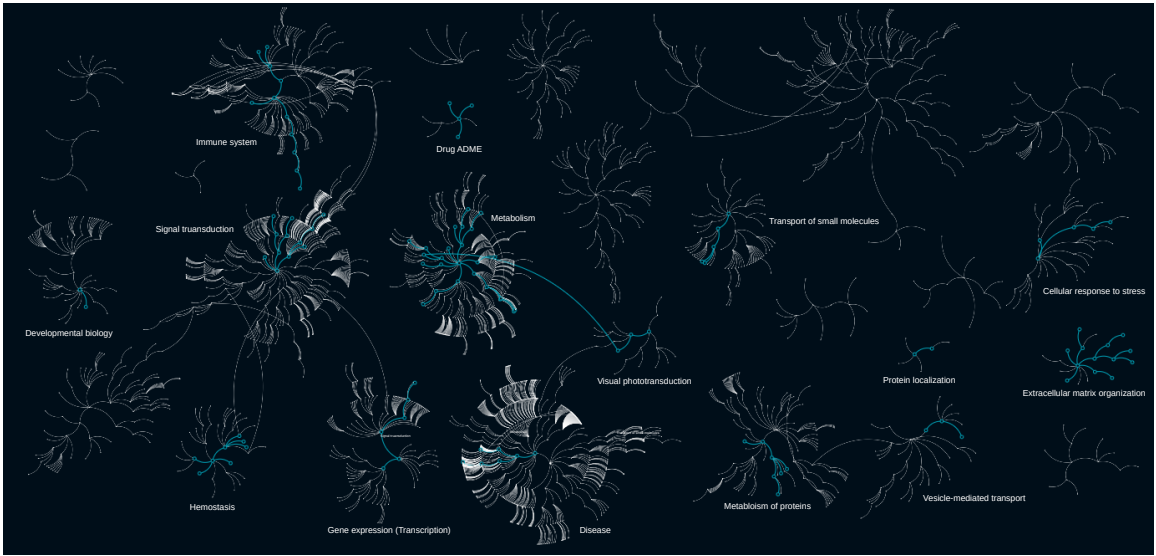

B

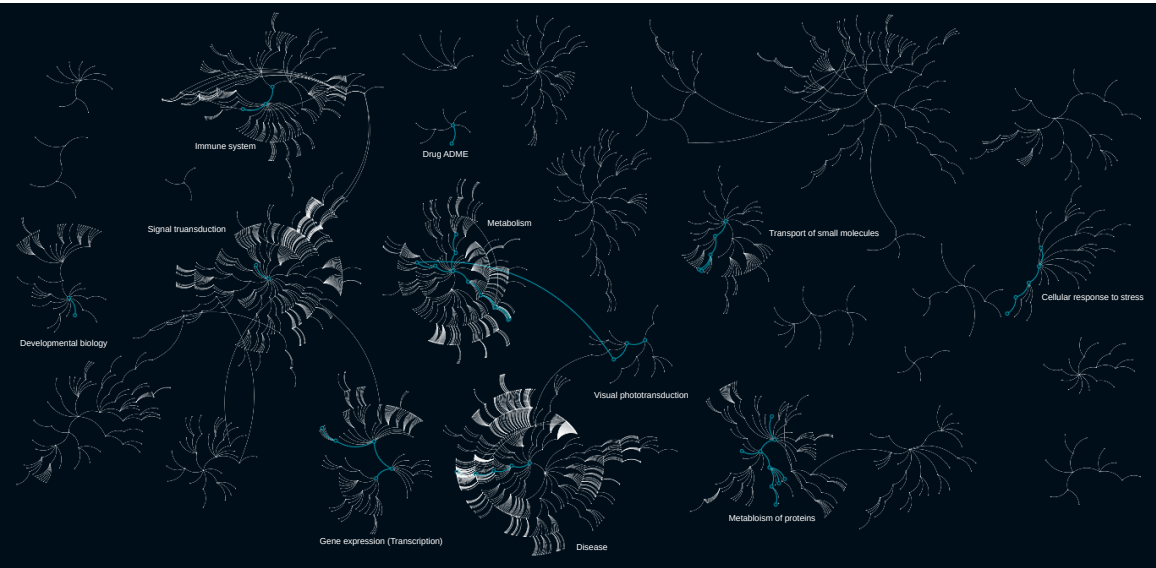

C

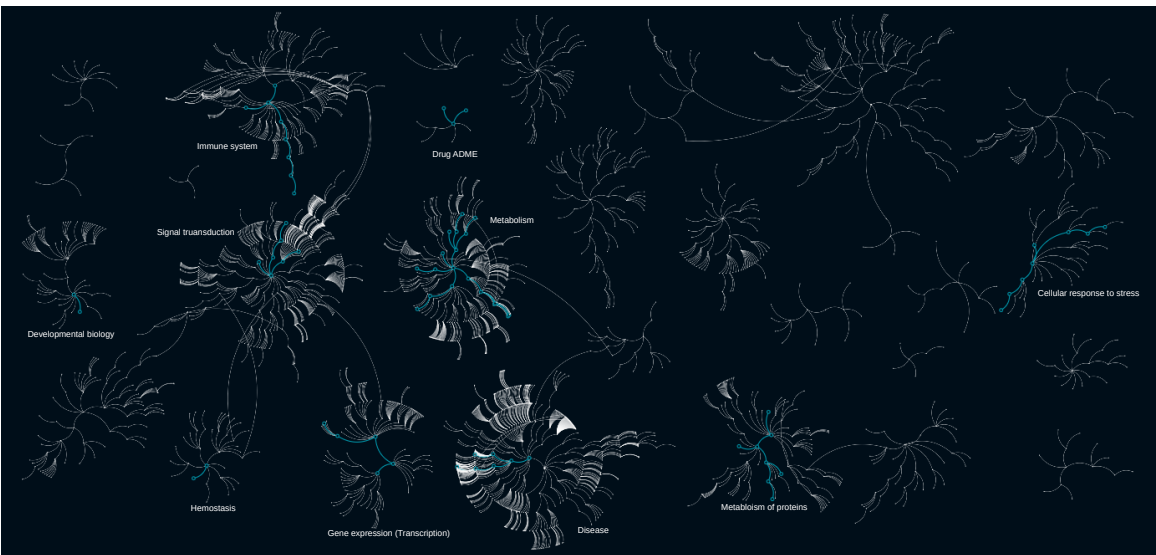

Supplement: Supplementary file 1 [file ijms-25-04781-s001.zip › FigureS2.pdf]
